# Impact of the COVID-19 Pandemic and the 2021 National Institute for Health and Care Excellence Guidelines on Public Perspectives Toward Myalgic Encephalomyelitis/Chronic Fatigue Syndrome: Thematic and Sentiment Analysis on Twitter (Rebranded as X)

**DOI:** 10.2196/65087

**Published:** 2025-05-21

**Authors:** Iliya Khakban, Shagun Jain, Joseph Gallab, Blossom Dharmaraj, Fangwen Zhou, Cynthia Lokker, Wael Abdelkader, Dena Zeraatkar, Jason W Busse

**Affiliations:** 1 McMaster University Hamilton, ON Canada

**Keywords:** myalgic encephalomyelitis, chronic fatigue syndrome, Twitter, sentiment analysis, post–COVID-19 condition, long COVID

## Abstract

**Background:**

Myalgic encephalomyelitis (ME), also referred to as chronic fatigue syndrome (CFS), is a complex illness that typically presents with disabling fatigue and cognitive and functional impairment. The etiology and management of ME/CFS remain contentious and patients often describe their experiences through social media.

**Objective:**

We explored public discourse on Twitter (rebranded as X) to understand the concerns and priorities of individuals living with ME/CFS, with a focus on (1) the COVID-19 pandemic and (2) publication of the 2021 UK National Institute for Health and Care Excellence (NICE) guidelines on the diagnosis and management of ME/CFS.

**Methods:**

We used the Twitter application programming interface to collect tweets related to ME/CFS posted between January 1, 2010, and January 30, 2024. Tweets were sorted into 3 chronological periods (pre–COVID-19 pandemic, post–COVID-19 pandemic, and post-UK 2021 NICE Guidelines publication). A Robustly Optimized Bidirectional Embedding Representations from Transformers Pretraining Approach (RoBERTa) language processing model was used to categorize the sentiment of tweets as positive, negative, or neutral. We identified tweets that mentioned COVID-19, the UK NICE guidelines, and key themes identified through latent Dirichlet allocation (ie, fibromyalgia, research, and treatment). We sampled 1000 random tweets from each theme to identify subthemes and representative quotes.

**Results:**

We retrieved 906,404 tweets, of which 427,824 (47.2%) were neutral, 369,371 (40.75%) were negative, and 109,209 (12.05%) were positive. Over time, both the proportion of negative and positive tweets increased, and the proportion of neutral tweets decreased (*P*<.001 for all changes). Tweets mentioning fibromyalgia acknowledged similarities with ME/CFS, stigmatization associated with both disorders, and lack of effective treatments. Treatment-related tweets often described frustration with ME/CFS labeled as mental illness, dismissal of concerns by health care providers, and the need to seek out “good physicians” who viewed ME/CFS as a physical disorder. Tweets on research typically praised studies of biomarkers and biomedical therapies, called for greater investment in biomedical research, and expressed frustration with studies suggesting a biopsychosocial etiology for ME/CFS or supporting management with psychotherapy or graduated activity. Tweets about the UK NICE guidelines expressed frustration with the 2007 version that recommended cognitive behavioral therapy and graded exercise therapy, and a prolonged campaign by advocacy organizations to influence subsequent versions. Tweets showed high acceptance of the 2021 UK NICE guidelines, which were seen to validate ME/CFS as a biomedical disease and recommended against graded exercise therapy. Tweets about COVID-19 often noted overlaps between post–COVID-19 condition and ME/CFS, including claims of a common biological pathway, and advised there was no cure for either condition.

**Conclusions:**

Our findings suggest research is needed to inform how best to support patients’ engagement with evidence-based care. Furthermore, while patient involvement with ME/CFS research is critical, unmanaged intellectual conflicts of interest may threaten the trustworthiness of research efforts.

## Introduction

### Background

Myalgic encephalomyelitis (ME), also known as chronic fatigue syndrome (CFS) [1], is characterized by persistent fatigue, cognitive dysfunction, and impaired daily functioning [2]. The global prevalence of ME/CFS has been estimated at 0.89% (95% CI 0.6%-1.33%), and it is more common among women aged 40 to 50 years [3]. Despite the prevalence and debilitating nature of ME/CFS, it remains poorly understood due to uncertain pathophysiology, nonspecific symptoms, and limited education and acceptance by the medical community [4].

There have been 2 recent events that may have affected public perceptions toward ME/CFS. The first was the reversal of a 2007 clinical practice recommendation for use of graded exercise in the management of ME/CFS by the UK National Institute for Health and Care Excellence (NICE) in 2021. The original recommendation in favor of exercise was seen by some patient advocacy groups as stigmatizing their condition as psychosocial [5,6]. Second, the COVID-19 pandemic, and resulting millions of recovered patients who developed post–COVID19 condition (PCC; also known as “long COVID”), a condition with considerable overlap with ME/CFS [7]. The emergence of PCC has reignited both research and clinical attention regarding postinfectious fatigue syndromes [8,9].

Lived experiences are invaluable sources of information for guiding research [10], and people living with ME/CFS report higher levels of web-based activity compared with other patient groups [11,12]. Social media is highly valued among individuals with ME/CFS to connect with other patients [13]. Data from social media have been used to model public sentiment and perceptions of health issues, especially relating to COVID-19 [14] and PCC [15,16]. However, while studies have suggested similarities between PCC and ME/CFS [17,18], no study has specifically analyzed social media information on ME/CFS.

To date, numerous frameworks for sentiment analysis have been explored [9,14]. Pretrained encoder transformers, such as Bidirectional Embedding Representations from Transformers (BERT) and its variants have demonstrated potential in accurate sentiment assessment due to sophisticated contextual understanding from multihead self-attention [15-17]. Furthermore, the transformer architecture significantly improves training efficiency, allowing for large pretrained models, and requires minimal preprocessing due to the ability of complex neural networks to automatically capture hidden patterns during training [18].

For topic modeling and thematic analysis, term frequency and latent Dirichlet allocation (LDA) have emerged as widely used approaches. Term frequency is a simple metric that quantifies how often a particular word appears in a dataset, providing insights into commonly discussed topics [19]. LDA is a generative probabilistic model that assumes each document is a mixture of topics, and each topic is characterized by a distribution of words [20]. By uncovering these distributions, LDA groups terms with similar semantic meaning, making it easier to discern overarching topics and patterns and enabling researchers to identify latent themes within large text datasets [21].

### This Study

To examine the public discourse on Twitter (rebranded as X in July 2023), we leveraged a BERT variant for sentiment analysis and term frequency and LDA for topic modeling and thematic analysis to understand the sentiments, concerns, and priorities of individuals living with ME/CFS and associated interest holders. By addressing these knowledge gaps, this study helps inform the use of social media analysis in understanding complex disorders, such as ME/CFS.

## Methods

### Overview

**Figure 1:**
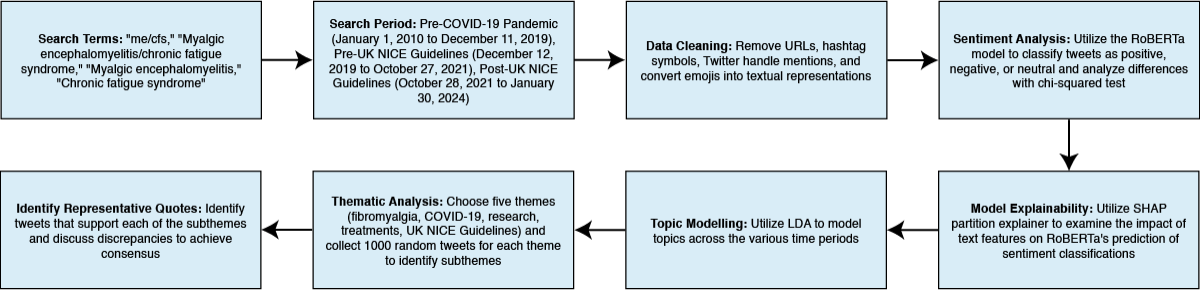
Flowchart summarizing the methodology used to retrieve tweets and conduct analyses. LDA: latent Dirichlet allocation; NICE: National Institute for Health and Care Excellence; RoBERTa; Robustly Optimized Bidirectional Embedding Representations from Transformers Pretraining Approach; SHAP: Shapley Additive Explanations.

We conducted a retrospective observational study of tweets. Our research approach consisted of seven steps: (1) developing Twitter search terms, (2) establishing a period from which tweets would be collected, (3) using the Twitter application programming interface (API) to collect tweets within the defined search period, (4) processing tweets to enhance the accuracy of data analyses, (5) performing sentiment analysis using a Robustly Optimized BERT Pretraining Approach (RoBERTa), (6) identifying themes among tweets through word frequency, and (7) collecting and further analyzing 1000 random tweets for each theme to identify subthemes and representative quotes (Figure 1) [22].

### Data Collection

The search terms used to collect tweets were “me/cfs,” “myalgic encephalomyelitis/chronic fatigue syndrome,” “myalgic encephalomyelitis,” and “chronic fatigue syndrome.” We excluded non-English tweets and retweets.

The search period for tweet collection was January 1, 2010, to January 30, 2024. The full-archive search end point from the Twitter API allows users to retrieve publicly available tweets posted since March 2006 [23]. We were aware of 2 key events relevant to ME/CFS during our search period: the onset of the COVID-19 pandemic and subsequent recognition of PCC (which has substantial overlap with ME/CFS) [18], and the release of the 2021 UK NICE guidelines for the diagnosis and management of ME/CFS [24]. As such, we grouped retrieved tweets into the following three periods for analysis: (1) pre–COVID-19 pandemic (January 1, 2010, to December 11, 2019), (2) post–COVID-19 pandemic (December 12, 2019, to October 27, 2021), and (3) post-UK NICE guidelines publication (October 28, 2021, to January 30, 3024). December 11, 2019, was chosen as the last day of the pre–COVID-19 period as the first report of the SARS-CoV-2 virus was on December 12, 2019 [25]. Similarly, October 28, 2021, was chosen as the first day to begin collecting tweets for the post-UK 2021 NICE guidelines publication dataset because the new guidelines were originally announced on that day, although they were officially published on October 29, 2021 [26].

### Data Preprocessing

For our sentiment analysis, modifications to tweets included removal of URLs, hashtag symbols, and Twitter handle mentions. Emojis were converted to their textual representations as they convey sentiment. For topic modeling, URLs, hashtag symbols, Twitter handle mentions, emojis, and original search terms were removed, and all remaining words were uncased and lemmatized using wordnet part-of-speech tagging. Empty tweets were excluded from analysis.

### Sentiment Analysis

We used the natural language processing (NLP) capabilities of RoBERTa to conduct sentiment analysis of the cleaned tweets. RoBERTa, an iteration of BERT, was selected over its predecessor due to its robust performance and notable effectiveness in understanding short-text formats, such as tweets [27-29]. RoBERTa was trained on approximately 58 million tweets and fine-tuned for sentiment analysis with the TweetEval benchmark [30]. With Softmax activation for the dense output layer, RoBERTa generated probability scores for each tweet and categorized them into 3 mutually exclusive sentiment classes: negative, neutral, or positive. The difference between the proportion of sentiments across all possible comparison between the 3 periods (pre–COVID-19 pandemic vs post–COVID-19 pandemic, post–COVID-19 pandemic vs post-UK NICE guidelines publication, pre–COVID-19 pandemic vs post-UK NICE guidelines publication, and pre–COVID-19 pandemic vs post–COVID-19 pandemic vs post-UK NICE guidelines publication) were evaluated using a chi-square test, where *P*<.05 indicates statistical significance.

### Model Explainability

Model explainability for RoBERTa was assessed using Shapley Additive Explanations (SHAP) Partition Explainer [31-33]. SHAP values were computed for the predicted probability of each tweet individually. Subsequently, text features were uncased and trimmed to remove leading and trailing white spaces. The 15 most important positive and negative features by the mean SHAP values across different periods were presented. We analyzed unique text features with ≥100 occurrences.

### Topic Modeling

Topics across timeframes were modeled using LDA. The models were trained with 10 passes and a random seed of 42, and 6 topics were generated.

### Identification of Tweet Themes

On the basis of key events during the search period, our research questions, and the most frequent terms—excluding words used in search terms, we identified themes to explore in the retrieved tweets. For each theme, the corresponding tweets were each assigned a unique number, and a random number generator (Microsoft Excel version 2406) was used to select a random sample of tweets. A preliminary coding scheme was developed through inductive coding of a random sample of 100 tweets for each theme. This exercise was completed independently by 5 analysts. After completion, a new random sample of 1000 tweets were acquired for each theme, and the coding scheme was applied to this sample independently by teams of 2 to 3 reviewers. Each team included a physician-in-training and a methodologist with training in qualitative research. The research team met throughout the coding process to discuss and revise the coding scheme, as necessary. Subthemes represented in fewer than 20 tweets were discarded. Reviewers discussed any discrepancies to achieve consensus.

### Hardware and Software

All SHAP values were calculated in parallel using multiple NVIDIA V100 Volta (32G HBM2 memory) each with 8 central processing unit threads and 40 GB of system memory provided by the Digital Alliance of Canada. All other analyses were conducted using an NVIDIA RTX 2070 (8 GB graphics double data rate 6 [GDDR6] memory) with 32 central processing unit threads and 64 GB of system memory.

The unprocessed dataset and accompanying code are available on the web [34]. Tweets were queried and fetched using *tweepy* and the *GET/2/tweets/search/all* end point in Twitter API (version 2). Data management and preprocessing were handled with *pandas*, *re*, and *nltk*. Sentiment analysis was performed using Hugging Face *transformers*, while *shap* provided interpretability insights. Statistical analyses leveraged *numpy*, *scipy*, *statsmodels*, and *sklearn*. For topic modeling, the *genism* library was used. All visualizations were generated with *matplotlib* and *seaborn*. Twitter thematic analysis was completed using Microsoft Excel.

### Ethical Considerations

This study ensured anonymity and confidentiality of Twitter users by removing the Twitter handles associated with all representative tweets reported. The requirement for ethics review was waived by the Hamilton Integrated Research Ethics Board at McMaster University because all tweets were publicly available.

## Results

### Characteristics of the Dataset

After processing, the Twitter API retrieved 906,404 tweets relevant to ME/CFS during our search period. This included 570,723 (62.96%) tweets spanning from January 1, 2010, to December 11, 2019 (pre–COVID-19 pandemic group); 139,486 (15.38%) tweets spanning from December 12, 2019, to October 27, 2021 (post–COVID-19 pandemic group); and 196,195 (21.64%) tweets spanning from October 28, 2021, to January 30, 2024 (post-UK NICE guidelines publication group).

### Sentiment Analysis

Overall, tweets related to ME/CFS were predominantly neutral (n=427,824, 47.2%) or negative (n=369,371, 40.8%), with only 12% of tweets (n=109,209) classified as conveying positive sentiments (Table 1). Over the 3 periods of interest, we found the proportion of negative tweets increased from 36.7% to 49.9%, the proportion of positive tweets increased from 11.1% to 14.3%, and neutral tweets decreased. Differences in sentiment proportions were significantly different across all comparisons (*P*<.001).

**Table 1:** Sentiment proportions.

Period	Positive tweets, n (%)	Neutral tweets, n (%)	Negative tweets, n (%)
Pre–COVID-19 pandemic (n=570,723)	63,094 (11.1)	298,411 (52.3)	209,218 (36.7)
Post–COVID-19 pandemic (n=139,486)	17,983 (12.9)	59,173 (42.4)	62,330 (44.7)
Post-UK NICE guidelines publication (n=196,195)	28,132 (14.3)	70,240 (35.8)	97,823 (49.9)
Total (N=906,404)	109,209 (12)	427,824 (47.2)	369,371 (40.8)

### Model Explanations

Across all periods, the importance of 99,231,570 individual features were analyzed, of which 29,558 (0.03%) were unique and 8872 (0.009%) demonstrated 100 or more occurrences. The top 15 most important features by the mean SHAP values for each sentiment are presented in Table 2. Tokens, such as “excited,” “enjoyed,” and “appreciate” increased the probability of positive sentiment and decreased the probability of neutral and negative sentiments. Tokens with obvious negative connotations, including “disgusting,” “sucks,” and “horrible” increased negative sentiment probabilities. Explanations tweets across different periods are provided in Figures S1-S18 in Multimedia Appendix 1. Sentimentally important features were similar across different periods.

**Table 2:** Shapley Additive Explanations (SHAP) for all tweets.

Token features			SHAP value, mean (95% CI)
**Positive features for positive sentiment**			
	excited	0.480 (0.467 to 0.493)	
	exciting	0.432 (0.418 to 0.446)	
	enjoyed	0.367 (0.339 to 0.394)	
	appreciate	0.314 (0.306 to 0.323)	
	congratulations	0.297 (0.279 to 0.314)	
	appreciated	0.286 (0.275 to 0.297)	
	delighted	0.272 (0.253 to 0.291)	
	enjoying	0.268 (0.244 to 0.291)	
	lovely	0.251 (0.242 to 0.26)	
	excellent	0.231 (0.225 to 0.237)	
	liked	0.226 (0.221 to 0.232)	
	blessed	0.226 (0.205 to 0.247)	
	proud	0.218 (0.208 to 0.227)	
	beautiful	0.216 (0.205 to 0.227)	
	thankful	0.212 (0.197 to 0.227)	
**Negative features for positive sentiment**			
	bye	–0.082 (–0.094 to –0.069)	
	deprived	–0.072 (–0.075 to –0.07)	
	sucks	–0.056 (–0.058 to –0.053)	
	disgusting	–0.048 (–0.053 to –0.043)	
	scandals	–0.047 (–0.051 to –0.043)	
	horrible	–0.043 (–0.045 to –0.041)	
	iful	–0.042 (–0.054 to –0.031)	
	frustrating	–0.040 (–0.042 to –0.038)	
	suck	–0.040 (–0.044 to –0.036)	
	unacceptable	–0.040 (–0.043 to –0.036)	
	terrible	–0.039 (–0.041 to –0.038)	
	suicide	–0.039 (–0.04 to –0.038)	
	clar	–0.038 (–0.041 to –0.036)	
	disappointed	–0.038 (–0.041 to –0.035)	
	sco	–0.038 (–0.043 to –0.033)	
**Positive features for neutral sentiment**			
	smithsonian	0.100 (0.095 to 0.105)	
	ashton	0.094 (0.089 to 0.099)	
	spotify	0.085 (0.084 to 0.086)	
	venture	0.080 (0.073 to 0.087)	
	insp	0.077 (0.074 to 0.079)	
	antioxidants	0.075 (0.067 to 0.083)	
	beetles	0.071 (0.07 to 0.071)	
	safely	0.070 (0.07 to 0.071)	
	praise	0.066 (0.061 to 0.07)	
	announces	0.064 (0.061 to 0.067)	
	embracing	0.063 (0.062 to 0.064)	
	playlist	0.063 (0.06 to 0.066)	
	winston	0.062 (0.061 to 0.062)	
	listener	0.061 (0.061 to 0.062)	
	churchill	0.060 (0.059 to 0.06)	
**Negative features for neutral sentiment**			
	disgusting	–0.378 (–0.395 to –0.361)	
	excited	–0.344 (–0.357 to –0.33)	
	sucks	–0.334 (–0.341 to –0.327)	
	shameful	–0.322 (–0.329 to –0.314)	
	unacceptable	–0.313 (–0.331 to –0.294)	
	horrible	–0.311 (–0.317 to –0.304)	
	embarrassing	–0.307 (–0.328 to –0.287)	
	disgrace	–0.305 (–0.323 to –0.288)	
	exciting	–0.301 (–0.314 to –0.287)	
	bullshit	–0.286 (–0.296 to –0.277)	
	terrible	–0.286 (–0.291 to –0.28)	
	unbelievable	–0.280 (–0.297 to –0.264)	
	ridiculous	–0.275 (–0.282 to –0.269)	
	disappointed	–0.271 (–0.284 to –0.258)	
	unbearable	–0.268 (–0.286 to –0.25)	
**Positive features for negative sentiment**			
	disgusting	0.426 (0.407 to 0.445)	
	sucks	0.389 (0.381 to 0.397)	
	horrible	0.354 (0.347 to 0.361)	
	shameful	0.354 (0.346 to 0.362)	
	unacceptable	0.352 (0.333 to 0.371)	
	disgrace	0.343 (0.324 to 0.361)	
	embarrassing	0.342 (0.32 to 0.364)	
	terrible	0.325 (0.319 to 0.331)	
	bullshit	0.320 (0.31 to 0.331)	
	unbelievable	0.314 (0.296 to 0.331)	
	disappointed	0.309 (0.295 to 0.323)	
	ridiculous	0.306 (0.299 to 0.314)	
	unbearable	0.301 (0.281 to 0.321)	
	suck	0.292 (0.274 to 0.31)	
	stupid	0.291 (0.283 to 0.299)	
**Negative features for negative sentiment**			
	liked	–0.184 (–0.189 to –0.179)	
	blessed	–0.146 (–0.161 to –0.13)	
	enjoyed	–0.142 (–0.155 to –0.13)	
	excited	–0.136 (–0.141 to –0.132)	
	appreciate	–0.136 (–0.14 to –0.133)	
	exciting	–0.131 (–0.136 to –0.126)	
	bless	–0.121 (–0.126 to –0.115)	
	enjoying	–0.117 (–0.129 to –0.105)	
	appreciated	–0.115 (–0.12 to –0.111)	
	insp	–0.112 (–0.116 to –0.108)	
	thankful	–0.109 (–0.115 to –0.102)	
	delighted	–0.107 (–0.114 to –0.101)	
	proud	–0.106 (–0.11 to –0.102)	
	lovely	–0.106 (–0.11 to –0.102)	
	excellent	–0.104 (–0.106 to –0.101)	

### Topic Modeling

The 6 modeled topics across all tweets are presented in Table 3. We categorized topic A as *treatment* based on the frequency of terms, such as “study,” “patient,” “drug,” and “treatment.” Similarly, topic D related to *research advocacy* due to the appearance of terms such as “research,” “aid,” “funding,” “NIH” (which refers to National Institutes of Health), and “CDC” (which refers to Centers for Disease Control and Prevention), the latter of which refer to public health agencies of the United States and United Kingdom that allocate research funding. In addition, the appearance of the term “pwme” refers to an acronym which stands for “people with myalgic encephalomyelitis,” a common tag used in online support circles among those with ME/CFS. Topic B suggested a focus on *symptoms and information resources* and mentions terms such as “fatigue,” “Amazon,” “Kindle,” “bestseller,” “book,” and “story.” Topics C and E referred to *fibromyalgia* and *PCC*, respectively due to the most heavily-weighted terms in each category being the names of these conditions. Finally, topic F related to *symptoms and causes*, including terms, such as “tire,” “always,” “virus,” and “disease.”

Modeled topics across individual periods are presented in Figures S19-S36 in Multimedia Appendix 1. Before the COVID-19 pandemic, the most common comorbidity term was “fibromyalgia,” whereas during and after the pandemic, “long” and “COVID” appeared frequently. After publication of the updated NICE guideline for diagnosis and management of ME/CFS, terms such as “guideline,” “exercise,” “NICE,” “GET” (which refers to graded exercise therapy), and “CBT” (which refers to cognitive behavioral therapy) appeared.

**Table 3:** Latent Dirichlet allocation topic modeling for all tweets.

Word			Weight
**Treatment**			
	study	0.02052	
	patient	0.01787	
	treatment	0.01541	
	use	0.01179	
	link	0.00987	
	fibromyalgia	0.00930	
	drug	0.00787	
	support	0.00784	
	new	0.00727	
	virus	0.00613	
	test	0.00601	
	community	0.00599	
	group	0.00599	
	found	0.00578	
	research	0.00578	
**Symptoms and resources**			
	symptom	0.02825	
	fatigue	0.02036	
	coverup	0.01799	
	epidemic	0.01510	
	amazon	0.01425	
	kindle	0.01412	
	chronic	0.01391	
	aid	0.01251	
	treatment	0.01121	
	disease	0.01118	
	bestseller	0.01079	
	book	0.00786	
	via	0.00783	
	gut	0.00780	
	story	0.00771	
**Fibromyalgia**			
	fibromyalgia	0.03497	
	fatigue	0.02597	
	chronic	0.02349	
	health	0.01455	
	fm	0.01441	
	syndrome	0.01419	
	help	0.01180	
	report	0.01157	
	free	0.01099	
	stress	0.01094	
	exercise	0.01026	
	may	0.01022	
	recovery	0.00954	
	therapy	0.00935	
	via	0.00909	
**Research**			
	research	0.03034	
	pwme	0.01996	
	new	0.01762	
	disease	0.01148	
	5	0.01090	
	myalgice	0.01002	
	step	0.00940	
	aid	0.00815	
	nih	0.00760	
	funding	0.00721	
	cdc	0.00690	
	read	0.00613	
	book	0.00609	
	deal	0.00593	
	dr	0.00588	
**Post–COVID-19 Condition**			
	covid	0.01770	
	long	0.01667	
	people	0.01476	
	get	0.01284	
	like	0.01103	
	year	0.00882	
	say	0.00817	
	patient	0.00800	
	would	0.00794	
	know	0.00708	
	many	0.00682	
	make	0.00679	
	thing	0.00653	
	think	0.00651	
	one	0.00639	
**Symptoms and causes**			
	may	0.02335	
	tire	0.01935	
	always	0.01785	
	know	0.01399	
	news	0.01352	
	get	0.01011	
	people	0.00925	
	cause	0.00910	
	bubblews	0.00851	
	learn	0.00834	
	virus	0.00751	
	help	0.00735	
	million	0.00726	
	day	0.00691	
	disease	0.00607	

### Twitter Thematic Analysis

#### Overview

The following five themes were identified for further thematic analysis: (1) fibromyalgia, (2) treatment, (3) research, (4) UK NICE guidelines for diagnosis and management of ME/CFS, and (5) PCC. We retrieved 2.59% (23,477/906,404) tweets that mentioned fibromyalgia; 1.78% (16,112/906,404) tweets about treatment; 4.18% (37,915/906,404) tweets about research; 0.3% (2234/906,404) tweets that discussed the UK NICE guideline for diagnosis and management of ME/CFS; and 8.9% (80,685/906,404) tweets pertaining to PCC. Notably, tweets could contribute to multiple themes.

#### Theme 1: Fibromyalgia

Fibromyalgia was a dominant topic that appeared in the LDA topic modeling, and our review of representative tweets identified 6 subthemes (Table S1 in Multimedia Appendix 1). The first 2 subthemes centered on patients’ frustration with categorization of ME/CFS and fibromyalgia as psychological or psychosomatic illnesses and disappointment with the care they received. Many tweets under these themes involved negative personal experiences with friends, family, or health care providers, including dismissal of ME/CFS and fibromyalgia-related symptoms:

I can’t even count how many conversations I overheard between my parents during the 90s about how chronic fatigue syndrome and fibromyalgia were fake diseases for depressed, lazy, overweight, listless women with no other conflicts in their lives.

Doctors are so afraid of patients who have symptoms of FMG (fibromyalgia) or CFS (chronic fatigue syndrome) because they have no clue what are causes or how to treat. I now believe years of shots may be part of the causes.

That’s one thing I’m dealing with. One doctor said fibromyalgia. Another said fibromyalgia and chronic fatigue syndrome. Others look at me like I’m crazy. All I know is, I hurt all the time and I’m always tired. I’m just sick of them not listening.

The third subtheme focused on the dissemination of research investigating ME/CFS and fibromyalgia. Some tweets simply shared links to research articles on ME/CFS and fibromyalgia. Others lamented the lack of research toward these syndromes:

I think you are all on the back foot as researchers for long Covid as the similarities to the extremely underfunded chronic fatigue syndrome and related fibromyalgia conditions are completely inadequately diagnosed and treated. You have a long, long way to go to understanding.

The fourth subtheme included tweets specifically related to individuals sharing their personal experiences with ME/CFS and fibromyalgia, whereas the fifth subtheme included tweets that highlighted online support groups or educational resources for individuals with these conditions:

Jump ahead five years, I had lost over 50 pounds, was almost completely bedridden, and was too weak to sit up, talk, or feed myself. I was diagnosed with fibromyalgia and myalgic encephalomyelitis/chronic fatigue syndrome, two illnesses that we still know very little about.

I got ME/CFS in the ’90s, and it turned into fibromyalgia in 2002. (Fibro is like ME/CFS with a lot more pain.) I stopped having severe pain that required opiates several years ago, but I’m still not healthy. I wouldn’t wish it on my worst enemy.

Check out FibroFlutters - an informal “patient-led” support group based in Sunderland for people with Fibromyalgia, ME/CFS, chronic illness.

The sixth subtheme focused on recommended treatments for fibromyalgia and ME/CFS symptom management. Tweets would often reference news articles supporting the claims being discussed, with many recommending unconventional treatment modalities:

Could Green Light Therapy Help Fibromyalgia and ME/CFS?

A Methylene Blue Boost? Could a Blue Dye Help with ME/CFS, Long COVID and Fibromyalgia?

#### Theme 2: Treatment

In total, 4 subthemes were identified on the topic of treatment for ME/CFS (Table S2 in Multimedia Appendix 1). The first subtheme was that most physicians are misinformed or do more harm than good. Many tweets expressed frustration with the fact that physicians typically lacked sufficient knowledge and experience to handle the complex, multifaceted symptomology associated with ME/CFS:

It’s because regular doctors have no clue what post viral fatigue or ME/CFS is. Exercise can be harmful even if organs are fine.

I just LOVE idiot doctors who think they know more about ME/CFS than I do. None of them understand PEM [postexertional malaise], and always think I’m exaggerating. I have 40 years of experience with this, and they’re all morons.

The second related subtheme centered on physicians mislabeling ME/CFS as mental illness. Some tweets relayed experiences of physicians ascribing ME/CFS symptoms to major depressive disorder rather than acknowledging the existence of the illness. Other explanations offered by physicians included anxiety disorders or simply stating that the symptoms were psychological:

I got ME/CFS after a seriously bad case of tonsillitis at 20. My doctors thought it was depression and increased my antidepressants. Surprise! That didn’t help.

I’ve had ME/CFS for over twenty years. Doctors generally don’t care to hear about illnesses they don’t know how to treat. I’ve been told I’m perfectly fine, my illness is just in my head.

I have ME/CFS myself. This is a neurological disease. Since most doctors are not familiar with it, we sufferers are often labeled as mentally ill, although the symptoms are purely physical.

The third subtheme was dismissal of patients’ concerns by physicians. One commonly cited reason for this was because of the poorly understood pathophysiology of ME/CFS. Several Twitter users relayed experiences of not being believed by health care providers:

Example number 579 why the “Golden Girls” were light years ahead of its time: the episode where Dorothy has Chronic Fatigue Syndrome. The dismissing of her legitimate medical complaints as simple aging or lack of social life by doctors who can’t find an easy diagnosis is spot on!

I developed ME/CFS 8 years ago. It has absolutely devastated my life & the real kicker is NO Duke doctors I’ve seen have a clue how to treat/support me or don’t believe in it at all. Being disabled is extremely hard but to be gaslit & dismissed makes it so much worse.

The fourth subtheme focused on the need for patient self-advocacy to find “good” physicians or to receive appropriate treatment. Within this theme, some Twitter users advocated withholding their diagnosis of ME/CFS from their physicians to receive adequate care:

Most doctors are not your friend if you have ME/CFS. Don’t see them for that. See them for symptoms, never mention ME/CFS. They cannot help with that.

I’m super relieved (in disbelief) that after seeing a dozen+ doctors/specialists, I’ve found one who’s actually assessing my ME/CFS and POTS! Like looking at my symptoms, considering possible diagnoses and causes, and starting me on an initial treatment - *jaw on the floor*.

#### Theme 3: Research

In total, 3 subthemes were identified among tweets discussing research (Table S3 in Multimedia Appendix 1). The first subtheme centered on the dissemination of research, with Twitter users sharing links to articles or citing recent literature. Tweets typically focused on research supporting physical causes of ME/CFS, and medical treatments, with some suggesting evidence supporting a biomedical model of ME/CFS was vulnerable to suppression:

New research linking chronic fatigue syndrome to retrovirus is released after being held by journal.

The second subtheme focused on criticism of research, particularly studies that found support for management of ME/CFS with psychotherapy or graduated exercise. Furthermore, some Twitter users speculated that some ME/CFS researchers were involved in manipulating research output:

The #PACE study claimed that graded exercise training (GET) and cognitive behavioral therapy (CBT) were effective in treating Myalgic Encephalomyelitis. There were so many things wrong with this study that it’s being used as an example of how NOT to do research.

That man I have permanently blocked. He claims to be an ME/CFS researcher. Almost all of them are corrupt and get paid to keep ME/CFS patients from finding out they likely are vaccine injured or sick as a result of a post-infectious gain of function virus.

The third subtheme involved calls for increased funding for ME/CFS research. Some Twitter users compared the relatively small amounts of funding allocated to ME/CFS versus other conditions to underscore the need for increased funding:

We need massive research programmes into both long Covid and ME/CFS, coupled with better information for doctors. But above all, we need something that currently seems a long way off. A government that gives a damn.

ME/CFS research is severely underfunded. Current NIH Funding: ME/CFS has $13 million in active NIH awards. HIV/AIDS has $3294 million in active NIH awards.

#### Theme 4: UK NICE Guidelines for Diagnosis and Management of ME/CFS

We identified 6 subthemes that discussed different aspects of the UK NICE guidelines for ME/CFS (Table S4 in Multimedia Appendix 1). The first subtheme focused on perceived problems with research used to inform the initial 2007 NICE guidelines. Much of this criticism focused on the PACE (Pacing, graded Activity, and Cognitive behavior therapy: a randomized Evaluation) trial, which randomized 641 patients diagnosed with ME/CFS to either pacing, CBT, GET, or specialist medical care, and found that CBT and GET were more effective than pacing or specialist medical care for reducing fatigue and improving physical functioning [35]:

The PACE trial which has subsequently informed NICE guidelines on treatment for ME/CFS is a great example of completely ignoring the patient voice, as well as dubious research ethics (yet is still up on the Lancet).

The problem is the broad definition of ME/CFS in PACE. It makes results fairly meaningless. NICE guidelines are the problem. They treat PACE as more powerful evidence than it is. Let’s fight to change NICE in light of new evidence. A warning for GET.

The second subtheme focused on comments suggesting that changes to the UK NICE guidelines were the result of prolonged efforts by the ME/CFS community. Specifically, how the guideline was changed to revise recommendations in favor of GET and CBT after pressure from patient advocacy groups:

Many thanks to everyone who signed this petition below re removal of GET & CBT from NICE Guidelines. We’ve reached 10,000, let’s keep it going and get to 15,000.

The updated NICE guidelines for ME/CFS are out. GET is gone (which is an absolute triumph) and CBT is now a coping strategy not a treatment. I’ll take that as a win.

We worked with ForwardME to ensure this guideline was led by #pwme.

The new NICE guidelines on ME/CFS that recommend patients stay active within their safe limits was generally welcomed by patients and a petition in support of its publication received 23,000 signatures.

The third subtheme comprised tweets indicating that the 2021 UK NICE guidelines now recognized ME/CFS as a biological disease. There were several references to the 2007 NICE guideline that was perceived as supporting ME/CFS as a psychological condition, and that the 2021 update had reversed this position:

ME/CFS is placed under the Neurological Conditions section on the NICE guideline.

The recent NICE guidelines have reversed previous statements about [ME/CFS] being psychological and now acknowledge that it is physiological.

The fourth subtheme included tweets focused on the 2021 NICE guidelines revised recommendations for GET and CBT as management options for individuals with ME/CFS:

Current NICE guidelines: The results show clearly that cognitive behavioural therapy and graded exercise therapy are unsuitable treatments or management approaches for ME/CFS.

The 2021 NICE guidelines for #MECFS have a severity box that outlines the situation.... “People with severe ME/CFS are unable to do any activity for themselves or can carry out minimal daily tasks only (such as face washing or cleaning teeth).”

The newly released NICE guidelines for ME/CFS is very firm about rejecting hysteria-based GET, CBT & The Lightening Process.

The fifth subtheme focused on the need to promote and defend the 2021 NICE ME/CFS guidelines. Several comments acknowledged that there was competing evidence regarding the role of CBT and GET and advised that promotion of the 2021 NICE guidelines was critical to ensure these interventions were not offered to patients:

The people who built their careers on disabling, torturing...even killing ME/CFS patients are extremely powerful in the UK and were not going to accept this [the new NICE guidelines] because they would have to admit their fraud. So they have decided to play dirty.

I just bloody knew it. Medical professionals are going to ignore nice guidelines and rebrand GET, CBT, to try to continue pushing us down the wrong road. Be careful everyone with #ME/CFS. WATCH OUT for anything like, improving activities, gradual activity increase, etc.

The @MEAssociation has been contacting NHS trusts that still recommend CBT and GET as treatments for #MECFS, in contradiction to the 2021 NICE guideline.

The sixth subtheme included tweets advocating that the 2021 NICE ME/CFS guideline would also benefit individuals with PCC. Specifically, many tweets discussed how the revised guidelines should be used to avoid CBT and GET for individuals with PCC:

Let’s hope the recent NICE guidelines changes on ME/CFS protect those with Long Covid from going through the hell that people with ME/CFS have gone through in terms of gaslighting and medical abuse.

I am sure the CBT/GET services and researchers are worried about losing ME/CFS patients with the review of the NICE guidelines on ME/CFS. They will look to the #longcovid patients to mitigate the loss.

#### Theme 5: PCC

The fifth theme that emerged was the association between ME/CFS and PCC, within which 6 subthemes were identified (Table S5 in Multimedia Appendix 1).

The first subtheme included tweets that focused on a common biological cause between PCC and ME/CFS, although hypotheses were highly variable ranging from immunopathology, micro clots, brain stem malfunction, chronic inflammation, respiratory muscle dysfunction, and mitochondrial aberrations, among others. Relatedly, the second subtheme included tweets that focused on the erroneous attribution of PCC and ME/CFS symptoms to psychological causes:

The inner lining of blood vessels (the endothelium) plays a crucial role in maintaining cardiovascular health. A recent study found that individuals with ME/CFS and those with long COVID had significantly impaired endothelial function.

QUACK psychiatrists who have been harming patients for decades. Stop this, just stop it. Haven’t you harmed enough myalgic encephalomyelitis patients? Do you need to damage more people who have Long Covid with the same garbage psychobabble?

In terms of Long Covid and ME/CFS, there’s definitely a concerted effort on the part of some powerful people to deny, minimise and psychologise. Which allows people to believe that recovery is possible, if you have the right attitude.

The third subtheme included tweets that focused on the notion that if more research had been pursued for ME/CFS, we would now have effective treatments for PCC. The fourth subtheme comprised tweets that related to beliefs that the current attention given to PCC would likely benefit patients with ME/CFS, given the substantial overlap between these conditions:

If governments around the world had allocated more than a pittance to biomedical ME/CFS research over decades, the Long COVID fallout might not have been so severe.

There’s a huge overlap between ME/CFS and Long COVID. If only people had cared decades ago to look into ME/CFS we might have had answers for Long COVID people.

I hate to say it...but long covid is kind of a gift for us with me/cfs, because it has increased funding.

The fifth subtheme referred to warnings to avoid exercise and psychotherapy for the management of PCC and ME/CFS. Several tweets relied on the 2021 NICE guidelines for support:

Unless you have ME/CFS or Long Covid, in which case exercise can take you into total incapacity with severe intractable pain lasting for decades. Minimise exertion, rest, rest, rest.

Walking and running do not treat the underlying issue with ME/CFS or Long Covid. See NICE warning about graded exercise for LC patients and draft guidelines which say exercise should not be prescribed to ME/CFS sufferers.

The sixth subtheme comprised tweets that suggested that there was no cure for PCC or ME/CFS:

If what you have is Long Covid going on to ME/CFS, stay at home and rest, take care of yourself. There is no effective medical treatment, nothing that a doctor can give you that can make you better.

I’ve been existing with ME/CFS for the past 29 years. It’s a post-viral disease I got from Epstein Barr Virus (Mono). It’s a living death. I’ve now had Long COVID since March of 2020. It’s also a post-viral ME/CFS-like disease. There’s no treatments nor cures.

## Discussion

### Principal Findings

We found Twitter to be an active forum for discussions regarding ME/CFS, with most tweets being either neutral or negative in their sentiments. Increases in both positive and negative tweets occurred after the release of the 2021 NICE guidelines for the diagnosis and management of ME/CFS and after the onset of the COVID-19 pandemic. Common themes included treatment and symptoms of ME/CFS, fibromyalgia, research, and (after the pandemic) PCC. Our thematic analysis identified acknowledgment of considerable overlap in symptoms between fibromyalgia, ME/CFS and PCC; frustration with dismissal by physicians and labeling of these conditions as psychological; and a desire for research that identified physical causes and biomedical treatments for ME/CFS. There was considerable enthusiasm for the 2021 NICE guidelines that reversed the prior recommendation in favor of GET, claims that revisions were the result of patient advocacy efforts, and claims that both ME/CFS and PCC were lifelong conditions without effective treatments or cures.

This is the first study to analyze public sentiment regarding ME/CFS on Twitter. People living with ME/CFS browse the web up to 10-fold more often than individuals living with other chronic conditions to connect with other patients and share experiences [36]. Previous studies have confirmed the overlap in symptoms between people living with fibromyalgia and those diagnosed with ME/CFS [37], with some investigators suggesting that labels assigned to patients may be largely an artifact of medical specialization [38].

Unfortunately, several studies have found that, due to a lack of objective diagnostic markers, people living with ME/CFS often perceive stigmatization and experience delegitimizing practices by physicians [5,39]. These experiences may have clinical implications, as a recent systematic review found that stigma is associated with increased pain intensity, disability, and depression among people living with chronic pain [40]. Conversely, physician empathy has been associated with improved outcomes [41,42]. People living with ME/CFS typically report low satisfaction with physician encounters [42-44], often due to the perception that physicians are inadequately trained to manage their concerns and often attribute symptoms to psychological issues [45]. These experiences are consistent with studies of physicians reporting that one-third to half of general practitioners do not accept ME/CFS as a genuine clinical entity and, among those who do, many lack confidence in diagnosis and management [46].

ME/CFS research is a contentious topic as, although 4 decades of study since case definitions of ME and CFS were published in the mid-to-late 1980s [47], a biomedical cause or biomarkers of disease remain elusive. Prospective studies that control for ascertainment bias have failed to show an association between acute infection and development of ME/CFS [48]. In contrast, several studies have found associations between the onset of ME/CFS and elevated premorbid stress, psychopathology, severe life events, or difficulties [49,50]. These findings should not be construed as evidence that symptoms associated with ME/CFS are not real, but as support that the central nervous systems’ response to biological, psychological, and social factors may be more likely to explain symptoms versus a specific disease process [51]. However, our findings suggest this paradigm may be unacceptable to many people living with ME/CFS.

Similar issues appear to have complicated the acceptance of some interventional studies and the development of clinical practice guidelines. The largest randomized controlled trial to explore the management of ME/CFS is the PACE trial [35]; however, the finding that CBT and GET provided benefits for fatigue and physical function at both 1 and 2-year follow-up [52] is contentious for many patients and advocacy groups [53]. The ME Association recommended that NICE withdraw their recommendation for GET and that CBT should not be a primary intervention for ME/CFS, and a petition to retract the PACE trial received over 10,000 signatures from patients [54]. Our analysis of tweets also found several mentions of a prolonged campaign to pressure the 2007 UK NICE guidelines’ authors to remove recommendations in favor of CBT and GET, which were modified in the second version released in 2021 (Textbox 1).

**Changes to National Institute for Health and Care Excellence (NICE) guideline recommendations from 2007 to 2021.**

**2007 NICE myalgic encephalomyelitis/chronic fatigue syndrome (ME/CFS) guideline**

- “Cognitive behavioural therapy (CBT) and/or graded exercise therapy (GET) should be offered to people with mild or moderate CFS/ME and provided to those who choose these approaches, because currently these are the interventions for which there is the clearest research evidence of benefit.”

**2021 NICE ME/CFS guidelines**

- “Do not offer people with ME/CFS any program…that uses fixed incremental increases in physical activity or exercise, for example, graded exercise therapy”
- “Only offer CBT to adults, children and young people with ME/CFS if, after discussing it, they would like to use it to support them in managing their symptoms…Explain that CBT for people with ME/CFS does not assume people have ‘abnormal’ illness beliefs and behaviours as an underlying cause of their ME/CFS, but recognises that thoughts, feelings, behaviours and physiology interact with each other.”

The updated NICE guidance justified changes in recommendations regarding CBT and GET for 4 reasons. First, by downgrading the evidence for indirectness because trials that provided supporting evidence did not require patients to screen positive for postexertional malaise (PEM), even though treatment effects were similar across patients with different diagnostic criteria (including those with PEM). Second, they rejected evidence from randomized trials that found GET was not harmful in favor of anecdotal reports by patients that GET worsened their symptoms. Third, they considered outcomes at the longest follow-up reported, which for the PACE trial meant results were confounded by cross-over. Finally, the guideline panel rejected evidence from Cochrane reviews because they did not report on mortality—an outcome that Cochrane review authors did not view as relevant for trials of CBT or GET [55].

Our analysis suggests the updated NICE guideline was well-received by patients and may be associated with the increase in positive tweets at this time. However, 4 members of the 2021 NICE guideline development committee resigned in protest [56], representatives of 7 UK medical groups (including the Royal College of Physicians) signed a joint statement relaying concerns with the guideline [57], and more than 50 international specialists analyzed the guideline and concluded that “the consequences of this are that patients may be denied helpful treatments and therefore risk persistent ill health and disability” [58]. At present, there are at least 2 ongoing campaigns by ME/CFS advocacy groups to have other publications they disagree with retracted: a Cochrane review that found GET was helpful for ME/CFS [59] and a deep phenotyping study of patients with ME/CFS that found functional limitations were due to “altered effort preference” [60].

Mention of PCC in tweets made after the onset of the pandemic aligns with studies that have shown a large overlap in symptoms between this condition and ME/CFS [18]. Interest by patients with ME/CFS may also be driven by the renewed attention to postinfectious fatigue syndromes that PCC has generated and resulting new investments in research. For example, the US NIH recently committed US $1 billion to fund PCC research [61]. Patients with a diagnosis of PCC face similar issues as patients with ME/CFS. Female sex and psychological factors are risk factors for developing PCC [62,63], infection with COVID-19 may not be required to manifest symptoms of PCC [64], and CBT and graduated exercise [65] are emerging as promising therapies. A Twitter sentiment analysis of 814,951 tweets on PCC also found key topics included calls for more research and better treatments [15].

The notion that recovery from ME/CFS is not possible is inconsistent with the evidence. Although only 5% of patients experience full recovery without targeted intervention [66], observational studies have reported a recovery rate of 18% following CBT [67,68]. There are entire organizations dedicated to recovered patients, such as Recovery Norge [69]. Interviews with patients who have fully recovered from ME/CFS reveal a consistent pattern of engagement with graduated exercise and psychotherapy to increase self-agency [70,71]. Furthermore, recovery from ME/CFS is associated with not attributing illness to a physical cause and a greater sense of control over symptoms [66]. However, patients who achieve recovery report conflicts with patients who have not, including skepticism about whether they had ME/CFS. Once patients recover from ME/CFS, they are less likely to remain engaged with online support groups [72].

### Strengths and Limitations

Strengths of our study include a large dataset of almost 1 million tweets. In addition, we performed sentiment analysis with a RoBERTa model, which has previously been trained on 58 million tweets. Finally, we ensured that representative subthemes were identified by independent raters which increases confidence in the reliability of our findings.

Our study also has several limitations. The method of tweet collection relied on a list of search terms that may have missed some relevant tweets and NLP models can misinterpret the sentiment of tweets, particularly when sarcasm is used [73]. As NLP methods evolve, it may become possible to harness the power of GPT models to facilitate more accurate sentiment, emotional, and thematic analyses. Discussions surrounding PCC in the post-UK NICE publication dataset may have suffered from some confounding with the post–COVID-19 pandemic dataset due to the overlap in dates, particularly because PCC was identified relatively late into the pandemic. In addition, demographics, geographic location, and previous daily activities of the authors of text-based social media data can result in different word choices even when discussing the same subject matter [74-76], and we were unable to adjust for these factors.

### Implications

Our findings highlight several important issues. First, although current evidence supports exercise therapy [77] and CBT [78] for the management of ME/CFS, some patients find these approaches unacceptable. In part, due to concerns about harm (eg, PEM following GET) and stigmatization (with CBT). Further research is needed to inform how best to support patients’ engagement with evidence-based care. Second, patients often report unsatisfactory health care encounters leading to disengagement and a desire to attend clinicians that view ME/CFS as purely physical disorder. For example, some surgeons offer cranial and spinal decompression as a treatment for ME/CFS [79], despite a lack of evidence supporting this approach. These findings reinforce the importance of building therapeutic relationships with patients living with ME/CFS that include addressing possible concerns about mind-body treatment approaches. Patients who view their health care provider as sympathetic may be more willing to engage in shared decision-making about interventions they are considering.

Third, our findings with respect to the potential influence of advocacy efforts on science are especially critical given the increasingly recognized importance of including patient partners in research [80]. Involvement of patient partners can improve the quality and relevance of research efforts [81]; however, participants with important intellectual conflicts of interest can compromise the research process and reduce the trustworthiness of results [82]. Finally, ME/CFS is not the first postinfectious syndrome, and will not be the last [83]. The latest variant is PCC, which, at present, is the focus of considerable attention and research funding. This presents important opportunities to advance our understanding of the etiology, prognosis, and effective management of this disorder. Such efforts would be more valuable if they considered the degree to which results may be generalizable to postinfectious fatigue syndromes in general.

### Future Directions

Twitter is an important source of information and communication for people living with ME/CFS. The degree to which advice is credible and consistent with the current best evidence is therefore important [84]. Our findings suggest that some individuals living with ME/CFS who post on Twitter believe that GET is harmful, CBT is ineffective, and recovery is not possible. Efforts should be made to promote the dissemination of evidence-based information on Twitter and assist patients in assessing the credibility of statements made on social media. Removing hope of improvement or recovery from ME/CFS can have dire consequences for some patients [85-88]. A survey of members of the Canadian Association of Medical Assistance in Dying Assessors and Providers found that ME/CFS was the second most common nonfatal condition for which medical assistance in dying was requested [89].

## Appendix

Multimedia Appendix 1 Additional details on tweet retrieval and analysis.

## Abbreviations

API: application programming interface
BERT: Bidirectional Embedding Representations from Transformers
CBT: cognitive behavioral therapy
CFS: chronic fatigue syndrome
GET: graded exercise therapy
LDA: latent Dirichlet allocation
ME: myalgic encephalomyelitis
NICE: National Institute for Health and Care Excellence
NIH: National Institutes of Health
NLP: natural language processing
PACE: Pacing, graded Activity, and Cognitive behavior therapy: a randomized Evaluation PCC: post-COVID-19 condition
PEM: postexertional malaise
RoBERTa: Robustly Optimized Bidirectional Embedding Representations from Transformers Pretraining Approach
SHAP: Shapley Additive Explanations

## Data Availability

The code used to generate these analyses is available on GitHub [34]. The datasets generated during this study are available from the corresponding author on reasonable request.
