# Supplementary material for: Impact of the COVID-19 Pandemic and the 2021 National Institute for Health and Care Excellence Guidelines on Public Perspectives Toward Myalgic Encephalomyelitis/Chronic Fatigue Syndrome: Thematic and Sentiment Analysis on Twitter (Rebranded as X)
Source: J Med Internet Res. 2025 May 21;27:e65087. doi: 10.2196/65087 (PMC12138300; doi:10.2196/65087)
Supplement: Multimedia Appendix 1 [file jmir_v27i1e65087_app1.docx]

APPENDIX

[Figure S1. Pre-COVID positive features for positive sentiment 1](#_Toc198368949)

[Figure S2. Pre-COVID negative features for positive sentiment 2](#_Toc198368950)

[Figure S3. Pre-COVID positive features for neutral sentiment 3](#_Toc198368951)

[Figure S4. Pre-COVID negative features for neutral sentiment 4](#_Toc198368952)

[Figure S5. Pre-COVID positive features for negative sentiment 5](#_Toc198368953)

[Figure S6. Pre-COVID negative features for negative sentiment 6](#_Toc198368954)

[Figure S7. Post-COVID positive features for positive sentiment 7](#_Toc198368955)

[Figure S8. Post-COVID negative features for positive sentiment 8](#_Toc198368956)

[Figure S9. Post-COVID positive features for neutral sentiment 9](#_Toc198368957)

[Figure S10. Post-COVID negative features for neutral sentiment 10](#_Toc198368958)

[Figure S11. Post-COVID positive features for negative sentiment 11](#_Toc198368959)

[Figure S12. Post-COVID negative features for negative sentiment 12](#_Toc198368960)

[Figure S13. Post-UK NICE guidelines positive features for positive sentiment 13](#_Toc198368961)

[Figure S14. Post-UK NICE guidelines negative features for positive sentiment 14](#_Toc198368962)

[Figure S15. Post-UK NICE guidelines positive features for neutral sentiment 15](#_Toc198368963)

[Figure S16. Post-UK NICE guidelines negative features for neutral sentiment 16](#_Toc198368964)

[Figure S17. Post-UK NICE guidelines positive features for negative sentiment 17](#_Toc198368965)

[Figure S18. Post-UK NICE guidelines negative features for negative sentiment 18](#_Toc198368966)

[Figure S19. Pre-COVID LDA topic 1 19](#_Toc198368967)

[Figure S20. Pre-COVID LDA topic 2 20](#_Toc198368968)

[Figure S21. Pre-COVID LDA topic 3 21](#_Toc198368969)

[Figure S22. Pre-COVID LDA topic 4 22](#_Toc198368970)

[Figure S23. Pre-COVID LDA topic 5 23](#_Toc198368971)

[Figure S24. Pre-COVID LDA topic 6 24](#_Toc198368972)

[Figure S25. Post-COVID LDA topic 1 25](#_Toc198368973)

[Figure S26. Post-COVID LDA topic 2 26](#_Toc198368974)

[Figure S27. Post-COVID LDA topic 3 27](#_Toc198368975)

[Figure S28. Post-COVID LDA topic 4 28](#_Toc198368976)

[Figure S29. Post-COVID LDA topic 5 29](#_Toc198368977)

[Figure S30. Post-COVID LDA topic 6 30](#_Toc198368978)

[Figure S31. Post-UK NICE guidelines LDA topic 1 31](#_Toc198368979)

[Figure S32. Post-UK NICE guidelines LDA topic 2 32](#_Toc198368980)

[Figure S33. Post-UK NICE guidelines LDA topic 3 33](#_Toc198368981)

[Figure S34. Post-UK NICE guidelines LDA topic 4 34](#_Toc198368982)

[Figure S35. Post-UK NICE guidelines LDA topic 5 35](#_Toc198368983)

[Figure S36. Post-UK NICE guidelines LDA topic 6 36](#_Toc198368984)

[Table S1. Representative tweets for ME/CFS and fibromyalgia 37](#_Toc198368985)

[Table S2. Representative Tweets for Treatment of ME/CFS 43](#_Toc198368986)

[Table S3. Representative Tweets for Research 52](#_Toc198368987)

[Table S4. Representative Tweets for the NICE ME/CFS Guidelines 56](#_Toc198368988)

[Table S5. Representative Tweets for ME/CFS and Long COVID 66](#_Toc198368989)

#

# Figure S1. Pre-COVID positive features for positive sentiment


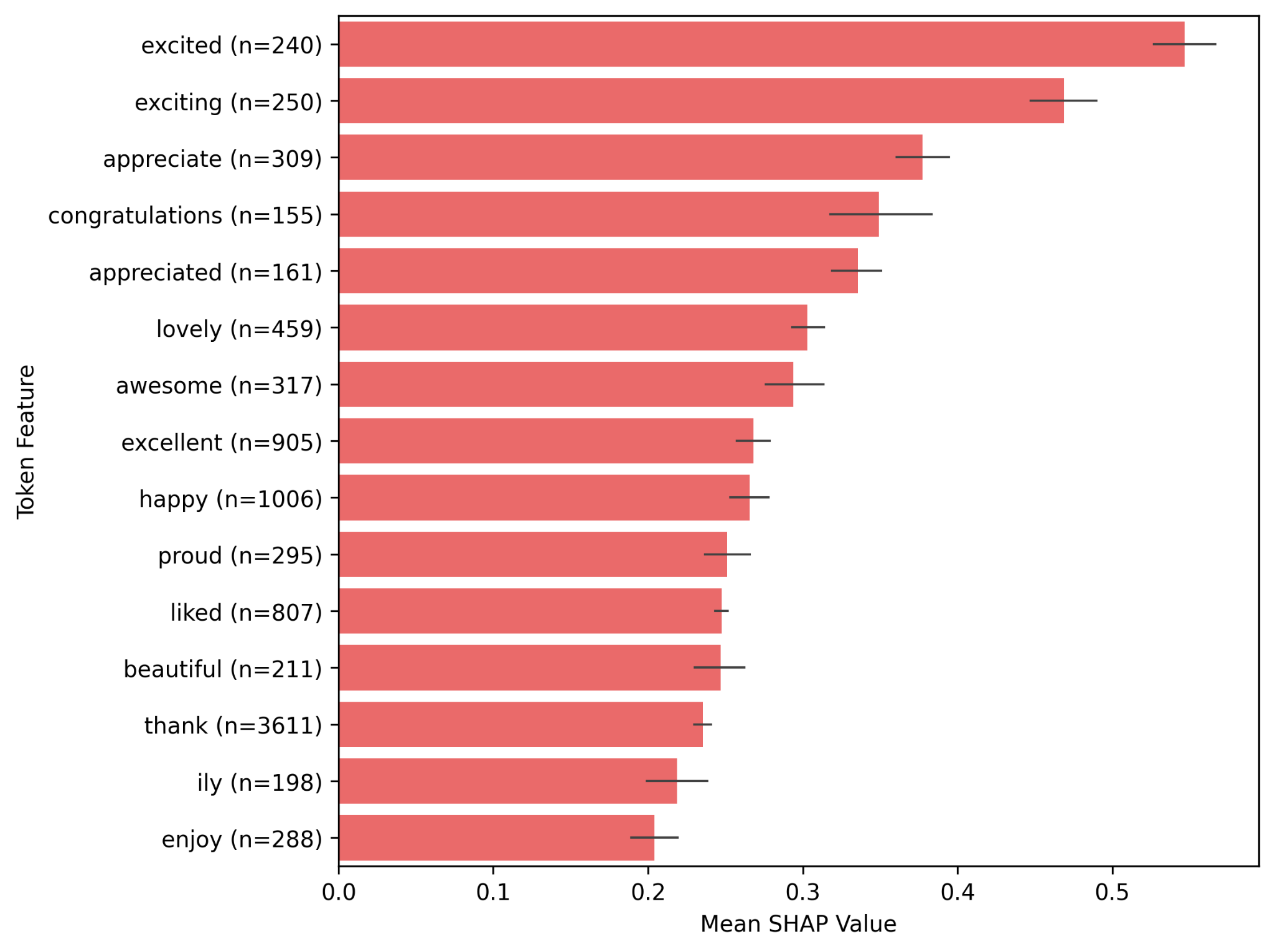


# Figure S2. Pre-COVID negative features for positive sentiment


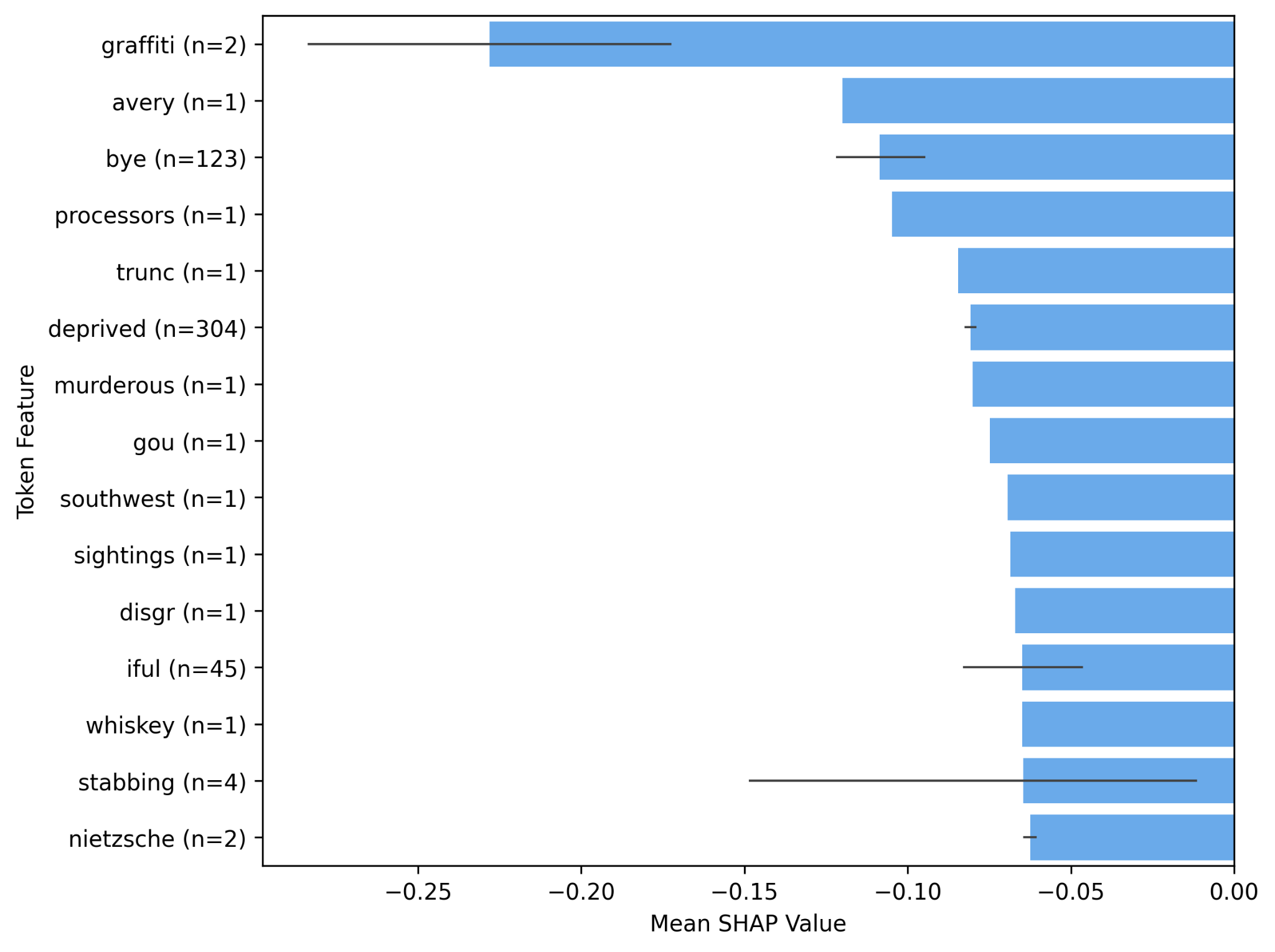


#

# Figure S3. Pre-COVID positive features for neutral sentiment


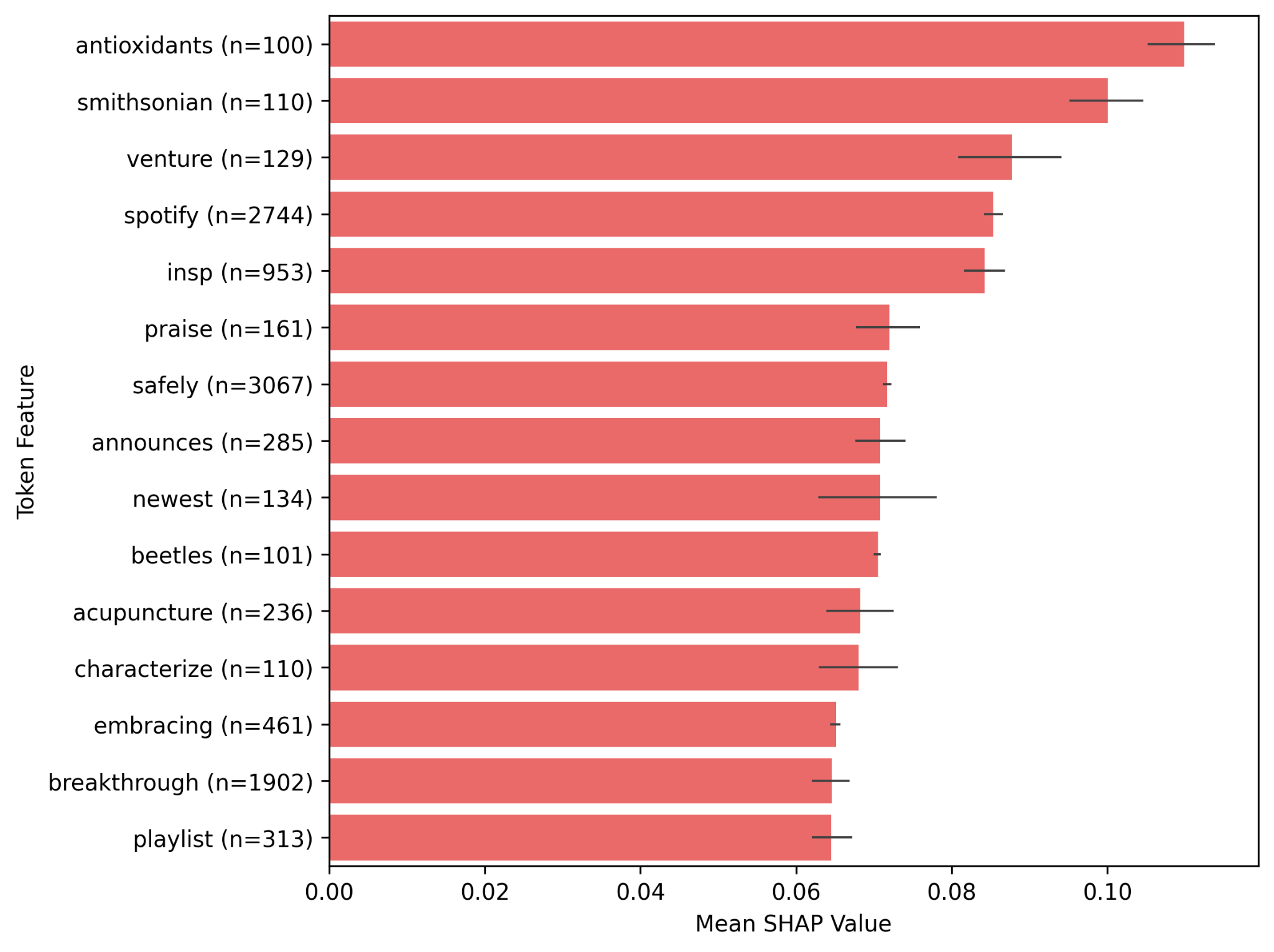


# Figure S4. Pre-COVID negative features for neutral sentiment


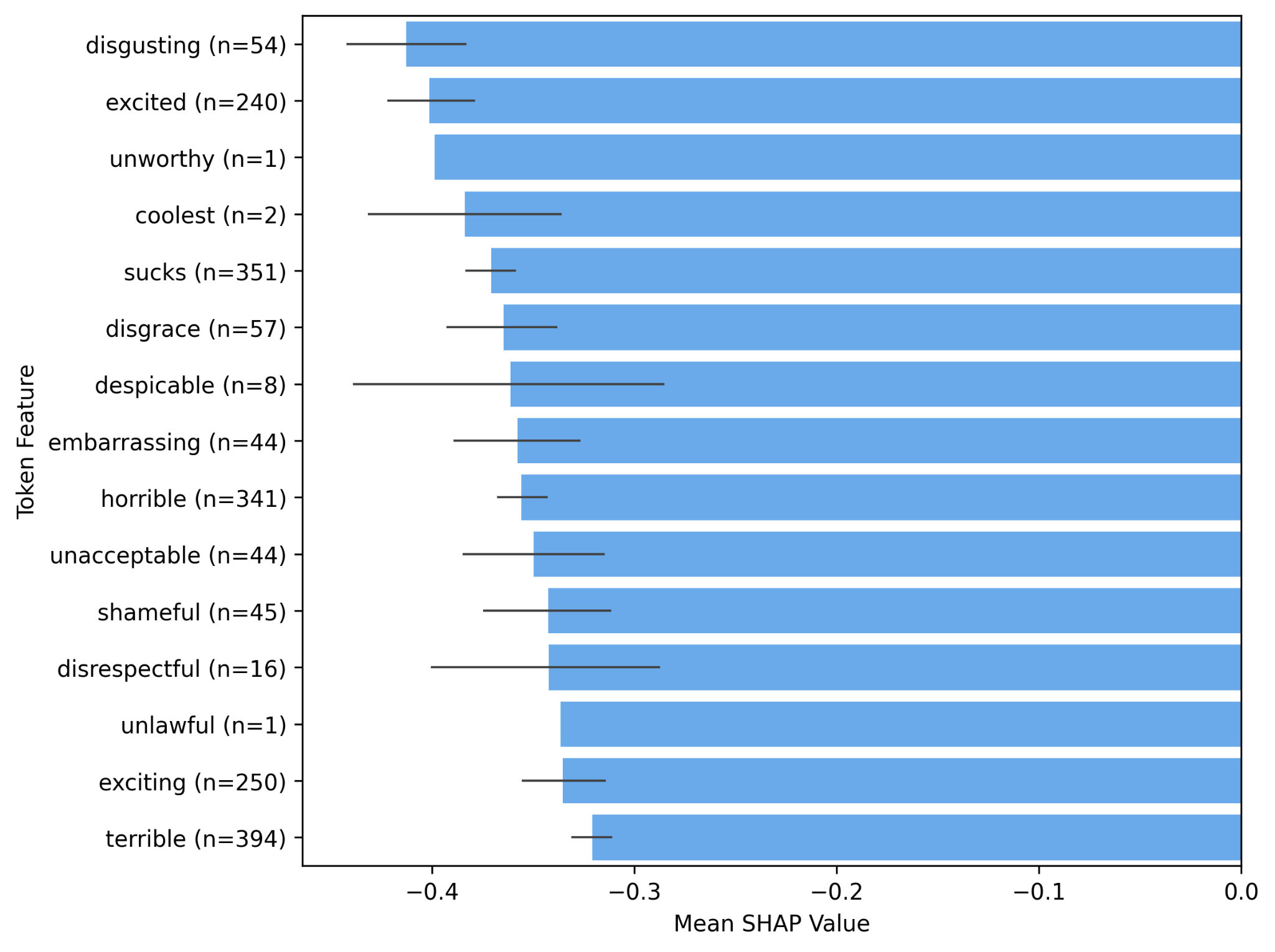


#

# Figure S5. Pre-COVID positive features for negative sentiment


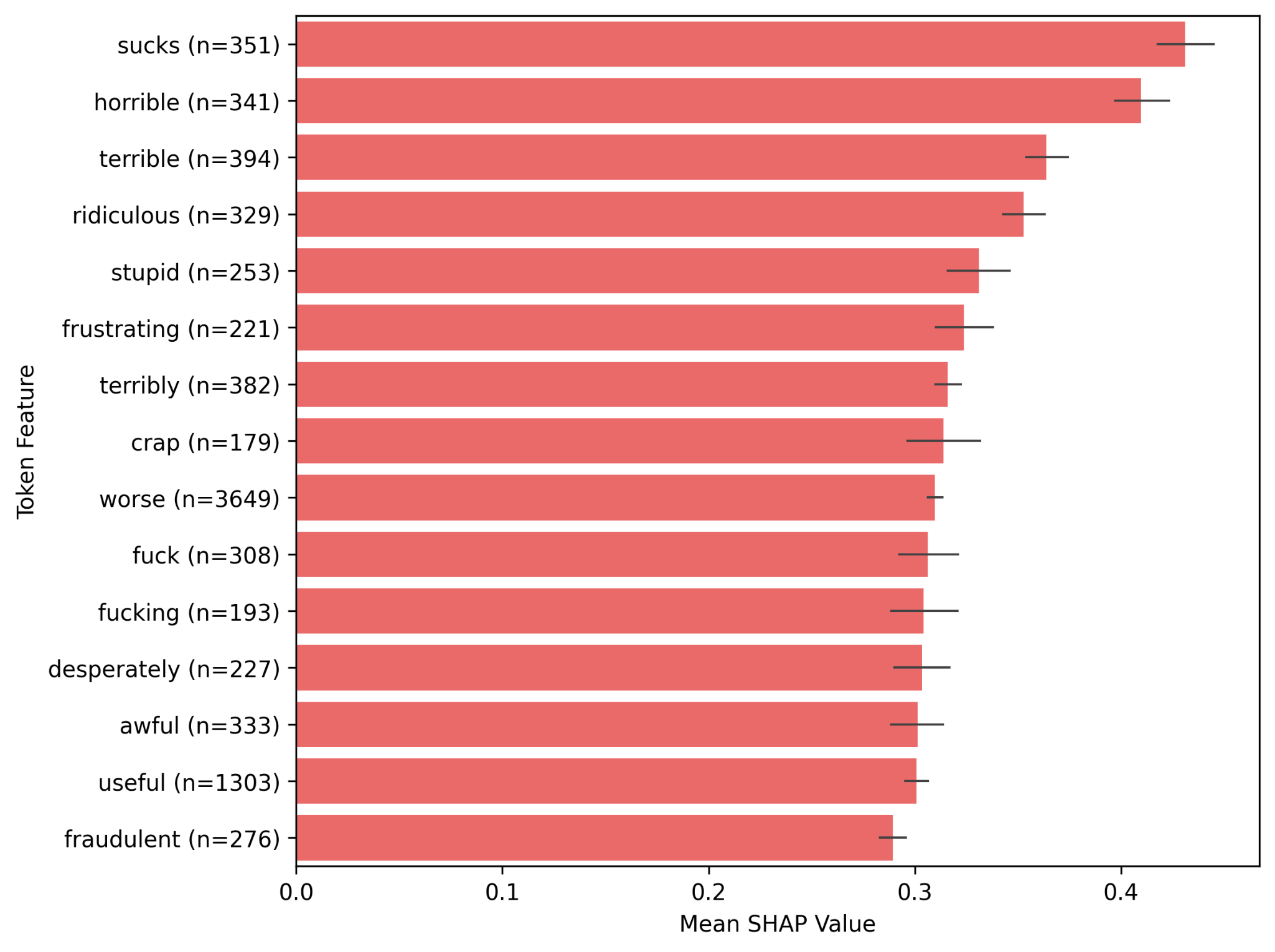


#

# Figure S6. Pre-COVID negative features for negative sentiment


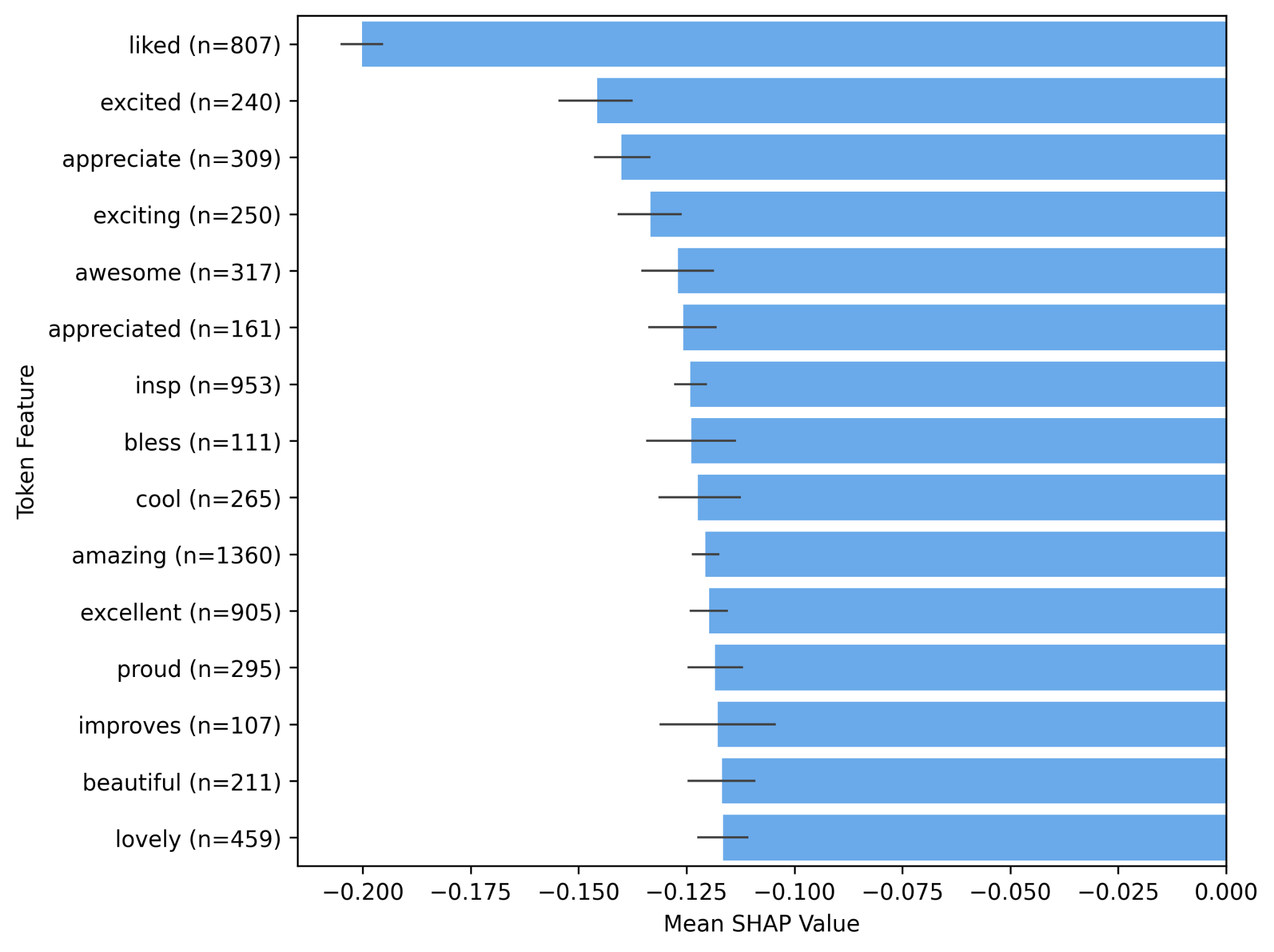


# Figure S7. Post-COVID positive features for positive sentiment


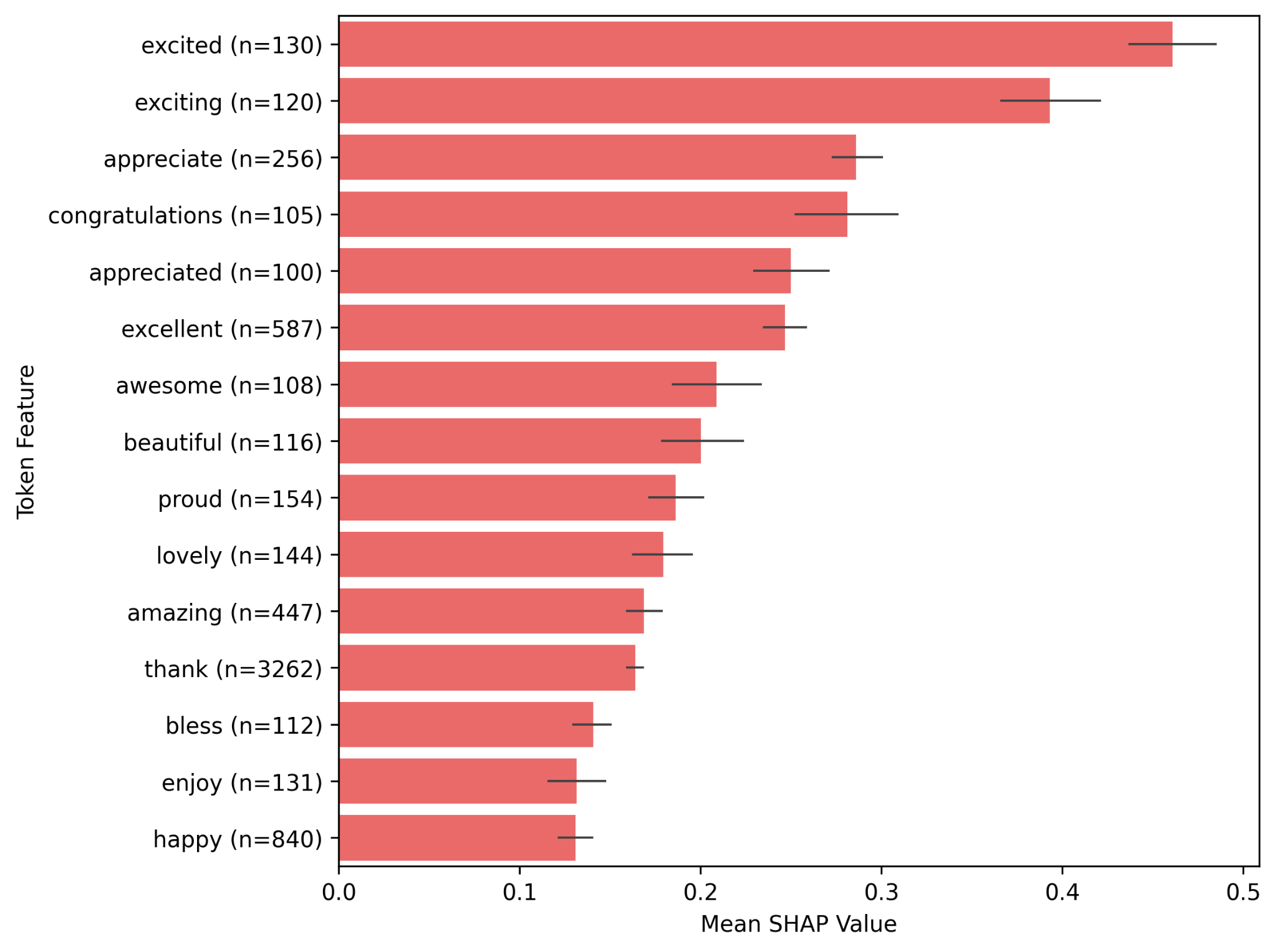


# Figure S8. Post-COVID negative features for positive sentiment


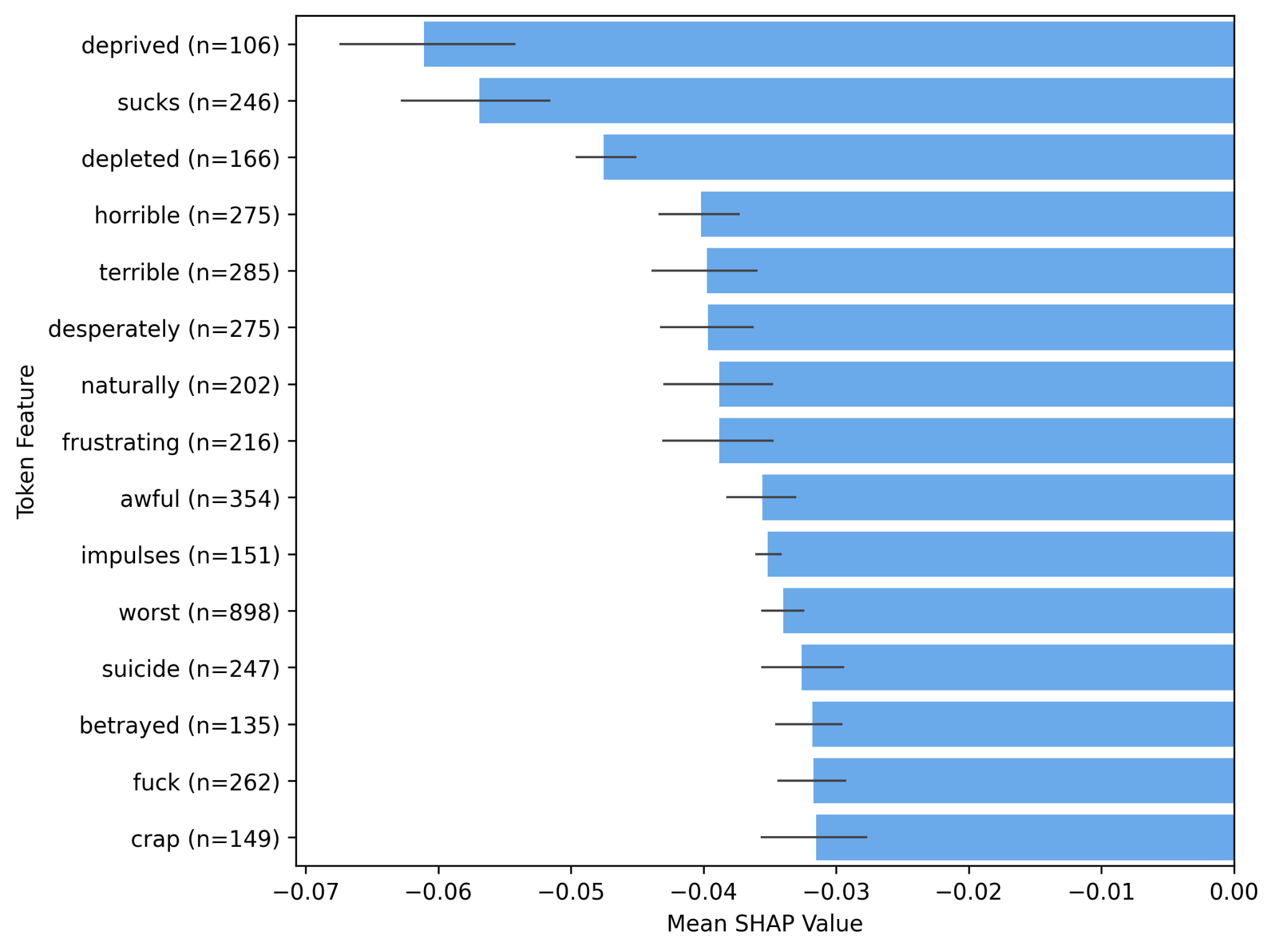


# Figure S9. Post-COVID positive features for neutral sentiment


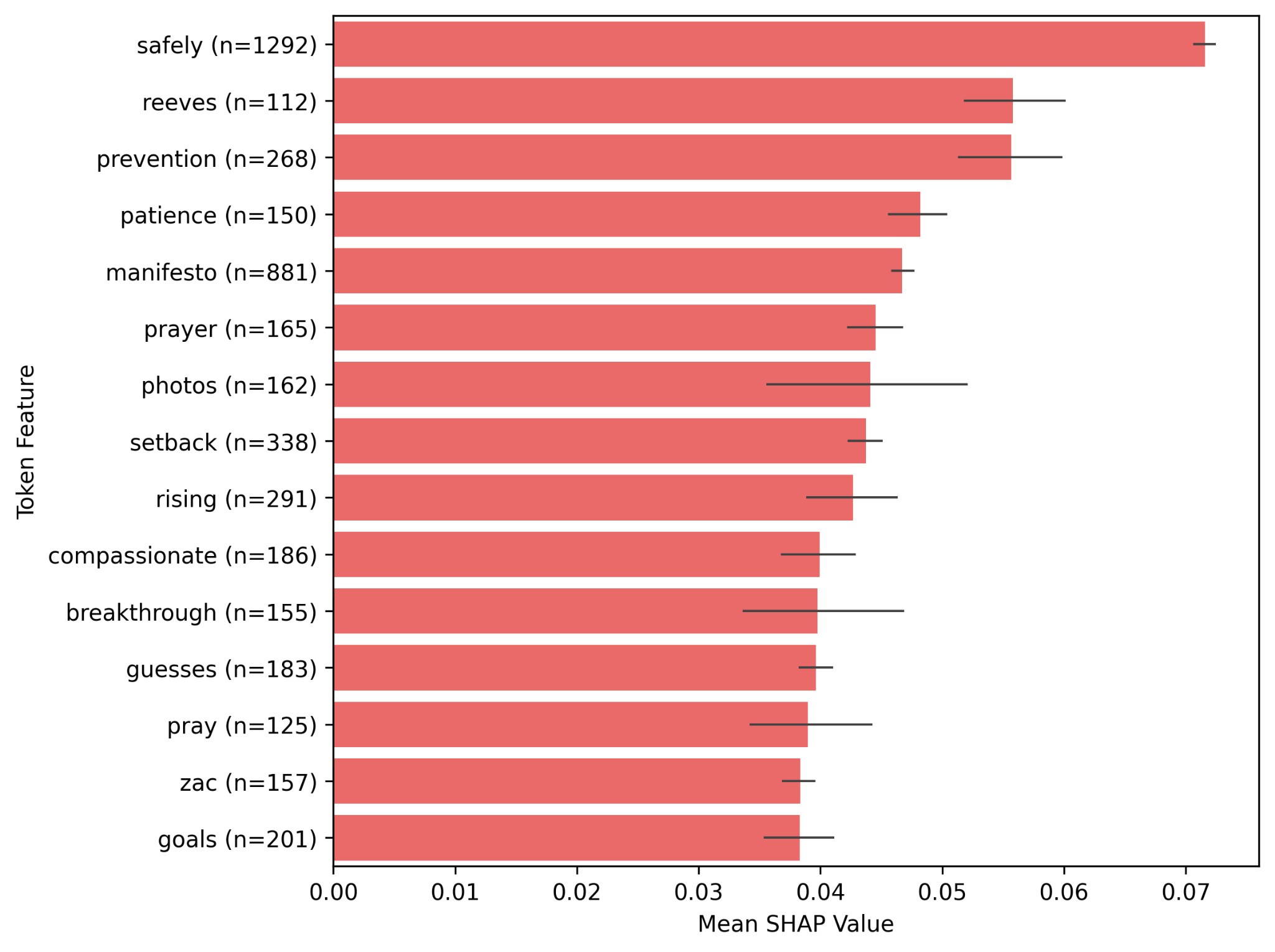


# Figure S10. Post-COVID negative features for neutral sentiment


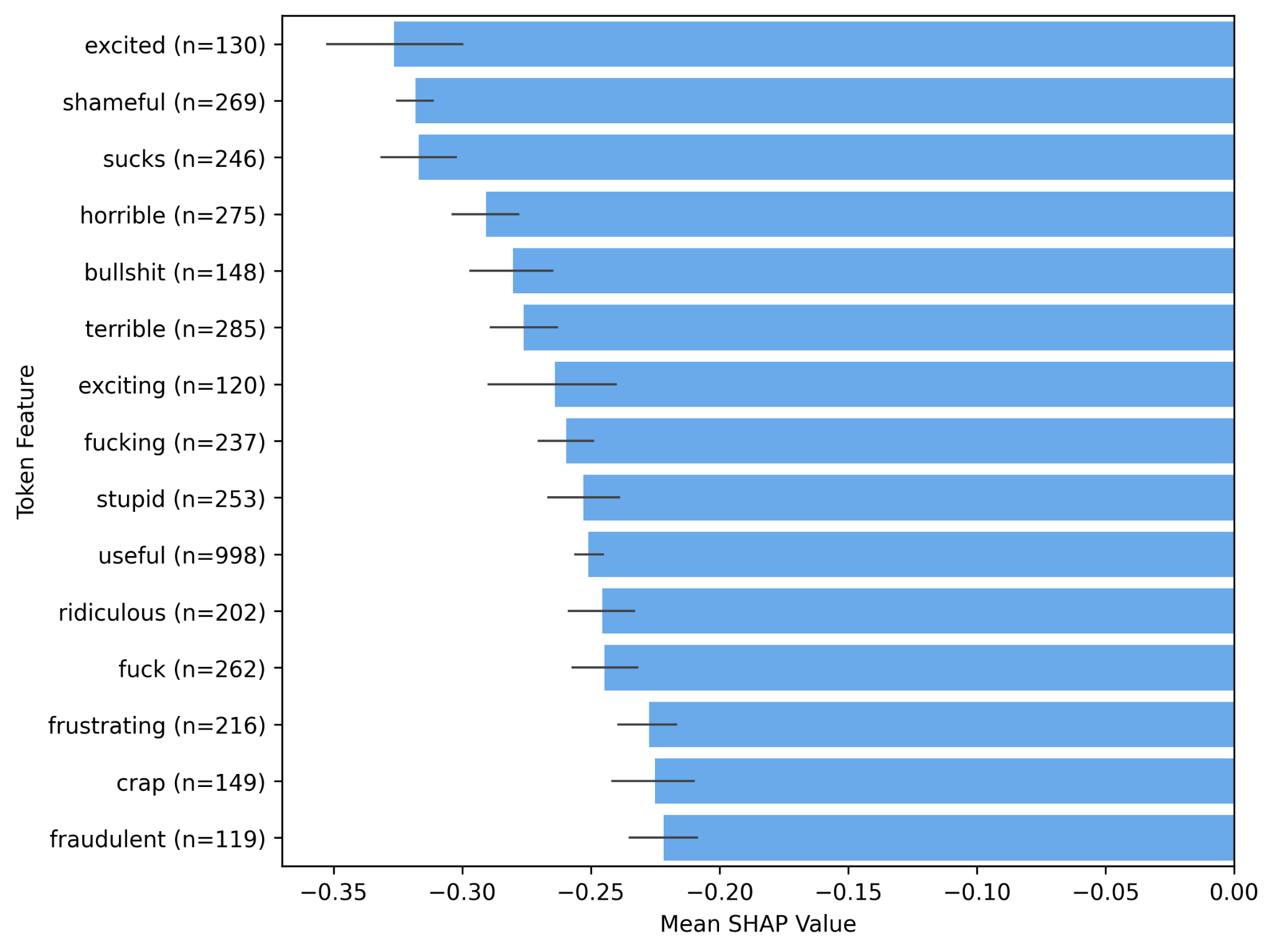


# Figure S11. Post-COVID positive features for negative sentiment


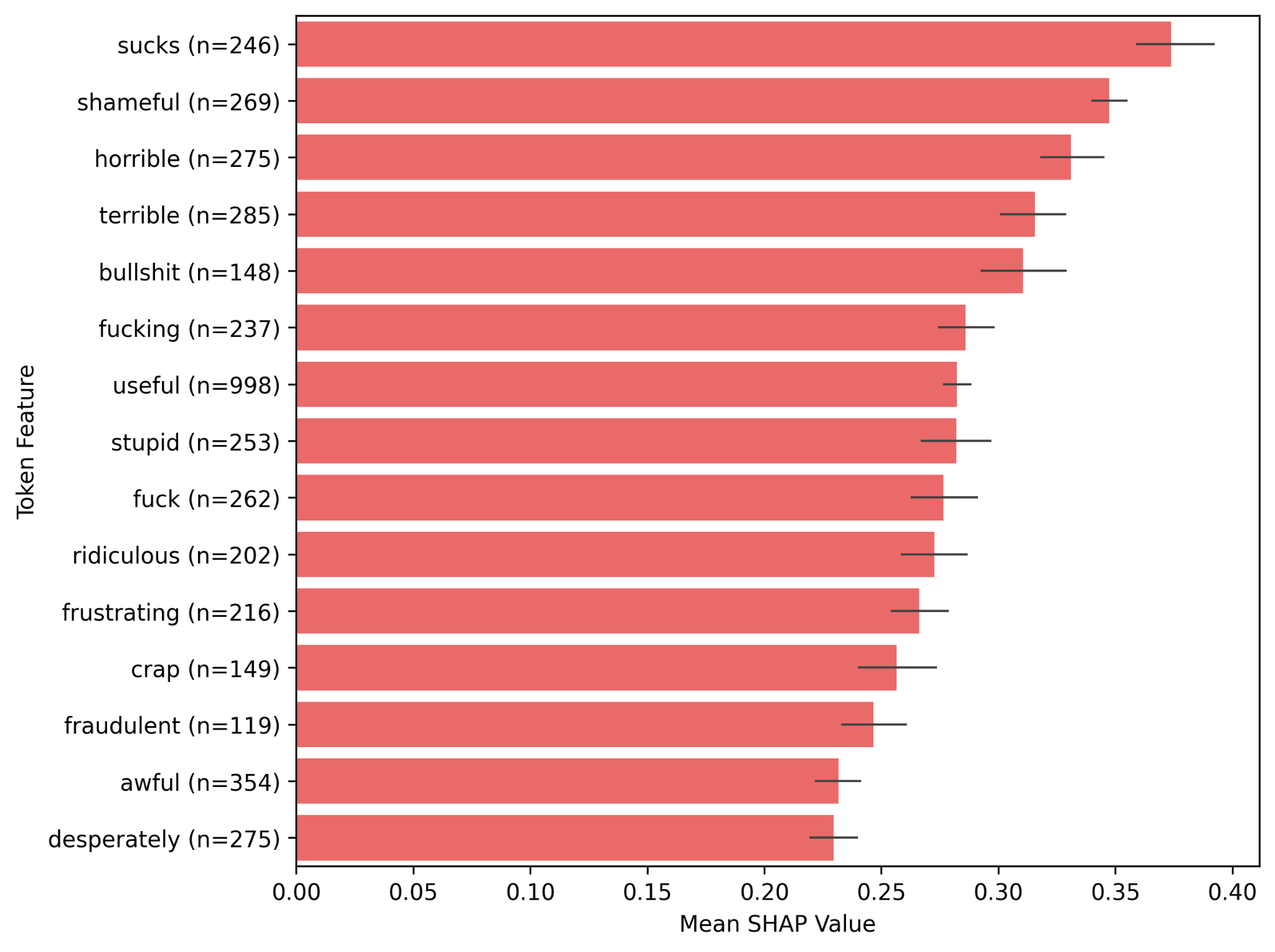


# Figure S12. Post-COVID negative features for negative sentiment


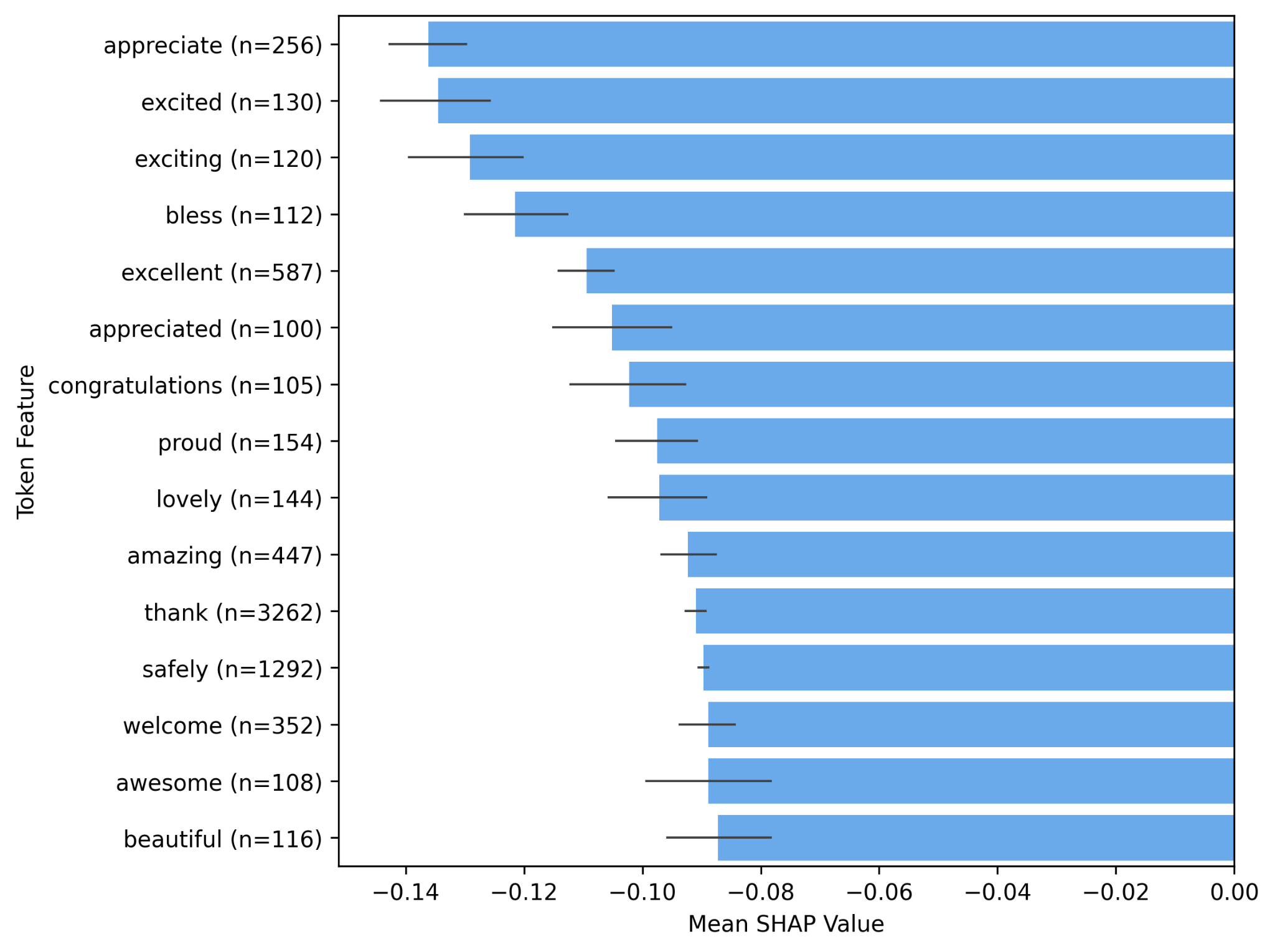


# Figure S13. Post-UK NICE guidelines positive features for positive sentiment


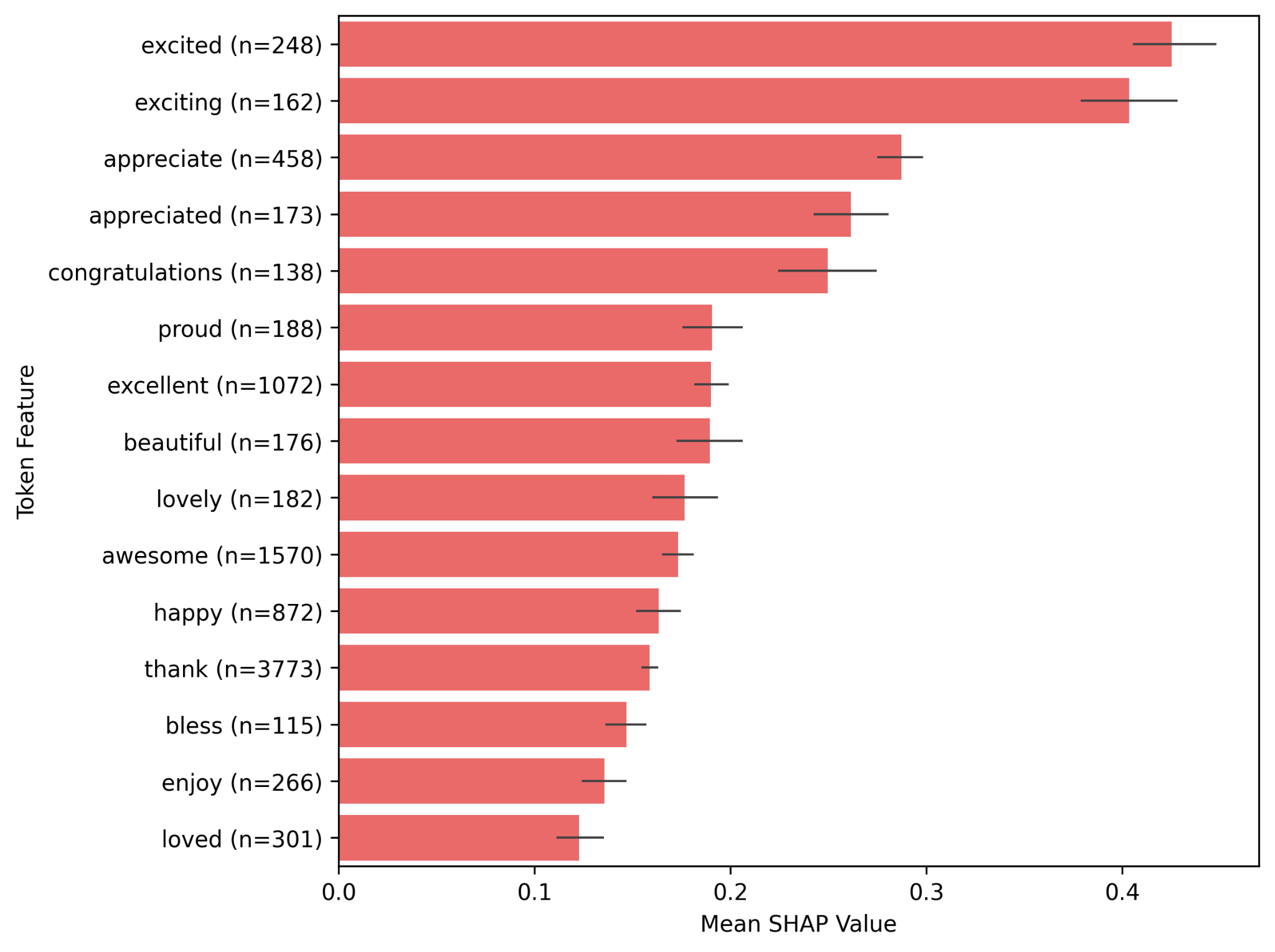


# Figure S14. Post-UK NICE guidelines negative features for positive sentiment


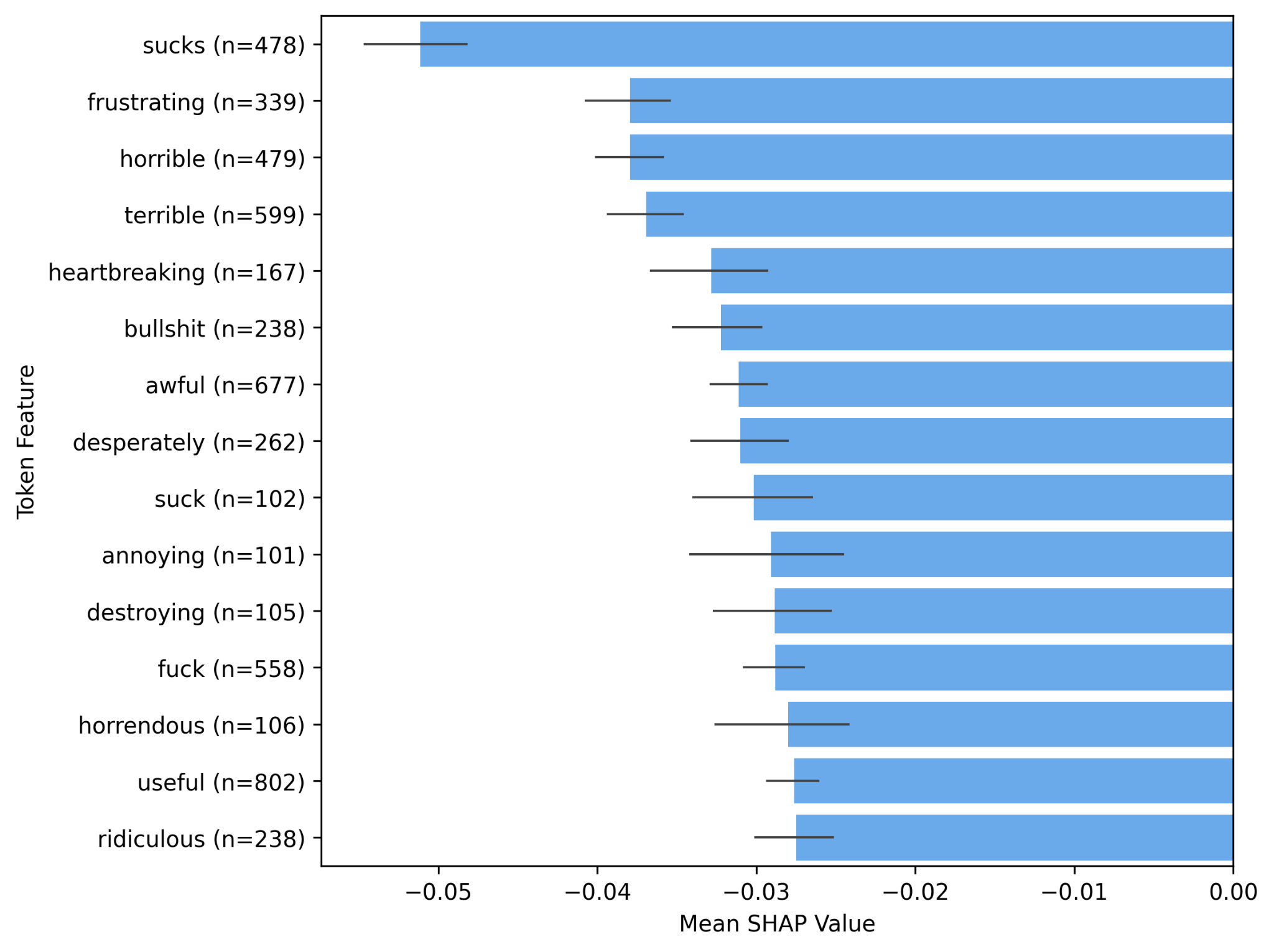


# Figure S15. Post-UK NICE guidelines positive features for neutral sentiment


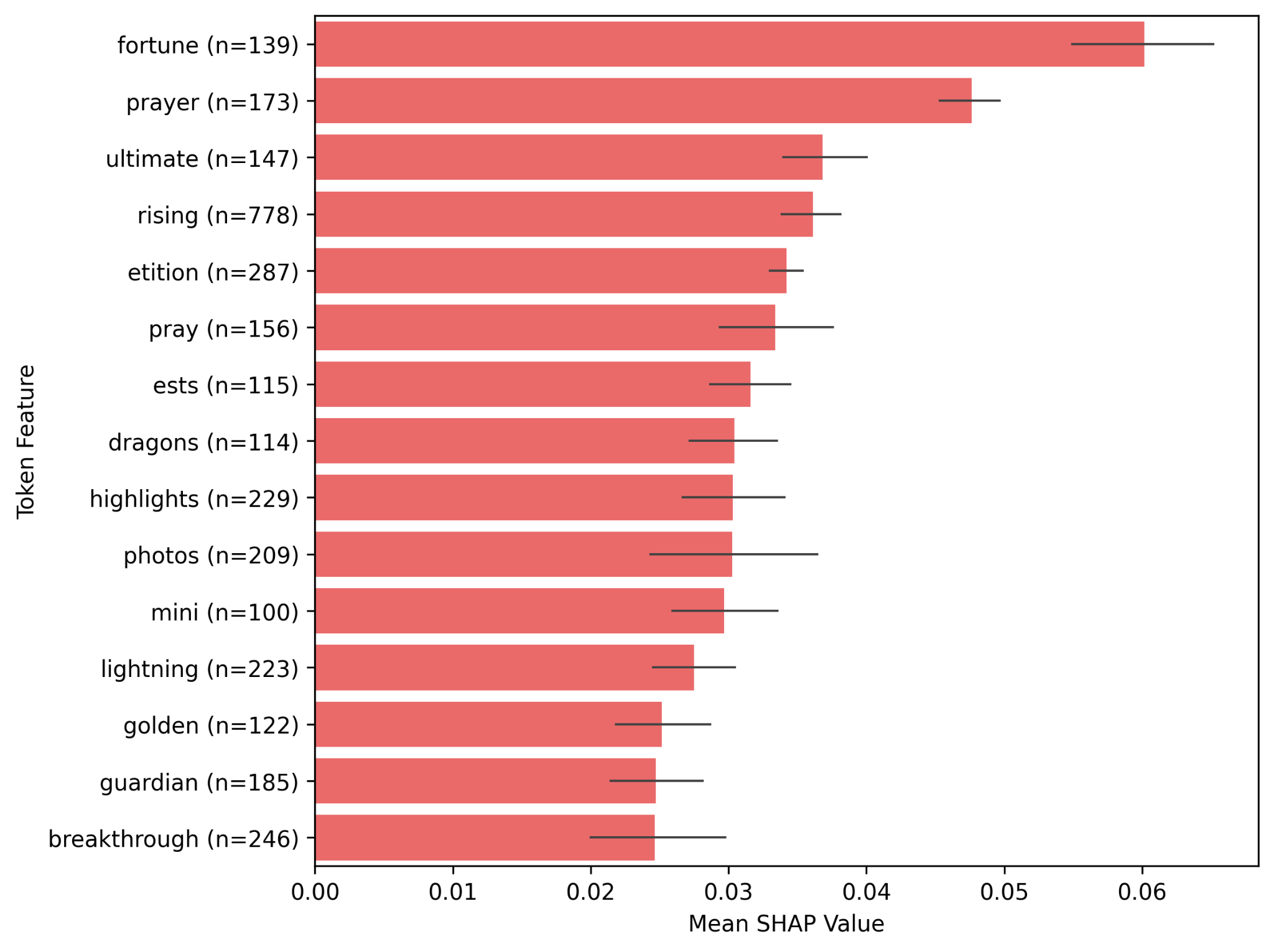


# Figure S16. Post-UK NICE guidelines negative features for neutral sentiment


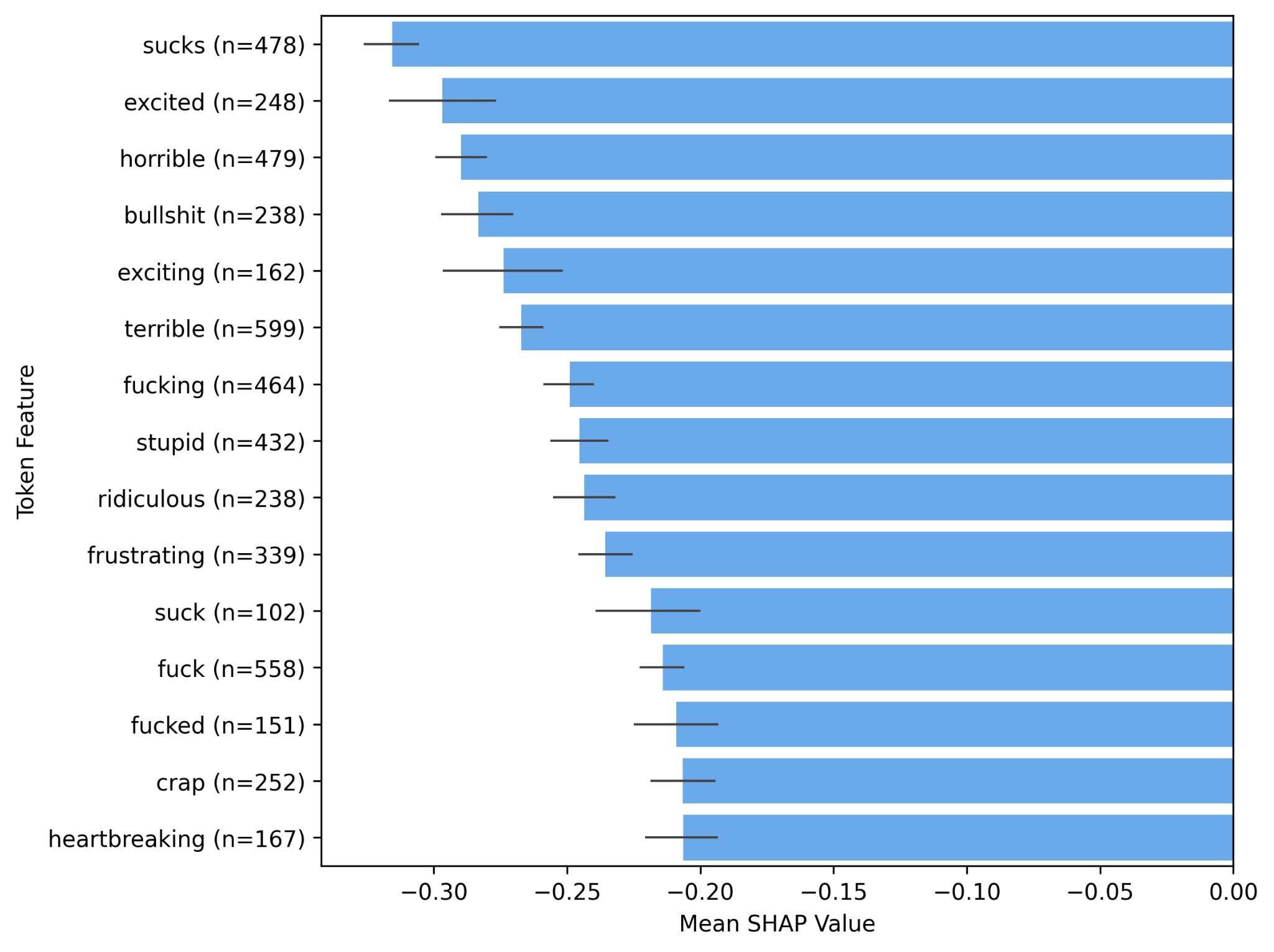


# Figure S17. Post-UK NICE guidelines positive features for negative sentiment


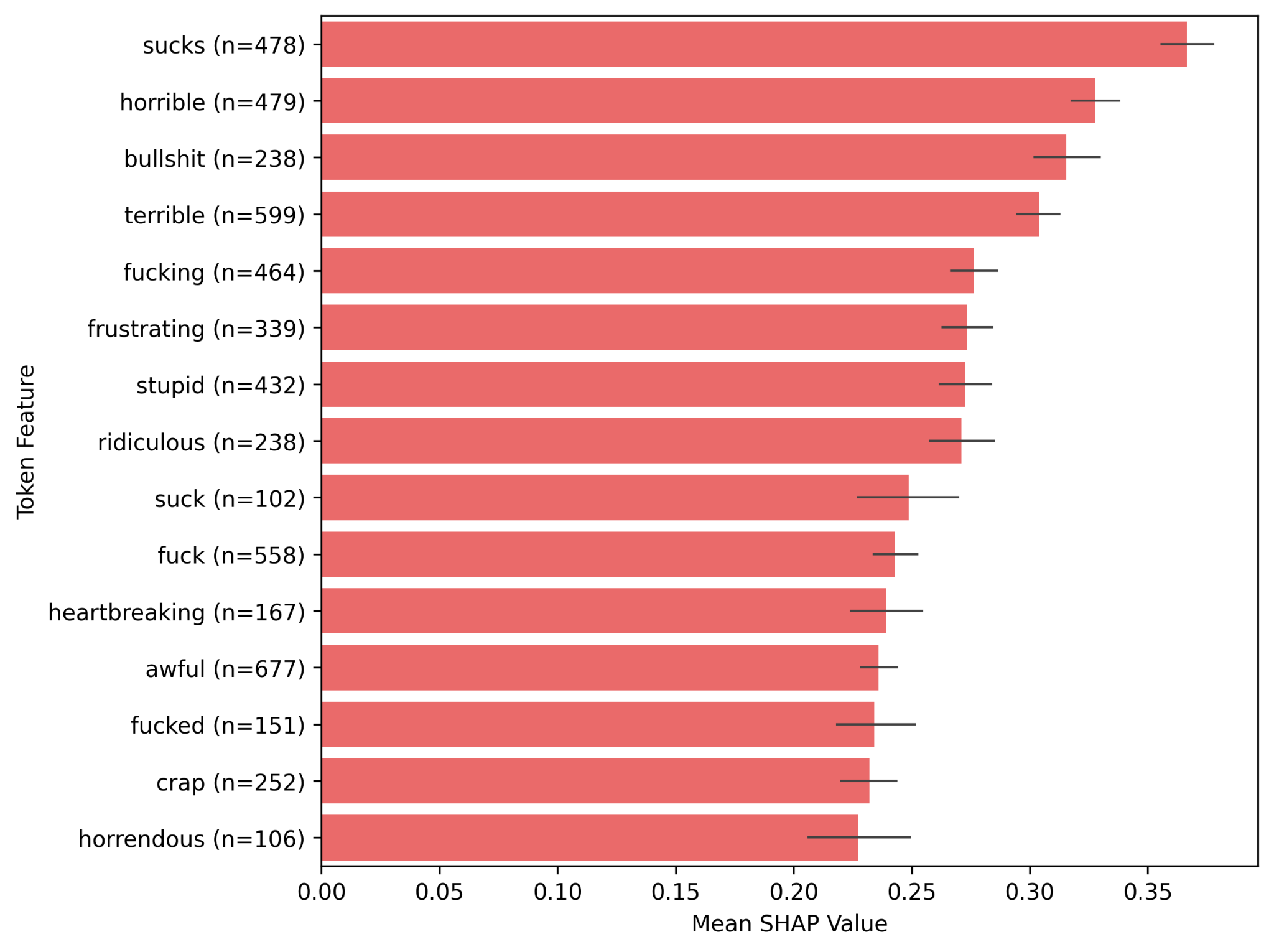


# Figure S18. Post-UK NICE guidelines negative features for negative sentiment


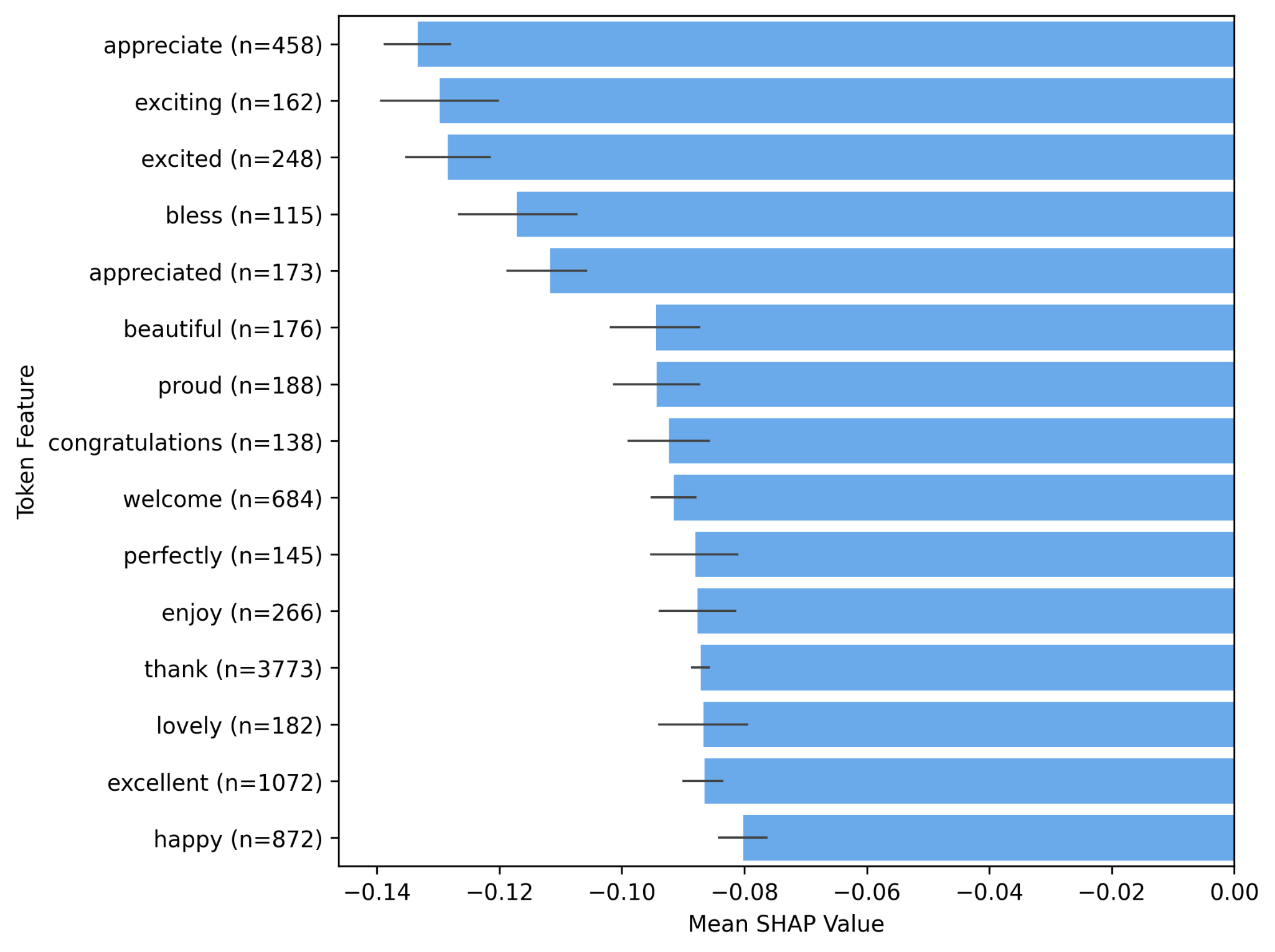


# Figure S19. Pre-COVID LDA topic 1


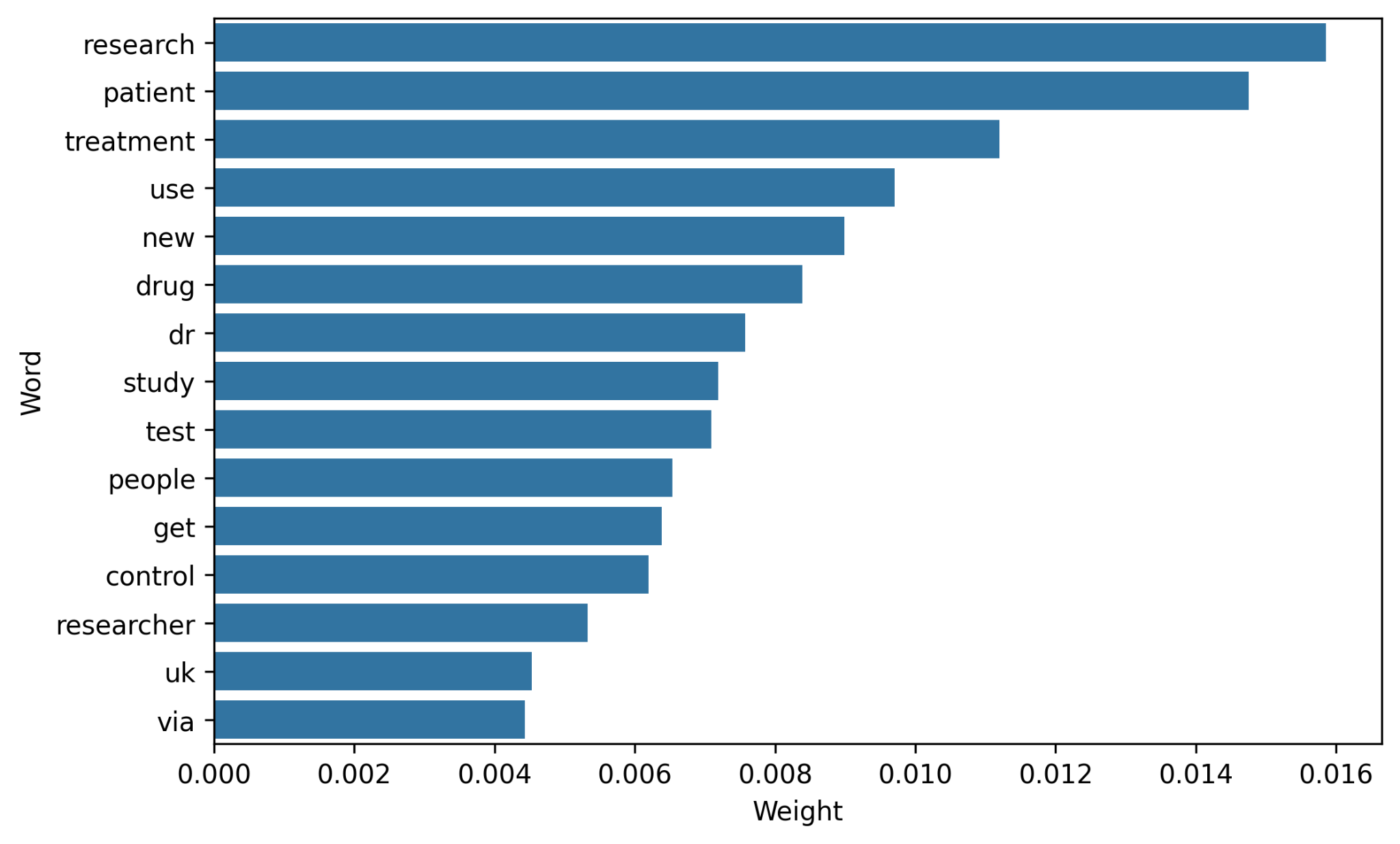


# Figure S20. Pre-COVID LDA topic 2


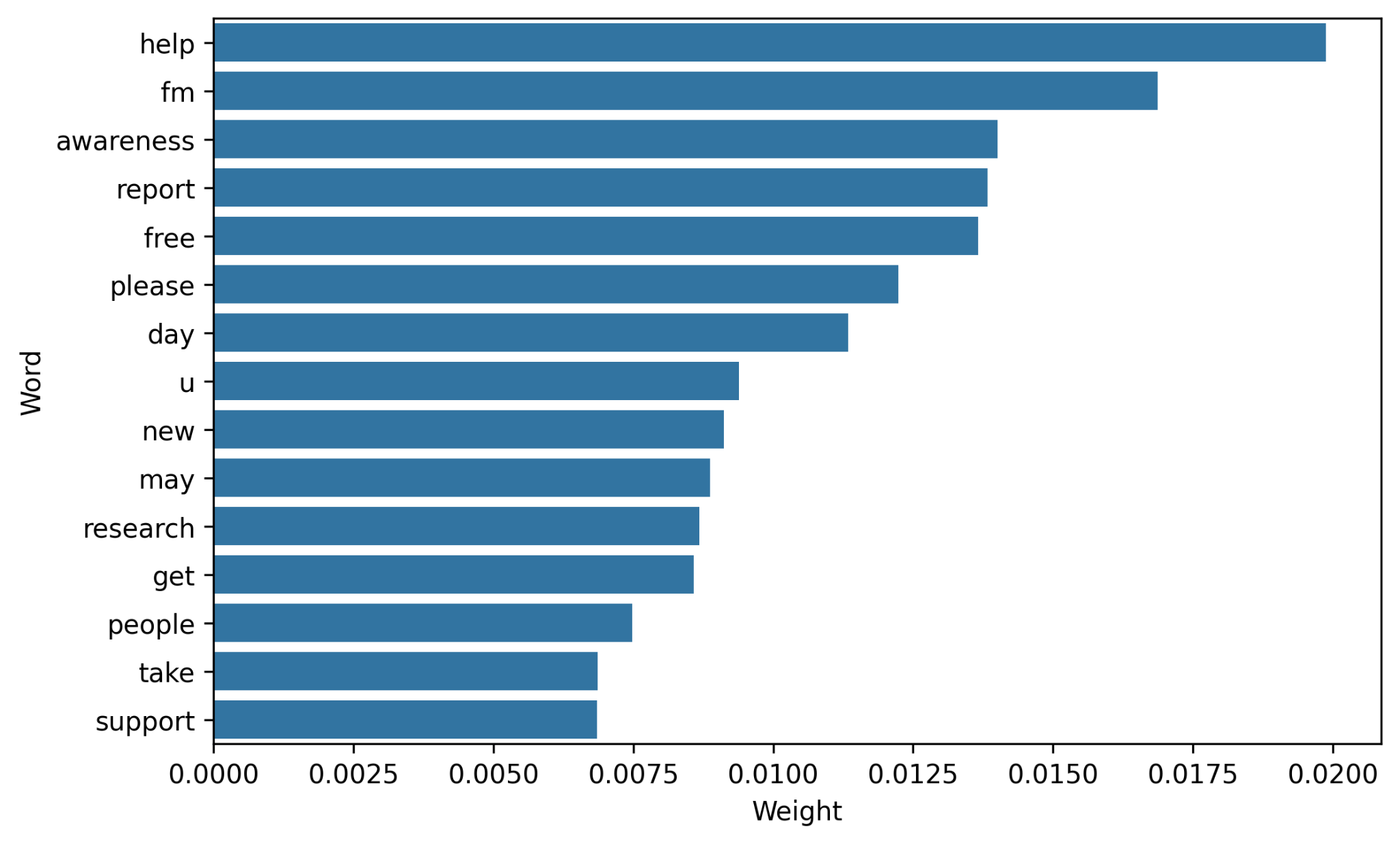


#

# Figure S21. Pre-COVID LDA topic 3


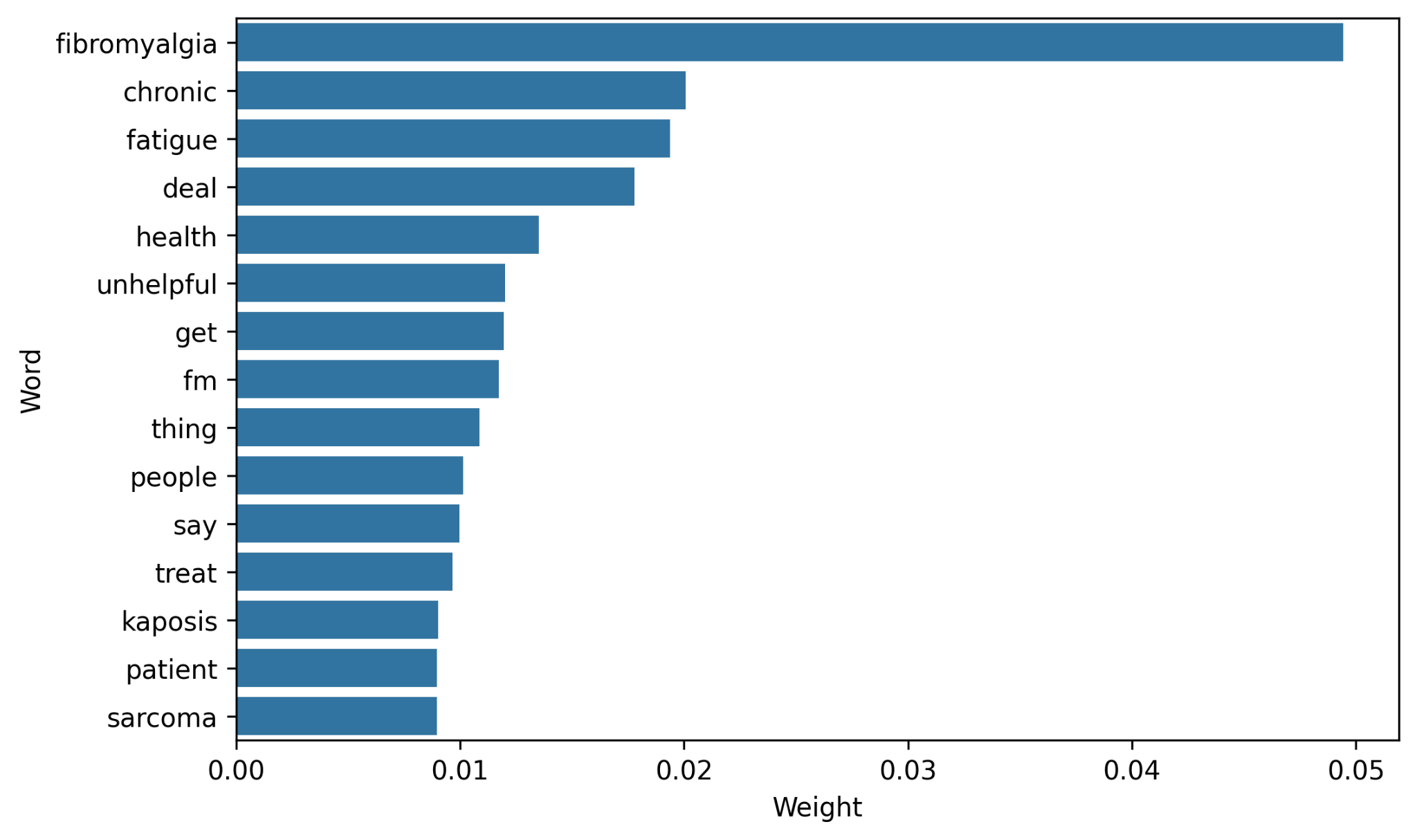


# Figure S22. Pre-COVID LDA topic 4


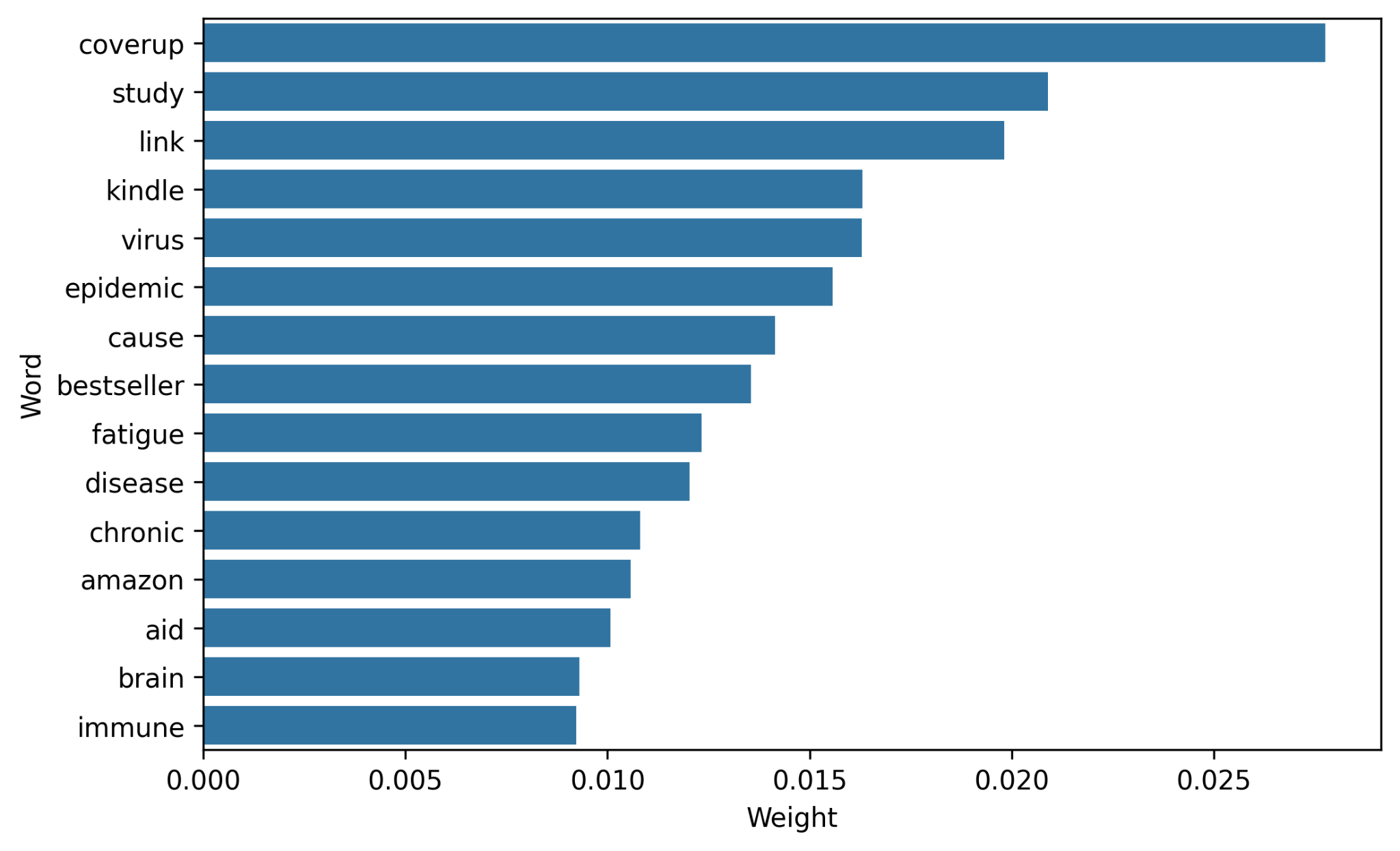


#

# Figure S23. Pre-COVID LDA topic 5


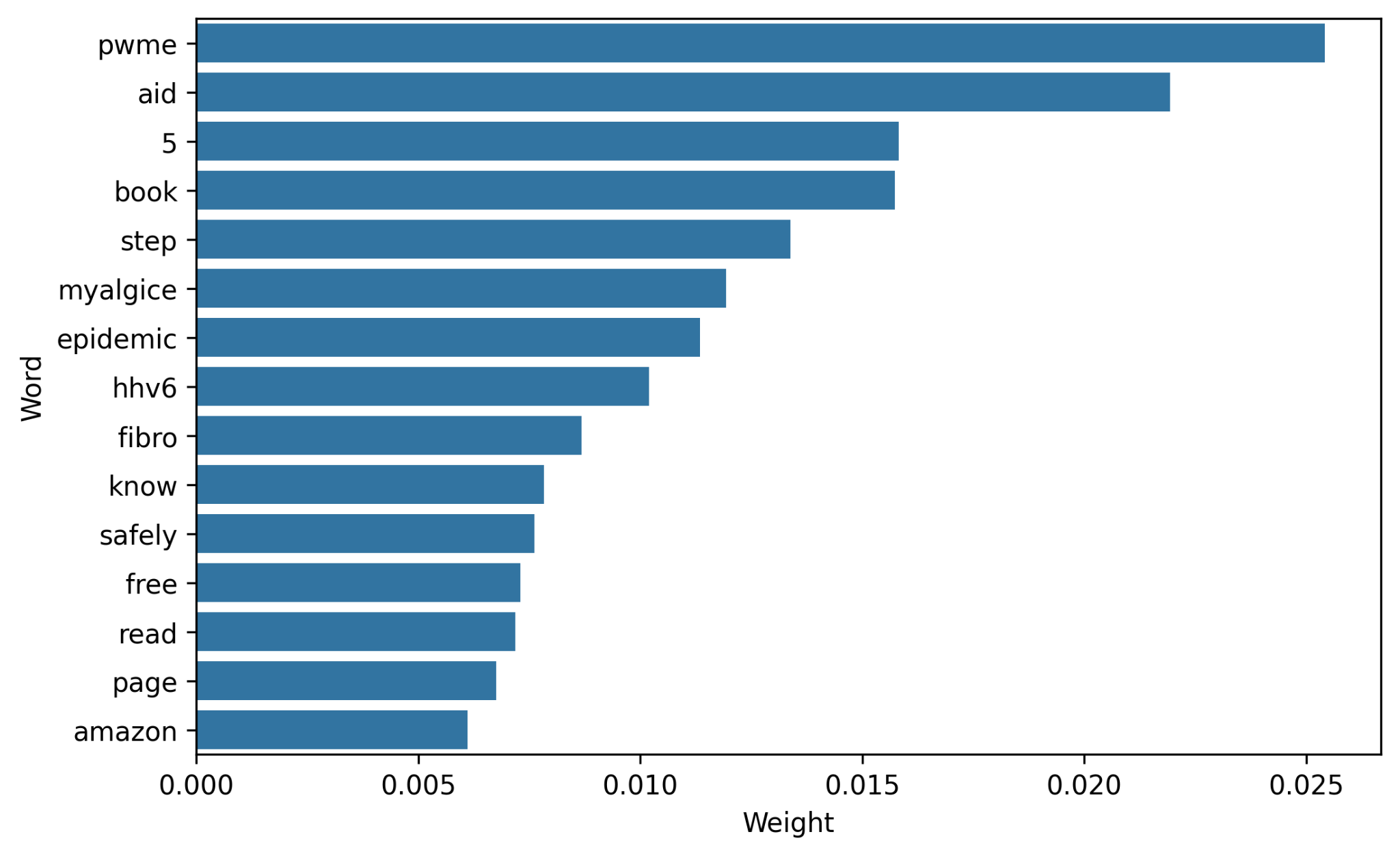


#

# Figure S24. Pre-COVID LDA topic 6


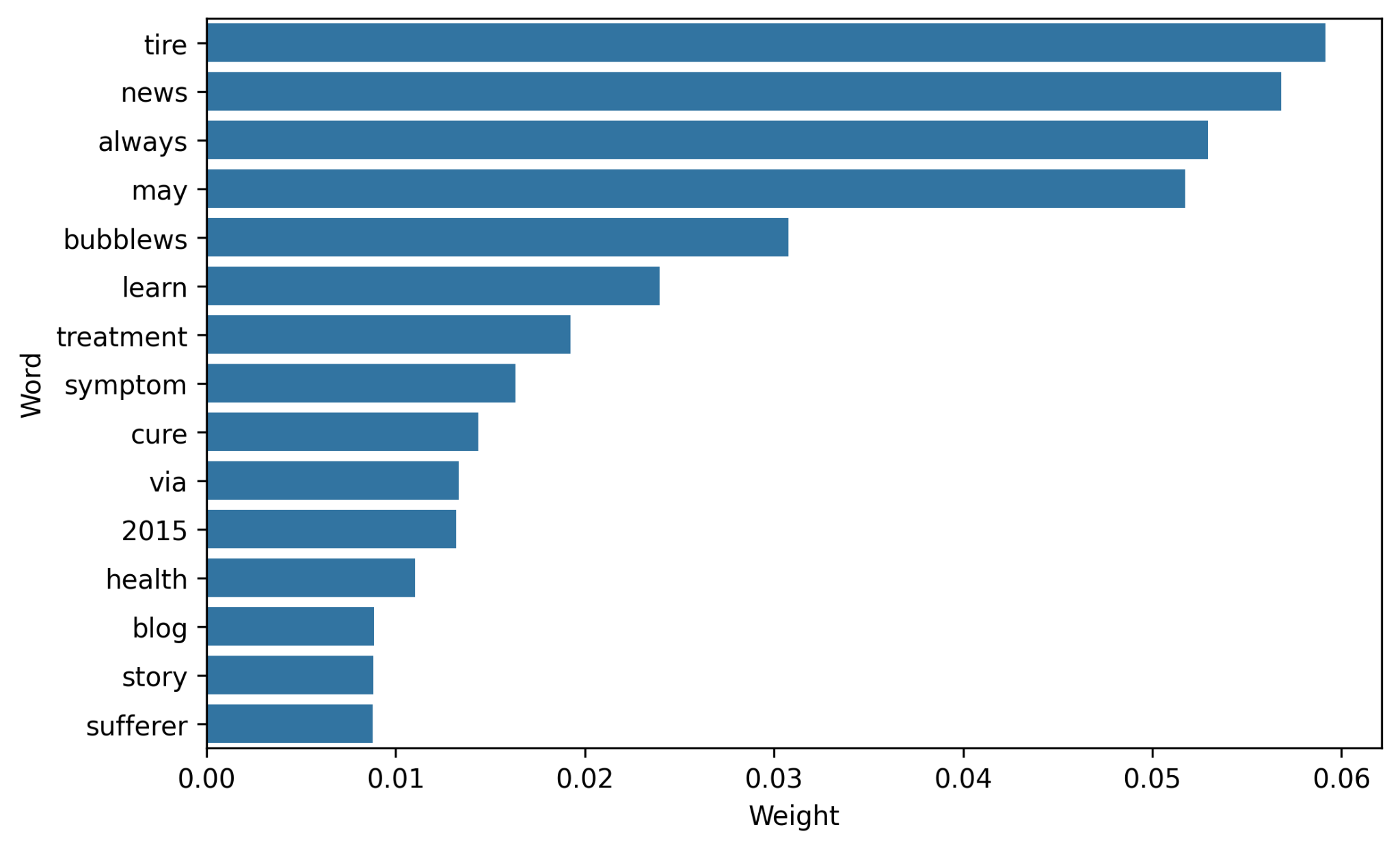


# Figure S25. Post-COVID LDA topic 1


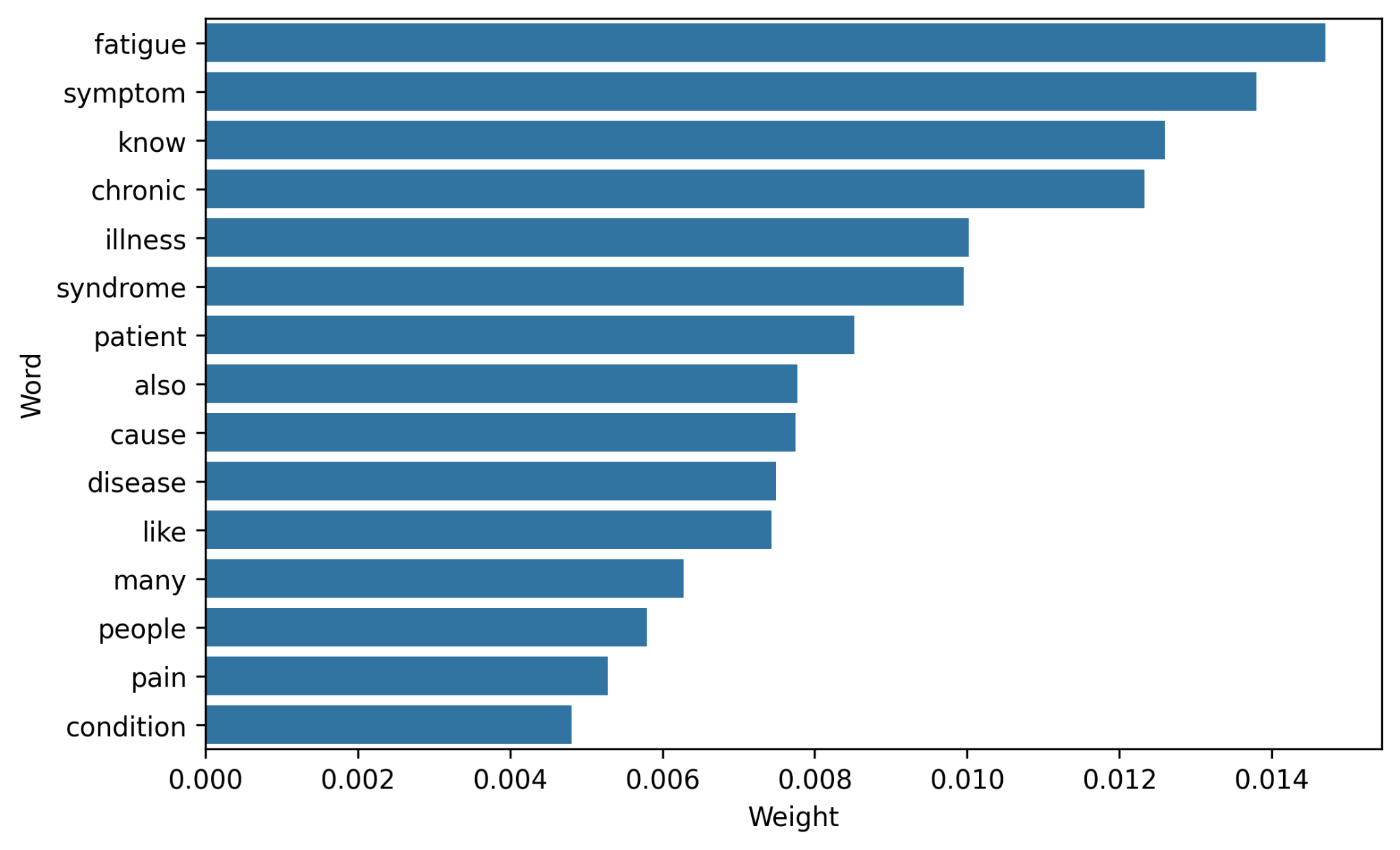


# Figure S26. Post-COVID LDA topic 2


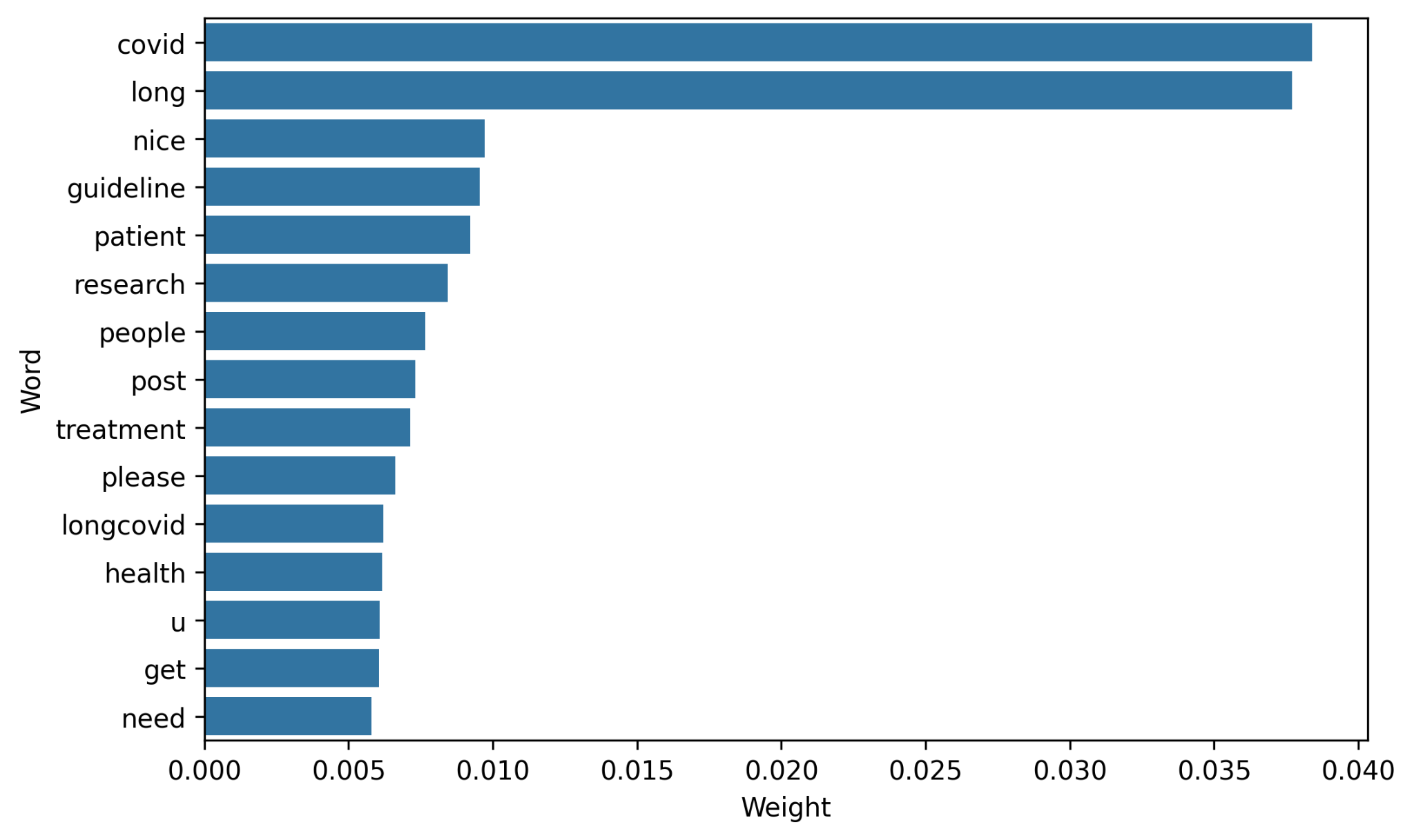


# Figure S27. Post-COVID LDA topic 3


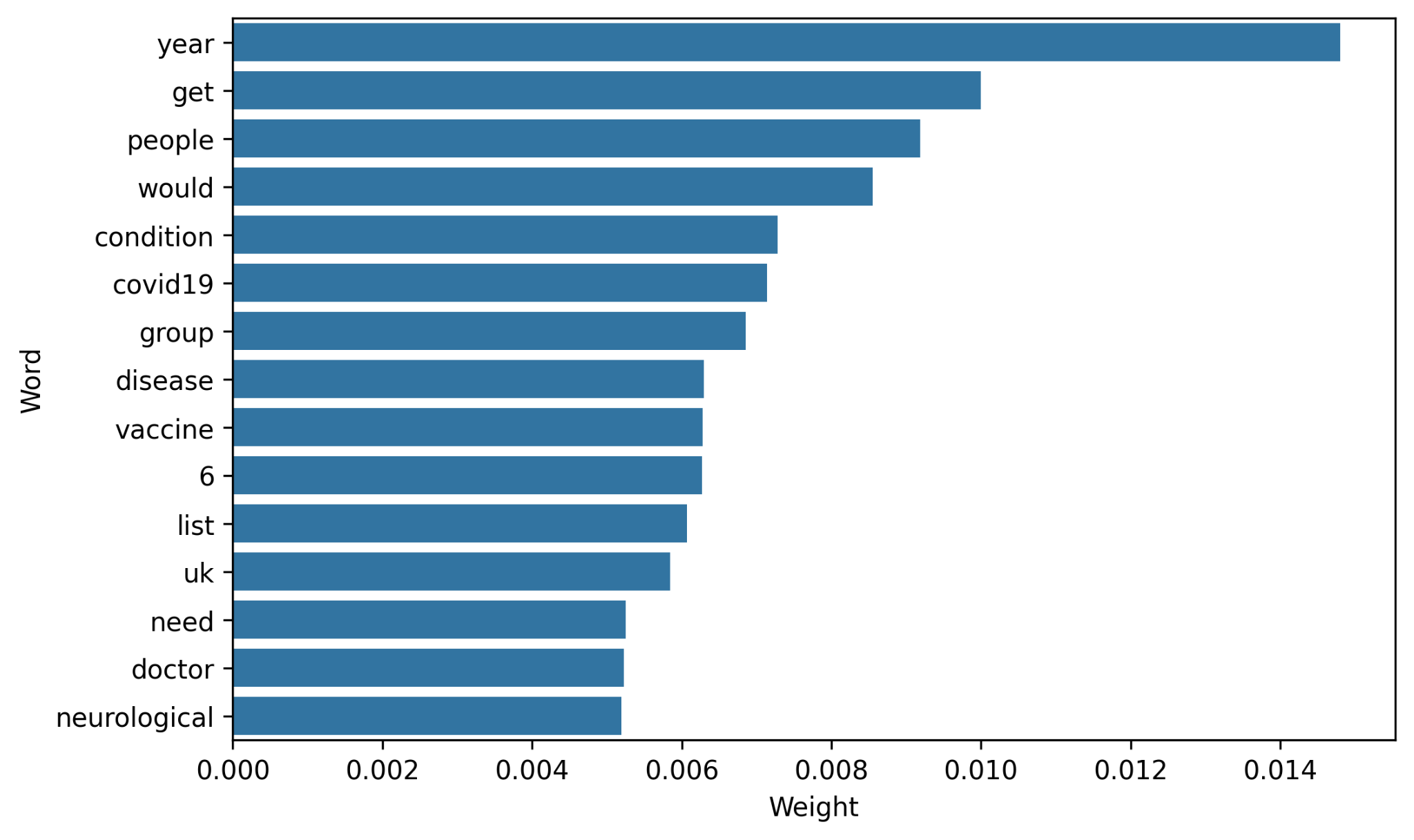


# Figure S28. Post-COVID LDA topic 4


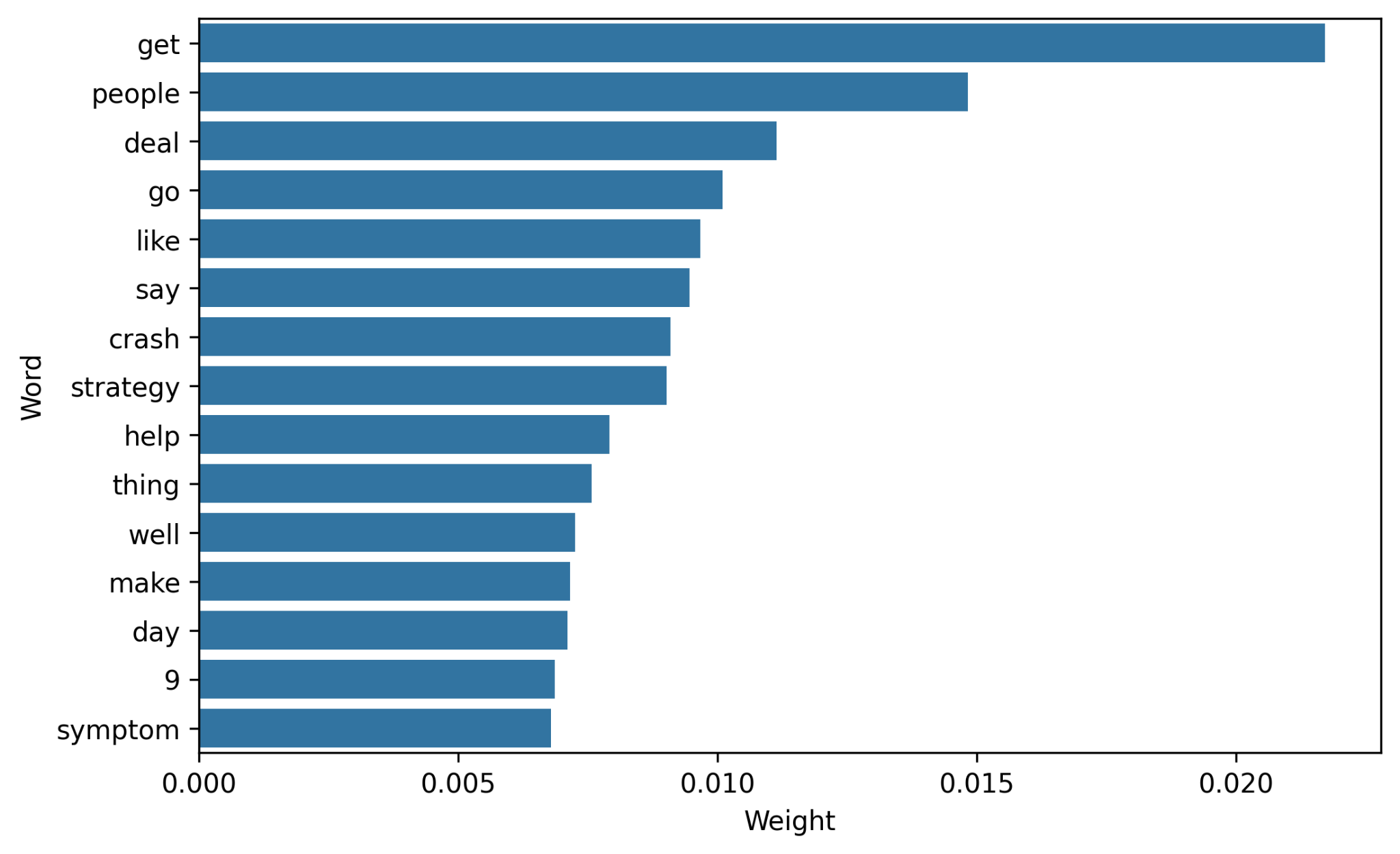


# Figure S29. Post-COVID LDA topic 5


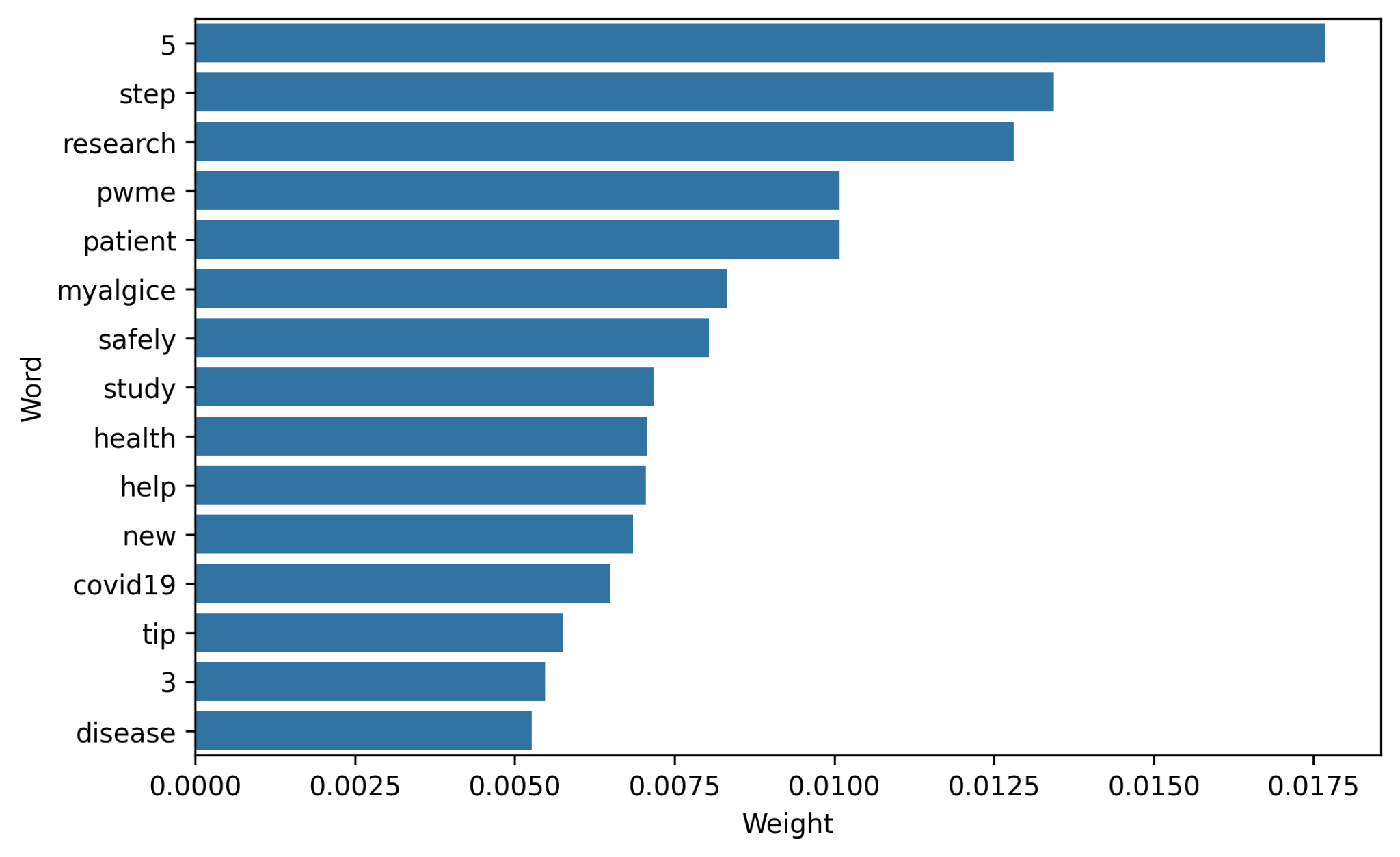


# Figure S30. Post-COVID LDA topic 6


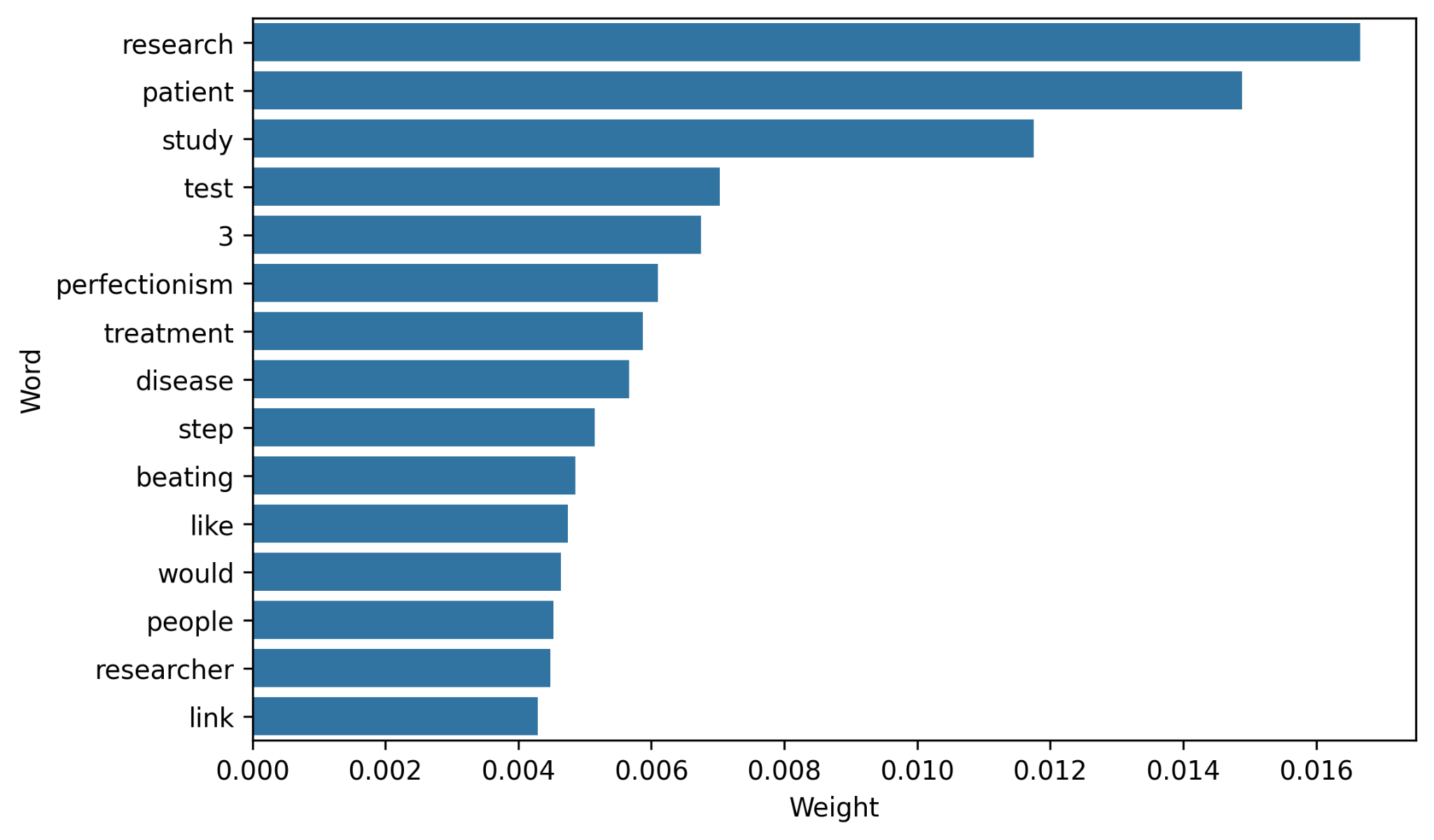


# Figure S31. Post-UK NICE guidelines LDA topic 1


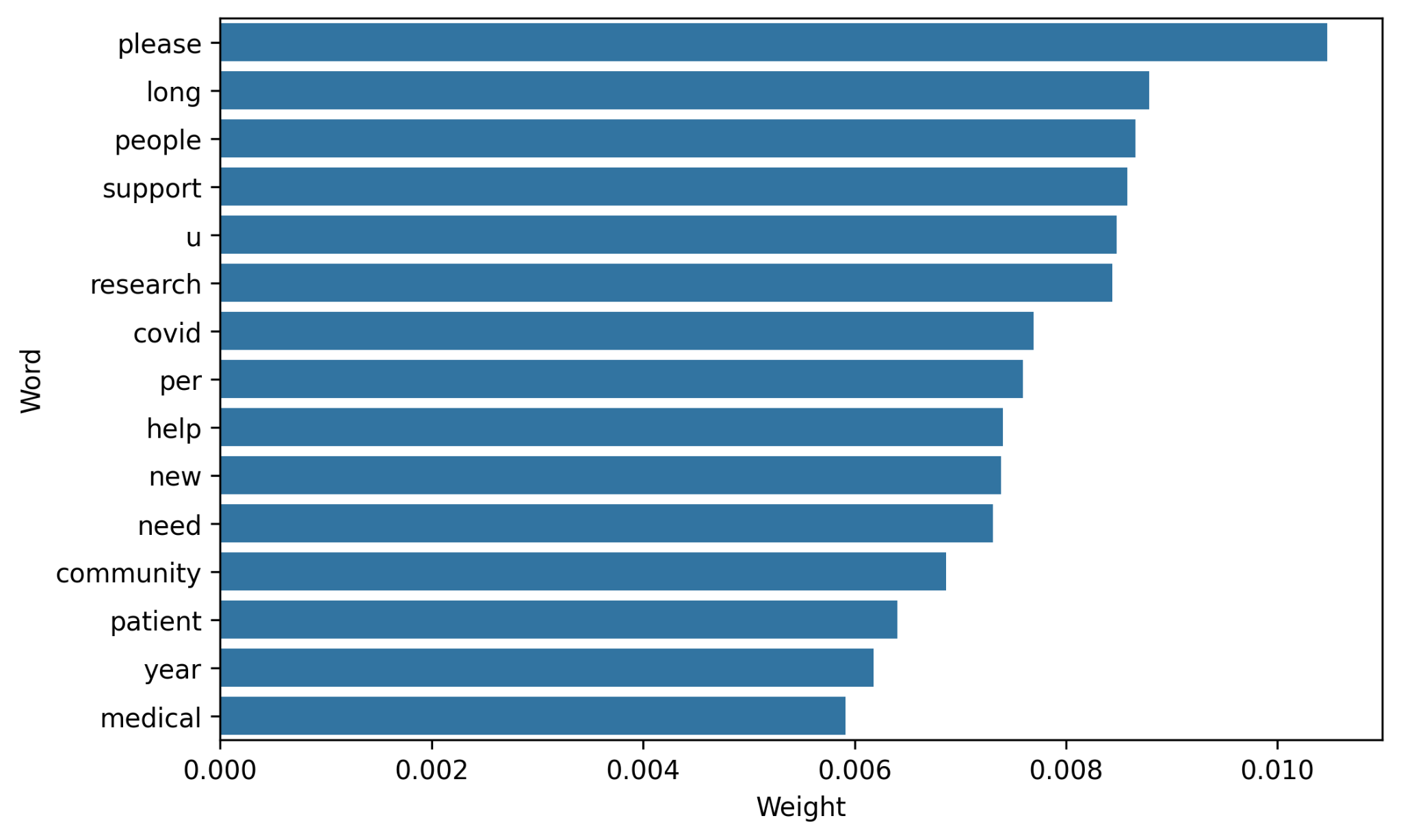


# Figure S32. Post-UK NICE guidelines LDA topic 2


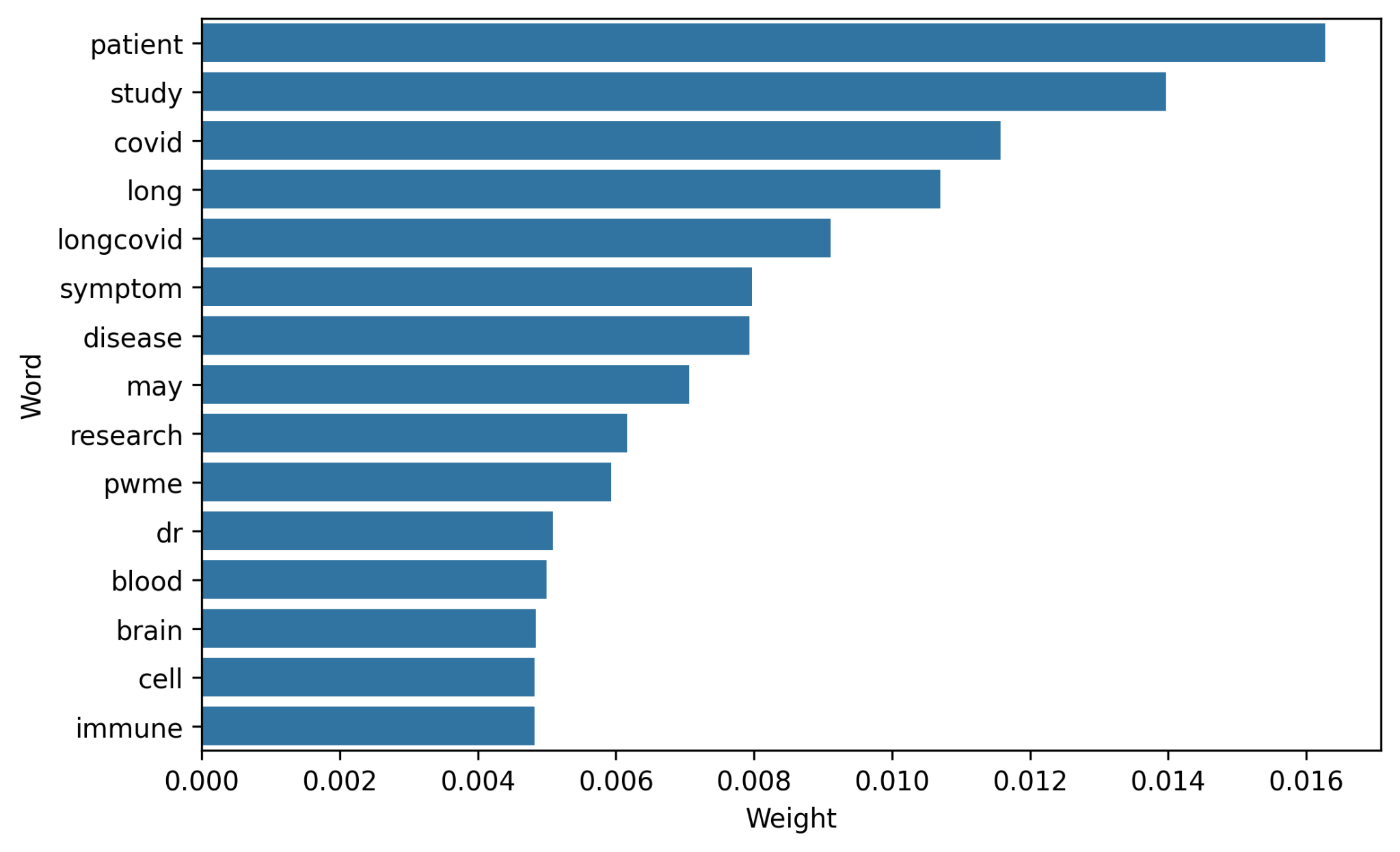


# Figure S33. Post-UK NICE guidelines LDA topic 3


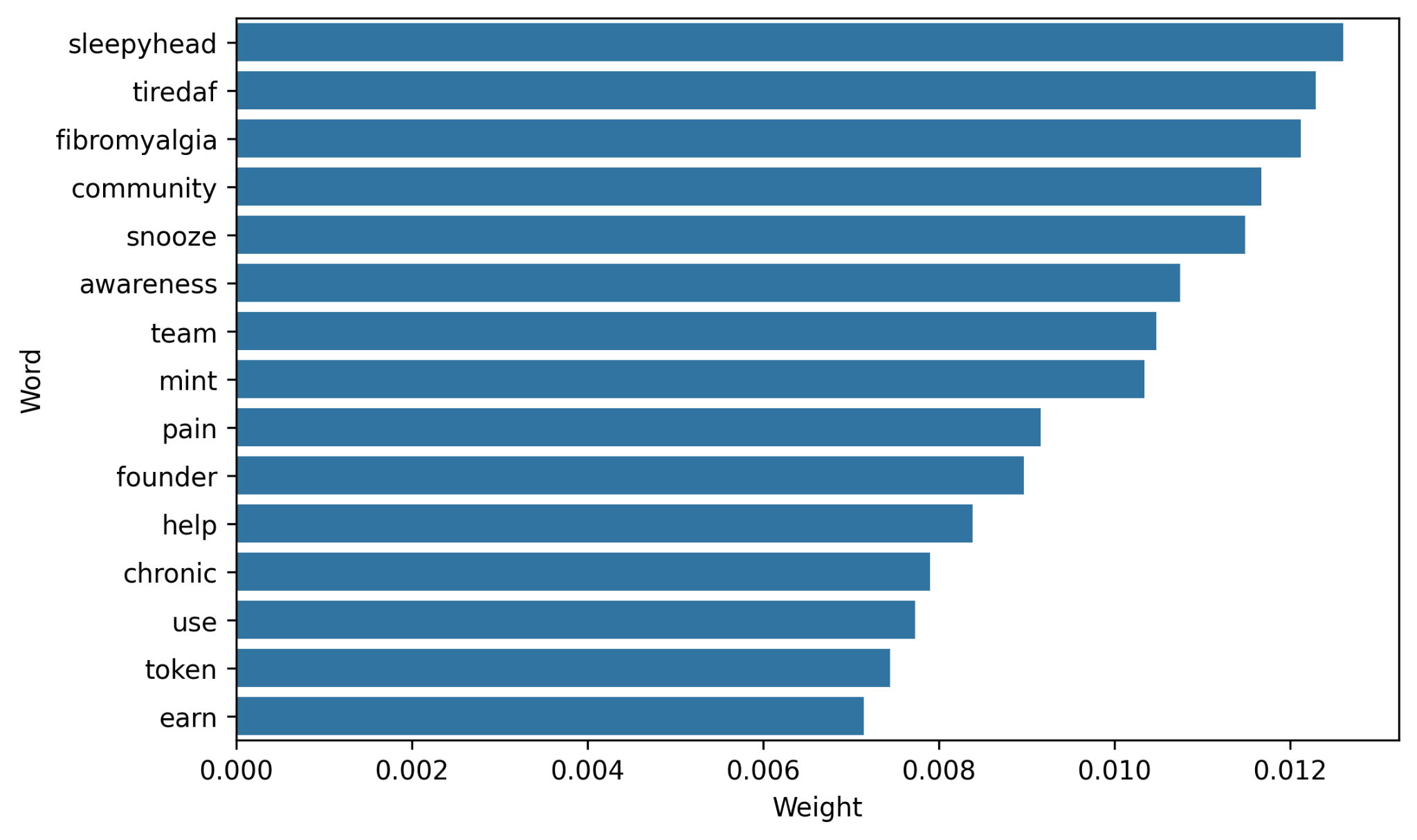


# Figure S34. Post-UK NICE guidelines LDA topic 4


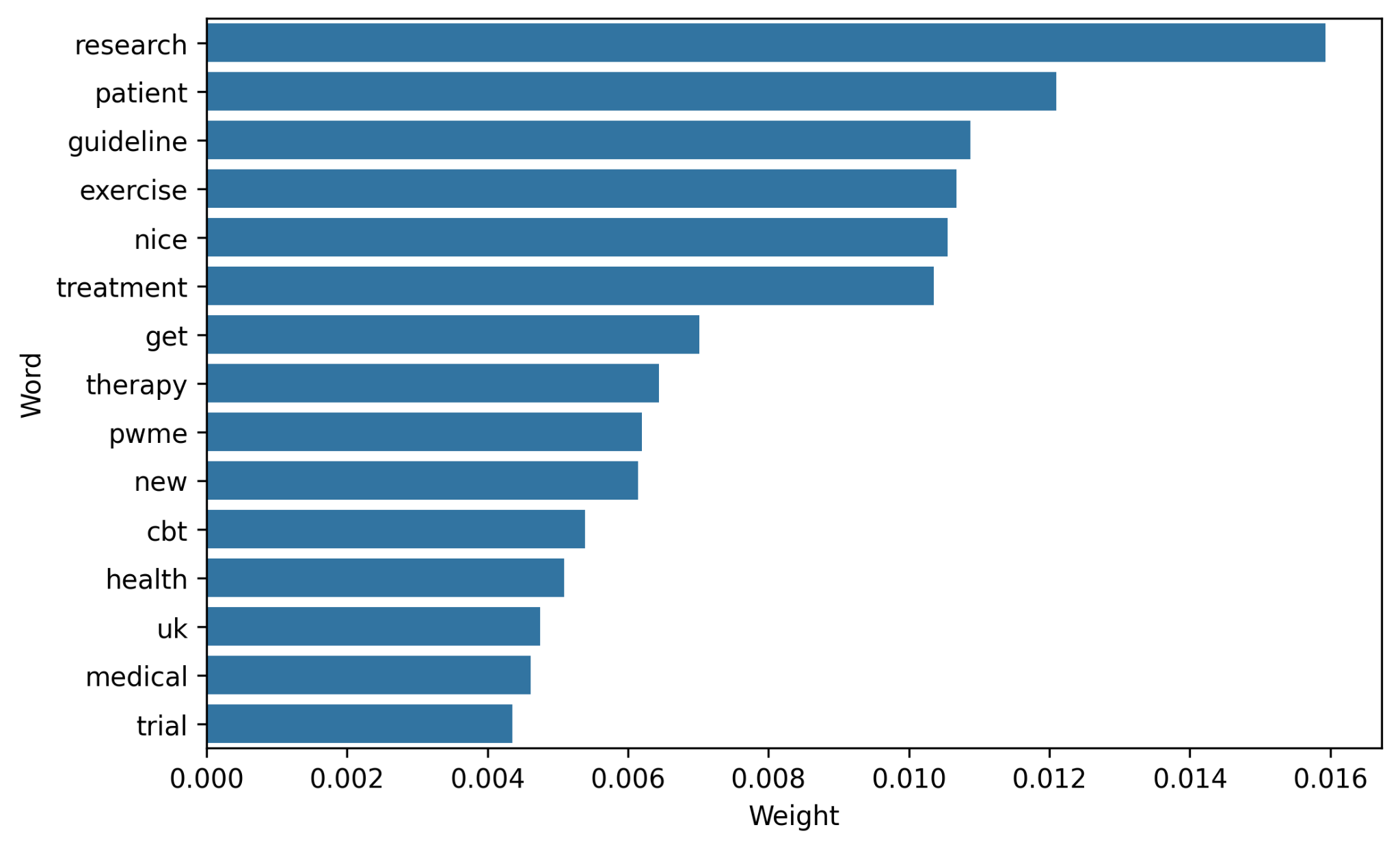


# Figure S35. Post-UK NICE guidelines LDA topic 5


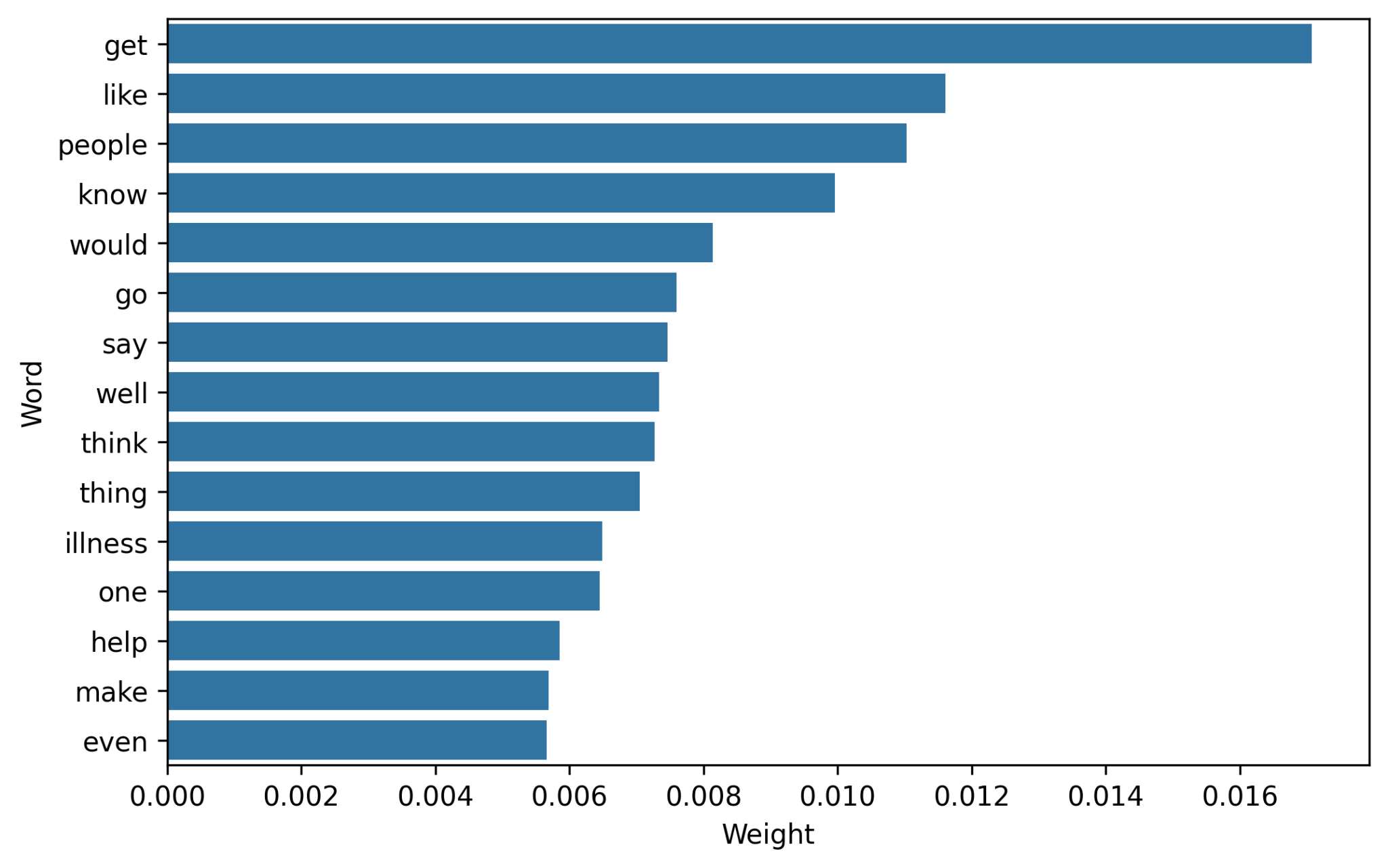


# Figure S36. Post-UK NICE guidelines LDA topic 6


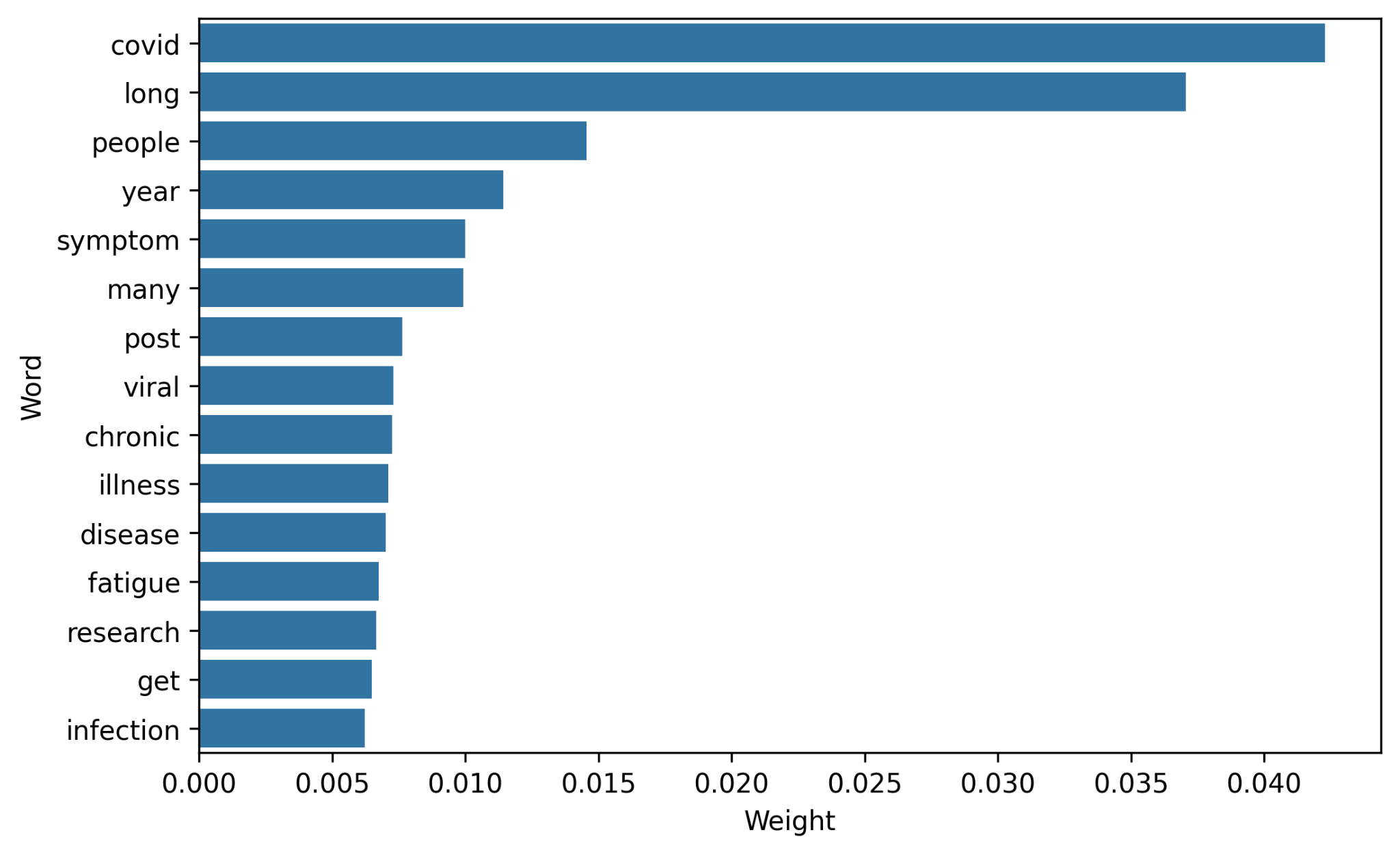


# Table S1. Representative tweets for ME/CFS and fibromyalgia

| **Subtheme** | **Representative Tweets** |
| --- | --- |
| 1. Frustration with dismissal of fibromyalgia and ME/CFS as psychological or psychosomatic illnesses | - I can’t even count how many conversations I overheard between my parents during the 90s about how chronic fatigue syndrome and fibromyalgia were fake diseases for depressed, lazy, overweight, listless women with no other conflicts in their lives. - Everyone here going “but chronically ill people can be evil too” is missing the significance of her specific choice of chronic fatigue syndrome, fibromyalgia, and POTS – all bear a unique stigma of being fake disabilities that whiny young people claim for attention. - I’m sorry. Same here. I’ve had arthritis since I was 6. I was told that I was faking my pain. My mom was convinced that I was a hypochondriac. Every time I was ill for any reason, she’d tell me that I was lying. I developed fibromyalgia and chronic fatigue syndrome later on. - I want to believe that this article is written from the perspective of someone who just wants to get at the truth, but… it reads a lot like the rhetoric people used to dismiss ME/CFS and fibromyalgia (“not biological” in particular). - I sincerely hope you're right. However as someone who's been dealing with fibromyalgia & me/cfs for the past decade I see the same patterns here - people taking the mick, acting like it's not real, laughing about it, acting like we're drama queens or just anxious. We'll see. - I like how my entire extended family has decided that it's all my fault for not being positive and taking initiative and just exercise what is diagnosed as fibromyalgia with signs of me/cfs, plus the downstream mental issues of chronic pain all away - Even dodgier is that for years people who have symptoms of Chronic Fatigue Syndrome or Fibromyalgia after a period of illness have been told 'it's in their head' for years and struggle to qualify for support, but now it's convenient for a similar condition - I have Chronic Fatigue Syndrome and Fibromyalgia. It's been that way since I was a kid. Nobody understands. Even my family, who was THERE when the doctor diagnosed me sometimes thinks I'm "faking" not "having enough energy." But it's everything, I need ENERGY just to TALK. |
| 2. Frustration with quality of care for ME/CFS and fibromyalgia patients | - Given the history of denial from so called medical professionals I’m not surprised, Fibromyalgia, ME, Chronic Fatigue Syndrome are just a few - From diagnoses like “this is all in your head”, to allergies, to fibromyalgia, to depression, to chronic fatigue syndrome, to EBV, to “an awakening”? i literally couldn’t handle the mixed opinions nor could i take any of it seriously. I’m analytical & i needed a clear roadmap. - I’ve been saying this since the since it was first reported. #Fibromyalgia, #ME/#CFS etc. Proper tests need to be a priority - Doctors are so afraid of patients who have symptoms of FMG (fibromyalgia) or CFS (chronic fatigue syndrome)Because they have no clue what are causes or how to treat. I now believe years of shots may be part of the causes. For many reasons. - Chronic fatigue syndrome (#CFS) and #fibromyalgia are two of the most common chronic health conditions that traditional Western medicine has failed to solve. It can be enough to make a person feel helpless. Know that #help is available! https://t.co/xKRVzO1Epu <https://t.co/SM7tfBRZ9j> - Classic doctor. Not surprising at all if you have any experience of ME/CFS or fibromyalgia or other conditions they can't diagnose easily. - Before 2020 “Long Covid” was called chronic fatigue syndrome, post exertional malaise, fibromyalgia, mental illness or something else that doctors couldn’t begin to comprehend….“Long Covid” is just repackaged vaccine adverse reactions…. - My experience has been that the average person or doctor thinks CFS is the same as fibromyalgia. The people who have never a seen a moderate or severe person with ME/CFS have no clue. And because of many of us can’t go out, we are never seen or understood. - That's one thing I'm dealing with. One doctor said fibromyalgia. Another said fibromyalgia and chronic fatigue syndrome. Others look at me like I'm crazy. All I know is, I hurt all the time and I'm always tired. I'm just sick of them not listening - I feel ya. I have fibromyalgia and chronic fatigue syndrome for the last 20 years. It was horrible to find a doctor to take symptoms seriously in the beginning - Doctors are so afraid of patients who have symptoms of FMG (fibromyalgia) or CFS (chronic fatigue syndrome) Because they have no clue what are causes or how to treat. I now believe years of shots may be part of the causes. For many reasons. - ME/CFS is better known in the UK and the US. I think it’s often associated with fibromyalgia in France, but CFS in fibromyalgia is understood as chronic fatigue (Fukuda criteria), not as post-exertional malaise. Anyway French doctors classify it as a psychosomatic disorder. - Yeah. .. not only my fibromyalgia and me/cfs were deemed psychosomatic, but even my gallstones 17 years before they were finally correctly dx'ed and removed even got to spend 6 weeks in a psych ward cuz of the gallstone puking |
| 3. Dissemination of research (or the lack thereof) investigating ME/CFS and fibromyalgia | - I'm a long time me/cfs patient in California. Curious if you have heard of Dr. Charles Lapp at the Hunter Hopkins clinic in Charlotte, N.C. He's a longtime researcher and treating physician for me/cfs and fibromyalgia. - I think you are all on the back foot as researchers for long Covid as the similarities to the extremely underfunded chronic fatigue syndrome and related fibromyalgia conditions are completely inadequately diagnosed and treated. You have a long, long way to go to understanding. - Chart reviewing some of the vascular / circulatory / reduced blood flow / hypoxia factors in ME/CFS & Fibromyalgia. - Sleep Problems in Fibromyalgia & Chronic Fatigue Syndrome: By Adrienne Dellwo, About… <http://t.co/NGtsTzSn> - He had ME/CFS himself and developed his own strategy and recovered. He wrote a book 'Managing Chronic Fatigue Syndrome and Fibromyalgia' and he ran a support group for many years. I've read about half of his book - it's very readable - nice big writing and plenty of paragraphs. - However similar the symptom presentations of #fibromyalgia (FM) and chronic fatigue syndrome (ME/CFS) are, their research pathways have been fairly distinct, with FM researchers focusing more on the central nervous system and ME/CFS… <https://t.co/pGeguVq4EM> - I was diagnosed with Fibromyalgia by a rheumatologist at a Boston teaching hospital 35 years ago. It gets scant research $ in the US, treatment options are poor. This is the gut punch for me: "people 'who say' they suffer from chronic-fatigue syndrome, fibromyalgia, chronic Lyme - I want to believe that this article is written from the perspective of someone who just wants to get at the truth, but… it reads a lot like the rhetoric people used to dismiss ME/CFS and fibromyalgia (“not biological” in particular) - Growing evidence that long-term conditions like myalgic encephalomyelitis/chronic fatigue syndrome (ME/CFS) and fibromyalgia are caused, wholly or in part, by viral infections is good news for millions of people <https://t.co/Nbov0gMAfp> - NK cells being dysregulated to attack snf is postulated to be the cause of fibromyalgia, ME/CFS, POTS, and Long Covid. Uncleared virus and dysregulated immune systems are the precipitators. <https://t.co/F8GrnXYQxs> - New Research: Psychiatric disorders and the onset of self-reported fibromyalgia and chronic fatigue syndrome: The lifelines cohort study: IntroductionThis study aimed to assess whether psychiatric disorders predict the onset of fibromyalgia… https://t.co/r2VrG6uytg #Psychiatry - Research suggests that myofascial release combined with massage therapy can be helpful in a short period of time for most people who present with #Fibromyalgia or #chronicfatiguesyndrome. <https://t.co/rmbdoJT6vA> - Clinical overlap between fibromyalgia and myalgic encephalomyelitis. A systematic review and meta-analysis. <https://t.co/7VKCfeRZPR> - I’ve joined OMF's StudyME registry to help advance research for ME/CFS, Long COVID, Fibromyalgia, and related illnesses. It's a global network of people and researchers working towards a cure! https://t.co/rKFGroh0DQ <https://t.co/GiHlsmVJQj> - If you've ever had a diagnosis of #CFS or #ME and live in the UK, register to take part in the world's biggest genetic study into ME/CFS https://t.co/wt8tSnMrUE #DecodeME #ChronicFatigue #Fatigue #Fibromyalgia #Fibro #MyalgicEncephalomyelitis #MCS #LongCovid <https://t.co/y1IIZBJi6S>   How many people agree that Long covid is rebranded chronic fatigue syndrome. What is coming is fibromyalgia, another disease doctors don't accept as real. #longCovid <https://t.co/Hi3QNZnB2N> |
| 4. Sharing personal struggles of ME/CFS and fibromyalgia | - Recovery was horrific. No bags tho thank god. I have 12 month immunotherapy to hopefully stop it recurring but that’s what caused my fibromyalgia and chronic fatigue syndrome. - I'm getting used to medication, found out I have ME/CFS & Fibromyalgia. - Could Green Light Therapy Help Fibromyalgia and ME/CFS? - Think of both Fibromyalgia and Chronic Fatigue Syndrome as 'ENERGY CRISIS' disorders. The goal of therapy is to… - Same hear. I got ME/CFS in the '90's, and it turned into fibromyalgia in 2002. (Fibro is like ME/CFS with a lot more pain.) I stopped having severe pain that required opiates several years ago, but I'm still not healthy. I wouldn't wish it on my worst enemy. - I have Myalgic encephalomyelitis, I have serious neruological problems, Cardio vascular problems. Fibromyalgia, Hashimotos, Orthostatic Intolerance, tinnitus, digestive problems, connective tissue disease, and a spine that is collapsing. Not all developed at the same time. - Jump ahead five years (right), I had lost over 50 pounds, was almost completely bedridden, was too weak to sit up, talk, or feed myself. I was diagnosed with fibromyalgia and myalgic encephalomyelitis/chronic fatigue syndrome, two illnesses that we still know very little about. |
| 5. Online support circles for patients with ME/CFS and fibromyalgia | - Check out @FibroFlutters - an informal ‘patient-led’ support group based in Sunderland for people with Fibromyalgia, Me/CFS, Chronic illness. - Bulletin Board: Support Group for Chronic Fatigue Syndrome (CFS). 2 to 4 pm Fibromyalgia (FMS), & Orthostatic In... http://t.co/F0ljATuT - Bulletin Board: Chronic Fatigue Syndrome and Fibromyalgia Support Group, 2:30 pm at Memorial Hospital West Fitness... http://bit.ly/bZoQo9 - From @MassMECFS e-newsletter: A Caregiver's Perspective on supporting ME/CFS partner caregivers that meets online monthly. - ME/CFS & Fibromyalgia Around the Web : FightingFatigue.org: Flupirtine is a widely known analgesic drug that is ... http://bit.ly/h4b7E6 |
| 6. Recommended treatments for symptom management of ME/CFS and fibromyalgia | - Could Green Light Therapy Help Fibromyalgia and ME/CFS? https://t.co/zPxuVfjr0D - Think of both Fibromyalgia and Chronic Fatigue Syndrome as "ENERGY CRISIS" disorders. The goal of therapy is to... http://t.co/GvogsYyU - Its a tricky topic because I know, from many years contending with fibromyalgia and chronic fatigue syndrome, that exercise…the very thing you might expect to help with recovery from physical injury…can very-much make things worse. https://t.co/69hngK2zs3 <https://t.co/o16dJOy7nY> - How To Beat Fibromyalgia And Chronic Fatigue Syndrome In 30 Days. <http://url4.eu/12ACL> - I have ME/Fibromyalgia/chronic fatigue syndrome I work because work is the best thing you can do for my condition. If I stop and give into it then I end up not able to move and in chronic pain. I have a physical job and exercise is good for me. No need to give up work! - Vitamin D for Fibromyalgia & Chronic Fatigue Syndrome <http://fb.me/vgWJlIeu> - A Methylene Blue Boost? Could a Blue Dye Help with ME/CFS, Long COVID and Fibromyalgia? <https://t.co/r8uUNO6WEW> |

# Table S2. Representative Tweets for Treatment of ME/CFS

| **Subtheme** | **Representative Tweets** |
| --- | --- |
| 1. Doctors are mis-informed and/or do more harm than  good | - This applies especially to ME/CFS patients. Their medical issues, their symptoms, are dismissed & psychologized. Doctors, often with little/no knowledge of #MECFS, choose to weigh in w/ an opinion on a complex, multi-system illness w/ a unique biochemistry. This is an error. - DYK that before COVID, graded exercise therapy was proven to be harmful to people with me/cfs and forms of dysautonomia? If you have LC, do your research before trusting your doctor about returning to exercise. They are likely to recommend actions that could seriously hurt you - ME/CFS has a damaging & very real PR problem. It matters - not just to general public - but also to broadly uneducated doctors, that an incapacitating illness is described so flippantly. If they don’t even accept severity of mild, you can bet they’ll never appreciate v severe. - Not a very high percentage, unfortunately. I think the average doctor has a fairly rudimentary understanding of it, and/or lacks the time and resources to properly apply it. That was certainly my experience with every single doctor (ME/CFS), and I had to figure it out myself. - If your Doctor is dismissive and unhelpful, consider getting another Dr & making a complaint - The other side of the coin is doctors assuming they know more about your condition than you. I reversed fibromyalgia, chronicle fatigue syndrome, opiate addiction and chronic benzo use thanks to google. I’ll keep lecturing ignorant MDs - A significant percentage are suffering from syndromes that few doctors understand or treat, primarily postural orthostatic tachycardia syndrome (POTS) and chronic fatigue syndrome (CFS). - Hugely agree with this! Even with things like sleep/circadian rhythmn disorders, being afab with adhd/being autistic, conditions like ME/CFS, fibromyalgia, like I have all of these and every time I talk to a doctor they either don’t know anything or suggest harmful treatments - It’s because regular doctors have no clue what post viral fatigue or ME/CFS is. Exercise can be harmful even if organs are fine. - Sorry to hear… Unfortunately the majority doctors know very little of ME/CFS and long-covid, and we as patients have to be our own doctors unfortunately. It's a horrible medical scandal. - Reading this article about the DNA for multiple sclerosis being borne on horseback across the steppes has me wondering ... is MS being ignored the way ME/CFS has been for years? Another "mysterious" disease that doctors don't understand the mechanism? - I just LOVE idiot doctors who think they know more about ME/CFS than I do. None of them understand PEM, and always think I’m exaggerating. I have 40 years of experience with this, and they’re all morons. - The PACE authors who authored this paper ask the question, should GET, graded exercise therapy, be prescribed to ME/CFS patients, and answer YES, when @NICEComms UK were reviewing evidence on GET and sent out a report saying GET to be removed! This paper now misinforms doctors. - Believe me after 40 years of ME/CFS, I know female doctors can also be arrogant and dangerous - Was told by a doctor to stop using my wheelchair as it was making me weaker and I needed exercise. But my chronic fatigue syndrome hasn’t ever been so severe before & if I hadn’t had a wheelchair I wouldn’t have been able to get out of bed for the hospital appointment anyway - But most people with HS don’t get that lucky, and can spend their lives in pain as doctors tell them to do things that are actively harmful. - Sadly it's the fight people with ME/CFS still face. Very little good quality research, no effective treatment and a complete lottery of whether a doctor will understand. - Of course not all GPs. But 2 GPs this year alone have recommended GET. It's problematic. Of course GPs are overwhelmed but ppl with #ME #CFS have been dismissed, ignored and given the wrong advice for decades. I don't see/ hear any improvement esp now with long Covid patients. |
| 2. ME/CFS is mislabelled as mental illness | - My doctor will be as much use on this as talking to a stuffed bear, they know nothing about ME/CFS whatsoever, other than its obviously just anxiety or fake. ME/CFS patients don't get the luxury of healthcare, we aren't eligible because doctors deny us access. - I got ME/CFS after a seriously bad case of tonsillitis at 20. My doctors thought it was depression and increased my antidepressants. Surprise! That didn't help :) It took my Mum a while to get it but once she did, she was heartbroken for me and supported me fiercely. - Doctor said I was just depressed. I was doing everything I could to get better. Depression wasn't cause - Most other doctors, those who are not scrambling to specialize in LC & ME/CFS, which is the vast majority of the medical field, will have no idea what to do with you. They'll tell you it's all in your head, run a CBC and CMP, which'll show "everything's normal!" and send you home - Some doctors think it's a mental illness because they are arrogant, uninformed, and can't be bothered to read the biomedical research on ME/CFS. - Ah, that takes me back to the days of my illness before my ME/CFS diagnosis, when every doctor I saw was trying to ply me with anti-depressants, tranquilisers and sleeping tablets - So since - to these doctors’ minds - impairments cannot be attributed to the minor inconvenience of ME/cfs, they now latch onto the mental health diagnoses as explanation of all symptoms and impairments. - The ME/CFS community have battled the medical community for years who have said their condition is ‘all in the mind’ and PG is dragging them back years in terms of advocacy and campaigning. - Chronic fatigue syndrome, asthma, anxiety, depression, autism (& possibly other/different things bc doctors don’t know shit about CFS and just ascribe anything weird my body does to that!) - My niece was injured by the HPV vaccine aged 12. She collapsed after her second jab and developed POTS syndrome, chronic fatigue and had other symptoms. Doctors said it was anxiety or ‘ in her head.’ They are liars. - I've had ME/CFS for over twenty years. Doctors generally don't care to hear about illnesses they don't know how to treat. I've been told I'm perfectly fine, my illness is just in my head. I don't go to Drs anymore. They are no help at all. - Omg! That's sooo true. Before knowing about my ME/CFS and my POTS I went to the doctor stating I had panic attacks (what else could it be if my heart is racing). Without any further tests or questioning it was put in my chart and I got meds for it (that of course didn't help). - Suffering from PostViral syndrome through EBV. It's a pity, ME/CFS or PostViral Syndrome have been promoted as "only in your mind" for decades. There could be a cure already, if doctors weren't that stubborn and dismissive. - I have ME/CFS myself. This is a neurological disease. Since most doctors are not familiar with it, we sufferers are often labeled as mentally ill, although the symptoms are purely physical. We don't need deniers and agitators against us sufferers. - Lol okay so today was the first time a doctor suggested I might have, not depression, but chronic fatigue syndrome. That's what you wanna hear when there's a pandemic causing inflammation so severe that 4 of your acquaintances are getting their gall bladders out this month |
| 3. Dismissal of symptoms and concerns by doctors | - Not all other illnesses. Doctors and others tend to dismiss stuff as not real when they can't easily prove what's going on. ME/CFS has been treated this way for years. Almost anyone with a rare "mystery disease" will run into it from at least some doctors. - I have fibromyalgia, ME/CFS and POTS. I sooo feel you. Doctors won’t take me on because I’m “too complicated of a case” for their practice. Just wanted to say I see you, I get the same feelings sometimes, you’re not alone and sending gentle virtual hugs - Described variously as hysteria, patients as barmy and doctors not recognising it as a condition; ME/CFS can take away careers, marriages and all sense of living. - Many of us have other "acceptable" conditions or went to the doctor for routine things before we got ME/CFS or Long COVID, so we can see the difference, and I've been getting this for almost 2 decades. I'm probably understating it or being polite. There is an active rejection. - First you must know what ME is. It is not ME/CFS. ME was added to CFS because of a name change committee. It is not commonly known as CFS. Psychiatrists came up with that statement to bury ME. ME/CFS is SEID. It is not CFS. CFS is a lazy doctors way of not treating the patient. - Doctors, school, friends thought I was faking chronic fatigue syndrome - The absence of urgency by doctors to help is very familiar to me with ME/CFS: "You can't work anymore? *Blank look*.""You can't walk more than 15 minutes at a time? Try exercise. “You feel sick all of the time? Lose weight. “Chronic headaches? You do seem stressed." - Example number 579 why the Golden Girls were light years ahead of its time: the episode where Dorothy has Chronic Fatigue Syndrome. The dismissing of her legit medical complaints as simple aging or lack of social life by doctors who can’t find an easy diagnosis is spot on! - I believe the "God Doctor Complex" is the true reason no one ever looked into Chronic Fatigue Syndrome. This was something beyond their God Powers, so they went out of their way to ignore it, pretending all the while that they knew EVERYTHING about this. - CFS/ME (badly named 'Chronic Fatigue Syndrome') is an autoimmune illness we don't fully understand, sometimes triggered by viral infections like COVID. And because it isn't understood many doctors will tell sufferers that they are inventing the symptoms. So bleak! - I developed ME CFS 8 yrs ago. It has absolutely devastated my life & the real kicker is NO Duke doctors I’ve seen have a clue how to treat/support me or don’t believe in it at all. Being disabled is extremely hard but to be gaslit & dismissed makes it so much worse. - This condensed video of Dorothy being gaslit by doctors as she tried to find out that she had ME/CFS is heartbreaking because it’s so accurate. It’s quite triggering tbh. - That’s good to hear. I get unbroken sleep, sometimes, but I still wake every morning feeling broken. I think there’s something else, on top of the diabetes, going on (ME/ CFS?) but the doctors just blame diabetes and won’t even tolerate a discussion until it is under control - The leading Dysautonomia Doctor in my City doesn’t believe in PEM and since every other ME/CFS Aware Doctor follows his lead, I haven’t met a single Doctor who understand PEM in my City. Doctors in other parts of Canada do understand PEM. - I don't, and you surely have a point there. But the problem starts with the denial of Long covid and ME/CFS by many doctors, so what to expect of only waiting for studies, why not trust patients reports and help them. Speaking from Personal devastating experience BTW. - When I was a kid I was hit by a viral infection that resulted in all sorts of wacky health issues, it was suggested to my mother that the cause of the issues was her "over mothering" me. Twenty years later it gets diagnosed as ME/CFS which some doctors still think isn't real. - I can’t even get a doctor to properly document my symptoms & how they limit my functioning. they just wrote “chronic fatigue”, not even “chronic fatigue syndrome” and call it a day. - Doctors are all fired up about long COVID which is the same damn thing as ME/CFS which they didn't give a damn about for decades. They decided we were just hypochondriacs and most are women so it was medical misogyny. We suffered for decades very ill. |
| 4. The need for self-advocacy to find ‘good’ doctors or receive better treatment | - Others have told me to not mention Long COVID when I try to access care - to just talk about one symptom at a time. This is what ME/CFS patients report doing. Patients managing doctors and nurses in order to get proper healthcare is insane. - No one should go to dangerous doctors period. The more educated doctors get the more stupid they become. I am entitled to sue the medical establishment of Canada. They cause Chronic Fatigue Syndrome period. - I get their dilemma. Not an easy one for the doctors, but there are doctors who are willing to read the research & try outside the norm things. That’s who I went to for Chronic Lyme (& ME/CFS & Fibro dxes) & how I was able to actually get better, even after 11 years. - I tell doctors now, that I've a “post-viral syndrome caused by EBV“ & that it “mainly affects my immune system“ & it feels like “having a cold“ with “swollen tonsils & fever“. I'm also “tired through that“, “gets worse after activities“ (ME/cfs, NEVER say fatigue) - Yes, I teach them a great deal. I let them use me as test subject. What is special about my doctors is that we have a discussion about my health. We make decisions, choices together. Any recommendations for me/cfs, PEM, POTS. I'm willing to try & report results. - So, in order to not get trapped like most of us do (being sent to different doctors for different symptoms instead of looking into multisystem diseases), ask to get your blood tested for EBV, ask about ME/CFS and Fibromyalgia and where to turn to. - However, I'm super relieved (in disbelief) that after seeing a dozen+ doctors/specialists, I've found one who's actually assessing my ME/CFS and POTS!Like looking at my symptoms, considering possible diagnoses and causes, and starting me on an initial treatment - *jaw on floor* - You still deserve a complete and accurate diagnosis. I had a "somatic disorder" diagnosis from a local lazy diagnostician before I was properly diagnosed by leading experts in the ME/CFS field. In my book, FND is a lazy diagnosis. Find a doctor who works harder. - Finding a doctor that is both supportive & knowledgeable about ME, especially at the beginning, can mean the difference between a recovery, or years of worsening symptoms. Doctors attitudes & actions have the potential to change lives. - Most women have a story of not being believed by a doctor. I'm trying to teach my daughters to speak up without doubt. - I was pretty surprised recently when my doctor told me that my ME/CFS is physical rather than psychological. I have waited for this day for over 30 years. I thought there might be an apology for the misdiagnosis that has caused me so much agony. Or perhaps a little rueful. - I did an at-home Schellong Test with the help of a family member (all you need is being able to measure BP and HR at home) and then showed the results to my doctor (who is thankfully specialized in LC / ME/CFS so he prescribed it to me). - for sure!!! my mom has ME/CFS like me and she had really bad glandular fever in her diagnosis. but we are gonna speak to the doctor just to make sure he’s aware of my heightened risk for lymphoma to see if I can get a screening just to be sure - Help your doctors diagnose and treat your #MyalgicEncephalomyelitis #MECFS #Dysautonomia Keep a daily log of activities and symptoms. DePaul Symptom Questionnaires measure symptoms of ME/CFS, ME, and COVID-19. - There is treatment. It does help. It’s very complicated. And totally depends on *your* situation and biology. Try to find doctors who know about ME/CFS & post-viral illness. Pace!!! - Thankfully, my internist (my primary doctor for over a decade) is very supportive. He always considered CFS to be a serious biomedical disease and did his best to help me manage my symptoms. But like most docs in the US, he simply had never heard of myalgic encephalomyelitis. - I was diagnosed with fibromyalgia and chronic fatigue syndrome. I would recommend going to the doctor and tell them you want additional testing since they will just tell ya the same thing over and over. Take vitamin d as well!! Esp if you live somewhere without sun - Most doctors are not your friend if you have ME/CFS. Don't see them for that. See them for symptoms, never mention ME/CFS. They cannot help with that. - It's hard, you'd have to see a specialist and really advocate for yourself. Which isn't easy. I ended up seeing an internal medicine doctor and was diagnosed by them with ME/CFS. I think getting a diagnosis really help me accept the fact I needed to pace myself and slow down. |

# Table S3. Representative Tweets for Research

| **Subtheme** | **Representative Tweets** |
| --- | --- |
| 1. Sharing ME/CFS research | - New research linking chronic fatigue syndrome to retrovirus is released after being held by journal - <http://newzfor.me/?67x8> - https://t.co/SnPrpwmJR6OMEGA are hosting a talk by the pioneering Karl Morten on his latest research into the causes of ME and possible biomarkers. Do join us for free online on the 18th September. #ME #CFS #MEAction #Unrest - Griffith University researchers identify similar brain structure changes in both chronic fatigue syndrome and long COVID https://t.co/lhg1X1UW6I - Between long COVID, ME/CFS, and other energy-limiting chronic illnesses, millions of people in the U.S. alone experience debilitating fatigue. Researchers have started to figure out why—but don’t yet know how to stop it. <https://t.co/08bKsjbycR> - Decreased NO production in endothelial cells exposed to plasma from ME/CFS patients https://t.co/OTt3pRCIVt“Nitric oxide (NO) production was reduced in endothelial cells exposed to blood plasma from people with ME/CFS. |
| 2. Criticisms of ME/CFS research | - Google the PACE trial and then how many people with LC qualify for an ME/CFS diagnosis and then rethink. Please. - Fauci knows full well what Long Covid is, 2 years ago he said it “resembled ME/CFS”. Let’s not forget he actively blocked biological research into ME/CFS for 2 decades. That’s why he’s ducking for cover now. - My issue is not so much that Long Covid is ME/CFS, by definition in half of cases it is (causing agent of Covid). My issue is more the suggestion that research isn't worth it, we don't have treatments and that deserves pathology research. This is a BPS supporting article. - Like I say, this is hard for people to accept, but Dr Komaroff is not studying CFS at all.He is studying EBV syndrome and CALLING it CFS (or ME/CFS)Dr Komaroff never saw an outbreak and EBV cannot cause them. This is "mistake #1" about how all researchers got the story wrong <https://t.co/658EkkUmLd> - The #PACE study that claimed that graded exercise training (GET) and cognitive behavioral therapy CBT) were effective in treating Myalgic Encephalomyelitis. #MECFSThere were so many things wrong with this study that it's being used as an example of how NOT to do research. - That man I have permanently blocked. He claims to be an ME/CFS researcher. Almost all of them are corrupt and get paid to keep ME/CFS patients from finding out they likely are vaccine injured or sick as a result of a post infectious gain of function virus. - On behalf of pretty much everyone, because we are all susceptible to Long COVID, I would love for LC research to stop spending so much time simply repeating results from prior ME/CFS research. <https://t.co/fVaoj5hsg2> - Boy does our patient group of ME/CFS know this. We have been fighting against a very few 'elite' in the psychiatry world for decades. Only this year NICE have downgraded their research as poor, but previous guidelines were based on it. - "It’s also important to understand that scientists examining long-term consequences of covid aren’t starting from scratch. “Long covid research is still playing catch-up to ME/CFS research,” said Jaime Seltzer, director of scientific & medical outreach at MEAction… - Some researchers have spent decades trying to convince the public that ME/CFS is not a serious chronic illness but an emotional and behavioural problem that can be reversed with a positive attitude and exercise. - What an absolute joke this study is. As a two year Long Hauler recently diagnosed with ME CFS, I urge all patients to not take part in this nonsense. For the researchers: if you had actually done any research you would learn that most long haulers struggle to eat… - I totally agree. Responder rate is the variable we need in me/cfs (pots & mcas) research. Trad RCTs with focus only on the average &amp; effect size across the whole group just mean no meds come out good, bc diff meds help diff ppl. RCTs falsely assume we’re all the same. - I see that y'all are still repeating older studies that were already done for ME/CFS. By the time you catch up with what is already known, you'll probably have yet another name for the condition, and can start research all over again. Great way to never help people. |
| 3. Calls for more funding into ME/CFS research | - We need massive research programmes into both long Covid and ME/CFS, coupled with better information for doctors. But above all, we need something that currently seems a long way off. A government that gives a damn. #MEcfs #MyalgicEncephalomyelitis <https://t.co/7y0WmFK6Zh> - Petition: Urgent funding for research into Long Covid and ME/CFS treatments and cures https://t.co/YnbzW7YgD2 - Long Covid shows identical symptoms to ME/CFS and Chemical Injury. We all desperately need help to enable us to recover our health to the fullest extent possible. We need gov'ts to implement what we already know and do solid medical research on what we don't know. <https://t.co/8gp2IgEYwo> - More research needed, full stop. We need to understand the various conditions and physical causes and then understand which of these would respond to Paxlovid. If only money had been allocated to research ME/CFS years ago. It needs the same impetus as creating Covid Vaccine tho. - ME/CFS is one of the lowest funded diseases by NIH. #LongCovid often leads to it, but there are no biomarkers or much in the way of treatment because of this lack of $ for research. https://t.co/1VML92WceV - There is no commitment to ring-fence funding for biomedical research into ME/CFS to reflect the disease’s prevalence and severity, nor any attempt to redress the historic imbalance in funding which has disadvantaged those affected by the disease for decades. - You missed my point, JKR reinforced negative portrayals of diseases which are neglected for research funding, have been wildly and nastily misrepresented by media for decades. Never be represented in negative terms? People with ME/CFS have been serial gaslit, mocked and vilified - ME/CFS research is severely underfunded. Current NIH Funding: ME/CFS has $13 million in active NIH awards. HIV/AIDS has $3294 million in active NIH awards Dr. Hanson aptly puts these numbers into perspective (quote below). <https://t.co/Rw4DuyvMlu> - You’ve suspended the intramural ME/CFS study. You’ve neglected to include ME/CFS as a control arm in any forthcoming Long COVID research. There is no new funding for ME/CFS, which still amounts to several dollars per person per year <https://t.co/wRlSdwy2Wn> - Once this virus stops spreading &amp; is under acceptable control we need the NIH to fund real research concerning ME/CFS (Myalgic Encephalomyelitis/ Chronic Fatigue Syndrome). It is a life-altering and complex multi-system disease which has disabled over 2 million Americans. #MECFS |

# Table S4. Representative Tweets for the NICE ME/CFS Guidelines

| **Subtheme** | **Representative Tweets** |
| --- | --- |
| 1. Evidence used to support GET and CBT in the 2007 NICE ME/CFS guideline were flawed | - the PACE trial which has subsequently informed NICE guidelines on treatment for ME/CFS is a great example of completely ignoring the patient voice, as well as dubious research ethics (yet is still up on the Lancet). - NICE guidelines are based on flawed research. - No! CBT cannot fix autoimmune disorders. Full stop. Telling patients they have a psychosomatic or functional disorder when it is autoimmune is not only incorrect, it is cruel and unethical. See NICE guidelines - discredited PACE study that started the promotion of CBT for ME/CFS. - I'll take 'Who's the 'skeptic' most likely to be angry about the pending updated NICE Guidelines for CFS, the ongoing debunking of the work of Simon Wessley, & the broad recognition of ME/CFS as a specifically physical illness by medical science for 5 million Pounds, Alex.' - PACE scientists screwed up by including non fatiguing illnesses in their study (by using Oxford criteria which excluded PEM) and ppl with CF (symptom) were wrongly recruited. NICE guidelines (yes they could be MUCH better but at least GET is gone) state PEM is ME/CFS symptom. - The problem is the broad definition of ME/CFS in PACE. It makes results fairly meaningless. NICE guidelines are the problem. They treat PACE as more powerful evidence than it is. Let’s fight to change NICE in light of new evidence. A warning for GET. - The original NICE guidelines were that CBT would help patients address “unhelpful beliefs” about their illness. This CBT was not about acceptance or pacing, it was addressing a concept that ME/CFS was in our heads. - The PACE trial was also criticised in debates on ME/CFS in the House of Commons. Currently the NICE guidelines are under revision. - Unfortunately, the CBT/GET research is so flawed that it isn't trustworthy...Didn't you read the NICE guideline on ME/CFS?   So beside all the methodological flaws, PACE and the other trials are irrelevant to healthcare in the UK since the authors definitions of disease were not even close to NICE guidelines definition of ME/CFS. How could a study of CFS/Oxford possibly be relevant to ME/CFS per NICE? |
| 2. Changes to the NICE ME/CFS guideline were the result of prolonged efforts | - Controversial NICE guidelines on ME/CFS to be updated after pressure from patient groups. - taking literal decades of campaigning to get GET removed from the NICE guidelines bc it's killing us. - Any hope for challenge RE: NICE guidelines for people with ME/CFS based on flawed PACE trial? - Pls help! Call for scientific review of the #ME #CFS NICE guidelines (CG53) following the failure of the PACE trial. - Stop harming ME/CFS patients - take CBT/GET out of NICE guidelines NOW. Please sign below. - A petition to remove Cognitive Behaviour Therapy and Graded Exercise Therapy from the NICE guidelines for treatment of M.E. after the now discredited PACE study. - The NICE guidelines are as damaging to the health of patients with ME/CFS as UC is to the welfare system. Please help by signing this petition. - Patients submitted 5000 testimonies of harm caused by GET to the committee rewriting the NICE guidelines for ME/CFS. - Please sign petition re removing GET & CBT from NICE Guidelines. - Many thanks to everyone who signed this petition below re removal of GET & CBT from NICE Guidelines. We’ve reached 10,000, let’s keep it going and get to 15,000. - The NICE guidelines are being reviewed this year... ME/CFS charities involved. - Perhaps not surprising, but the stakeholder of GET and CBT have managed to halt the release of the new NICE guidelines, and NICE will now talk with them to agree on a path forward. How is this evidence-based, and not eminence-based? - US and NZ treatment guidelines for ME/CFS reject CBT and GET. New NICE guidelines were set to do the same yet a handful of influential medics with a vested interest in maintaining the status quo have the leverage to sabotage the entire process. #PublishThatGuideline - Many chronic illness patients have been severely permanently harmed by exercise “treatment”. NICE Guidelines were changed for ME/CFS after a lengthy battle. - An unprecedented third petition was launched to NICE “Stop harming ME/CFS patients - take CBT/GET out of NICE guidelines NOW” which asked that NICE immediately remove CBT/GET from the guidelines in the face of evidence of harm amounts to medical abuse & violation of human rights. - If you haven't seen it or signed it yet, #MEAction has a petition up, requesting that NICE guidelines be released as planned. - I'd like to ask people to please sign and share this petition asking for the updated NICE guidelines to be published so people like me with ME/CFS in the UK are no longer being offered harmful "treatments"!! - Everyone needs to read this to clearly understand why the old ME/CFS NICE Guidelines should never have existed and been accepted!! Welcoming the new guidelines on 18th! - The updated NICE guidelines for ME/CFS are out. GET is gone (which is an absolute triumph) and CBT is now a coping strategy not a treatment. I'll take that as a win. - We worked with ForwardME to ensure this guideline was led by #pwme. - BREAKING NEWS!! GET (Exercise) Removed from the UK's NICE Guidelines for #MECFS - It's finally happened, something patients have been waiting for many years. - The new NICE guidelines on ME/CFS that recommend patients stay active within their safe limits was generally welcomed by patients and a petition in support of its publication received 23000 signatures. - Finally, exercise intervention studies are precisely what ME/CFS patients have been trying to say for years have been making us worse and causing serious harm. That's why exercise intervention had been removed from the NICE guidelines. - Fantastic news! #GradedExerciseTherapy has been removed from NICE guidelines for ME/CFS patients. - NICE has set out the steps needed to put the recently updated NICE guideline on #MECFS into practice. We worked with ForwardME to ensure this guideline was led by #pwme. - Adam Lowe talks about his work on the NICE guideline committee as a lay member & highlights the amount of agreement between patients & clinicians. We owe a huge debt of thanks to Adam and all the lay members of the committee. #pwME #MillionsMissing - New NICE guidelines for ME/CFS are triumph of advocacy. A victory for those who shone a light on bad conclusions. Vindication for those of us who felt dismissed, discriminated and hurt by our medical system. We can hold our heads high today. We matter. #MECFS #pwME |
| 3. The 2021 NICE guideline confirms that ME/CFS is a biological disease | - most Neurologists think that my POTS and ME/CFS which was caused by a reaction to the vaccine is functional. This is despite overwhelming evidence to the contrary and new NICE guidelines. - This is evidence that the new NICE guidelines based on the IOM/NAM report is inadequate for #pwME. #MyalgicEncephalomyelitis is not an "exertion intolerance" issue. It is a neuroimmune disease requiring specialist care. Not behavior modification. - ME/CFS is placed under the Neurological Conditions section on the NICE guideline. - ME/CFS is now recognised as a disease and CBT cannot cure it. In the NICE guidelines. - A brief overview of symptoms, diagnosis and treatment based on the 2021 NICE guideline. Its not being tired all the time, patients have an energy impairment that dramatically limits their activity. - Finally, people with ME/CFS have recognition in the new published NICE guideline that their symptoms are physical. - The recent NICE guidelines have reversed previous statements about [ME/CFS] being psychological and now acknowledge that it is physiological. - The view that ME/CFS is a biomedical disease characterized by exertion intolerance has been affirmed by large reviews and position papers by major research and healthcare bodies. The new NICE guidelines are the latest example of this. |
| 4. The 2021 NICE guideline recommends against GET or CBT for ME/CFS patients | - the NICE guidelines changed in October (please see link below), graded exercise therapy in any form should not be prescribed to your #MECFS patients. - Yes really concerning push on exercise for LC, in UK being pushed even tho graded exercise therapy removed from NICE guidelines in 2021 for ME/CFS due to long-term & permanent harm. - current NICE guidelines: The results show clearly that cognitive behavioural therapy and graded exercise therapy are unsuitable treatments or management approaches for ME/CFS. - The Royal College of Psychiatrists promotes dangerous “treatments” for children with ME/CFS in contravention of NICE guidelines. graded exercise therapy (GET) can provoke relapse, which can in some cases become permanent. - NICE guidelines specifically mention that the Lightning Process should not be used in patients with ME/CFS. - Finally, exercise intervention studies are precisely what ME/CFS patients have been trying to say for years have been making us worse and causing serious harm. That's why exercise intervention had been removed from the NICE guidelines. - The new NICE Guidelines for ME/CFS state that the Lightning Process should NOT be offered as a treatment for it. Also, they confirm that there are currently no treatments for ME/CFS as the underlying pathophysiology of the illness is unknown. More biomedical research is needed. - The Nice Guidelines advise against exercise in ME/CFS. It can put the patient in bed for many months, even years. - The new NICE guidelines state very clearly that LP should not be offered to people with ME/CFS. - Please read the new NICE guidelines on ME/CFS so your clinics can identify any new ME/CFS cases to ensure they aren't put forward for exercise programmes that could cause them long-term severe disability. Exercise is no longer recommended for the treatment of ME/CFS. - Celebrating a true milestone for the ME community today with @MEActNetUK & @meactionscot! GET is gone from the NICE Guideline! The recognition that Graded Exercise Therapy causes significant harm & should not be offered to #pwME is a huge step forward. - The newly released NICE guidelines for ME/CFS is very firm about rejecting hysteria-based GET, CBT & The Lightening Process. - You’ve correctly noted ME/CFS is a common comorbidity of POTS, but then the video recommends exercise as a POTS treatment. Needs to be emphasised that exercise is usually harmful to people with ME/CFS (regardless of body orientation), see UK NICE guidelines. - Good coverage of the new NICE guideline on ME/CFS in the Isle of Wight News. It's good to see the message that GET is gone is spreading throughout the country. - The new NICE guideline on ME have banned GET and any other forms of graded activity/ exercise and curative CBT for ME/ CFS because it was found the evidence for it was low or very low and there is evidence of harm. - The massive problem with this, is that (at least some) Long Covid patients describes PEM and burst of symptoms after exertion. Which is a hallmark symptom of ME/cfs, for which exercise therapy is contraindicated, and even dangerous. As indicated in the last NICE guidelines. - The 2021 NICE guidelines for #MECFS have a severity box that outlines the situation…"People with severe ME/CFS are unable to do any activity for themselves or can carry out minimal daily tasks only (such as face washing or cleaning teeth)." - The NICE guidelines recognise that even graded exercise is harmful to ME/CFS patients, pacing is the route to improvement. - During a recent appt my doctors mentioned he read the new NICE guidelines, and he acknowledged the potential harm of prescribed exercise for people with ME/CFS. Feeling very grateful today - Article suggests exercise and CBT can treat brain fog. If ME/CFS is the cause, exercise is dangerous and CBT ineffective as treatment (as NICE guideline recognises). - Exercise is dangerous for those with ME/CFS. NICE says CBT is ineffective. |
| 5. The 2021 NICE ME/CFS guidelines must be defended and promoted | - Don't let vested interests derail the review of NICE guidelines on ME/CFS! - Miller, Sharpe, Wessley et al have responded to the NICE Guidelines draft. Suggesting that NICE have not followed scientific advice. Gaslighting is happening here once again, suggesting political pressure. - Concerning that this should be coming from NHS England, whose leaders (shamefully) tried to block the NICE guideline publication on ME/CFS. - Brian Hugh's Blog breaking apart the 8 criticisms of NICE Guidelines for M.E by 48 of the usual suspects. I think it still highlights the self serving nature of these people & they still refuse to accept that M.E is more than fatigue. - They've done a rebuttal to NICE guidelines on ME/CFS where they had to disclose their conflict of interests. The list of COI was almost as long as the article they wrote. - These FND specialists appear to be closely aligned with the people who false claimed ME/CFS could be cured with CBT and graded exercise therapy. Recently both of these groups launched an attack the UK NICE guidelines on ME/CFS because it rated their work as very poor quality. - My worry is ME CFS patients will just be diagnosed with somatoform which will circumvent new NICE guidelines to not use GET CBT. - I learned the names of lots of common logical fallacies from this great post by @b_m_hughes about that yet-to-be-published howl of protest against the new ME/CFS NICE guidelines, signed by dozens of core members of the pro-PACE ideological brigades. - Would still query their claim that CBT has been shown to be an “effective intervention for CFS/ME”. Not according to whole ME/CFS NICE Guideline committee. - Regarding the NICE Guideline, there will be no change until the psychiatric lobby are completely expelled from their profoundly destructive involvement in Myalgic Encephalomyelitis. #MECFS #MillionsMissing. - The @MEAssociation has been contacting NHS trusts that still recommend CBT and GET as treatments for #MECFS, in contradiction to the 2021 NICE guideline. - Dangerous misinformation put out by @BBCLN that is in direct contravention of the NICE Guidelines for ME/CFS and has the potential to harm patients who think they can push themselves into recovery on the basis of that statement. Please issue a correction. - RE: his saying the ME/CFS NICE Guidelines are causing conflict, the only complaints are from the small group of BSP authors whose papers were downgraded to very low quality or low quality & a few of their friends wanting to save their careers. The global experts welcome the update. - The Royal New Zealand College of GP's and @goodfellowunit1 have recommended GET, CBT & the Lightning Process as treatments for #MECFS, ignoring the findings of the NICE Guideline review, which specifically warned against GET & LP. - Sadly me and many others sounded the alarm at the beginning. If you read what has happened with the release of NICE guidelines on ME/CFS and one young man’s battle to get them released. Now there are Drs fighting not to use them. - The people who built their careers on disabling, torturing ... even killing ME/CFS patients are extremely powerful in the UK and were not going to accept this [the new NICE guidelines] because they would have to admit their fraud. So they have decided to play dirty. - Psychologists / psychiatrists have for too long held total sway over ME/CFS at the very highest levels of the NHS and they will not accept the new NICE Guidelines so are going on 'strike' to stop them. - See whinge in BMJ a few weeks back from the psychosomatic pushers trying to overturn the 2021 UK NICE guidelines for ME/CFS (that were based on detailed evidence re PEM and dangers/ineffectiveness of GET & CBT) - I just bloody knew it. Medical professionals are going to ignore nice guidelines and rebrand GET, CBT, to try to continue pushing us down the wrong road. Be careful everyone with #ME/CFS. WATCH OUT for anything like, improving activities, gradual activity increase etc. - The Mayo Clinic refuses to abandon the GET/CBT paradigm for #MECFS. They refer to the discredited NICE guidelines and the Cochrane review on exercise for ME/CFS. |
| 6. 2021 NICE ME/CFS guideline may help people with long COVID | - Let's hope the recent NICE guidelines changes on ME/CFS protect those with Long Covid from going through the hell that people with ME/CFS have gone through in terms of gaslighting and medical abuse. - If COVID patients meets diagnostic criteria for ME/CFS then need to be extremely careful with exercise prescriptions as there is a significant risk of harm. NICE guidelines in process of being updated but suspended due to pandemic. Current 2007 guidelines out of date & harmful. - should review the new NICE Guidelines for ME/CFS and use this approach to treat patients with Long Covid as they have Post Viral Fatigue Syndrome (PVFS). - lots of crossover with Long Covid & ME/CFS - big thing recently on graded exercise therapy harming & permanently disabling ME sufferers & now against NICE guidelines (shocking took so long to happen). NHS Your Long Covid Recovery not appropriate for LC & could encourage it. - Discussions will include Long Covid/Post Covid19 and the overlap with ME/CFS, the draft NICE guidelines. - The retraction of Graded Exercise Therapy in the draft NICE guidelines for ME/CFS is also good news for people with #longcovid #longhaulers #postcovid who are at great risk of harm too. - I am sure the CBT/GET services and researchers are worried about losing ME/CFS patients with the review of the NICE guidelines on ME/CFS. They will look to the #longcovid patients to mitigate the loss. - Are you unaware that half of Long Covid cases meet #MEcfs diagnostic criteria? (As such should get a ME/CFS diagnosis). Exercise is contraindicated for M.E/CFS (NICE guideline) - Just as it isn’t the answer for ME/CFS as per updated NICE guidelines (we’ve finally been listened to). My ME has worsened significantly because I exercised and pushed through the pain and fatigue. I hope LC patients won’t be told to do GET and encourage to rest/pace. - I have ME/CFS and probable long covid. I think it's crucial people are advised to rest and not push through or exercise after any virus to prevent post viral fatigue. The risks of exercise have finally been recognised in the NICE Guidelines for ME. - Long Covid patients could be in the bizarre position that it would be better for them to have an me/cfs diagnosis because at least then they can rely on the draft new nice guidelines. - It's ironic that UK #MECFS patients may soon be able to shelter from psychological abuse under updated NICE guidelines, while UK #longcovid patients may have no such protection. The strategy of some #longcovid patients of distancing from ME/CFS may turn out to be a big mistake. |

# Table S5. Representative Tweets for ME/CFS and Long COVID

| **Subtheme** | **Representative Tweets** |
| --- | --- |
| 1. There is a common biological cause of Long COVID and ME/CFS | - Maybe ME/CFS is associated with hypoxia for a different reason than microclots and hypoxia is the common denominator between many long covid and ME/CFS cases? - This research clearly demonstrates that the immunopathology of ME/CFS is dynamic and not fixed. It’s therefore likely that the few differences that have been found between ME/CFS and long COVID are an artifact of time since illness onset. - Mitochondrial disturbances in both Long Covid & ME/CFS are believed to contribute to loss of cell activity. - Stiffened blood vessels that could impair blood flows have been found in long COVID, ME/CFS and FM. - The brainstem regulates respiratory, cardiovascular, gastrointestinal, and neurological processes and its impairment can explain the overlapping symptoms of ME/CFS and long COVID. - ME/CFS patients without a history of COVID-19 infection also exhibit microclots & hyperactivated platelets similar to long COVID patients. - long covid and ME/CFS is characterized by kynurenine pathway issue - that means that instead of transform tryptophan in serotonine and melatonine - we will preduced to much quinolinic acid (with a difficulty to produce NAD+ from it) and hydroxykynurenine which are neurotoxic. - Some research showing vagus nerve issues in long covid. Could be relevant to ME/ CFS too. - The article says "a respiratory muscle dysfunction as a novel aspect of COVID-19 sequelae" but if the studies would join the ME (Myalgic encephalomyelitis) studies they could realize that both diseases have the same dysfunctions. - Voicing is a physiological process that involves nerves and muscles. I can't be the only one who reads the testament about exertion exacerbating dysphonia and connecting the dots between ME/CFS, LongCovid and dysphonia. - Like Long COVID, ME/CFS may involve a biological response that goes haywire when the body encounters certain infections or other environmental hazards. - Long COVID patients and those with chronic fatigue syndrome have significantly larger brain stems than healthy people. - In this paper, we provide evidence for frequent HSV-1 and EBV reactivation in both ME/CFS and long COVID patients and provide an experimental reasoning for potential cellular damage through herpesvirus dUTPase proteins. - High Intracranial Fluid Pressure, ME/CFS, Fibromyalgia and Long COVID. - Orthostatic Symptoms and Reductions in Cerebral Blood Flow in Long-Haul COVID-19 Patients: Similarities with Myalgic Encephalomyelitis/Chronic Fatigue Syndrome. - Queensland researchers find overlap in pathology of long COVID and chronic fatigue syndrome. - You might think of "Mitochondria are the powerhouse of the cell" [to] understand why damaged mitochondria leave ME/CFS long COVID patients so exhausted. - Brainstem volume changes in myalgic encephalomyelitis/chronic fatigue syndrome and long COVID patients are associated with pain, neuro cognitive dysfunction and PEM. - The inner lining of blood vessels (the endothelium) plays a crucial role in maintaining cardiovascular health. A recent study found that individuals with ME/CFS and those with long COVID had significantly impaired endothelial function. - Chronic inflammation, neuroglia dysfunction, and plasmalogen deficiency as a new pathobiological hypothesis addressing the overlap between post-COVID-19 symptoms and myalgic encephalomyelitis/chronic fatigue syndrome. - Metabolism seems to be broken in Long COVID, in ways similar to ME/CFS. |
| 2. Erroneous attribution of Long COVID and ME/CFS to psychological causes | - Myalgic encephalomyelitis, fibromyalgia, long covid, long vax, and the vaccine injuries killing young men around the world are NOT PSYCHIATRIC. - This article is full of nonsense about a 'psychological' & 'trauma' basis to ME/CFS & now LT Covid. It's a physical illness. We need a physical solution. - In terms of Long Covid and ME/CFS, there's definitely a concerted effort on the part of some powerful people to deny, minimise and psychologise. Which allows people to believe that recovery is possible, if you have the right attitude. - I had an enterovirus that did this to me. I went on to develop Myalgic Encephalomyelitis. I eventually got my taste (& smell) back but I never recovered. God help everyone with Long Covid - the same psych lobby has latched on with the same false illness beliefs. - A great article to read regarding ME/CFS and long Covid! I’m so pleased more research is being carried out. Patients need answers, not clinician who look at them as mental health patients. They are having physiological symptoms, not mental issues. - The psychologisers of ME/CFS continue with their comeback tour. Only nobody has bought tickets this time. It won’t be long before their harmful nonsense is consigned to the dustbin of history. Enjoying playing a part in this Long Covid. - A small group of psych lobbyists working for private insurance are trying to say ME & LC is psych so they can avoid insurance payouts. - QUACK psychiatrists who have been harming patients for decades. Stop this, just stop it. Haven't you harmed enough myalgic encephalomyelitis patients? Do you need to damage more people who have Long Covid with the same garbage psychobabble? - The Psychiatric domain works with the Medical Insurers globally to keep Long Covid as all-in-your-head needing graded exercise and CBT to stop malingering and stop claiming Insurance - our Profits are taking a hit! Just like they did for ME/CFS. |
| 3. If more research had been pursued for ME/CFS we would have effective treatments for Long COVID | - It is because NIH has not effectively accelerated ME/CFS research, that many people with Long COVID have been left without research-informed treatments or adequate care. - If more funding had been directed toward ME/CFS & post viral syndrome, we would be more knowledgeable about long COVID now. - If govts around the world had allocated more than a pittance to biomedical ME/CFS research over decades, the Long COVID fallout might not have been so severe. - I have been unable to work due to severe Myalgic Encephalomyelitis since 1998. Very little money spent on research. Now there are people with Long Covid who would've most likely benefited from such research. - If NIH had invested in ME/CFS research, we would have more answers. If we knew more, we could help people with Long COVID or perhaps even prevent it. Instead . . . well, here we are. - Now we have the devastating occurrence of long COVID, I hope and pray we’re reaching an end to the exploitation of post-viral illness ie ME/CFS sufferers and are heading for some long overdue legitimacy. It’s not our fault the money hasn’t been put into the research. - If the UK had invested properly in ME/CFS research, both long Covid and ME/CFS patients would not have to put their health at risk by travelling abroad for treatment. - If we'd taken ME/CFS seriously we'd likely have a lot more treatments available to pwLC and a whole lot less gaslighting for all of us. - Lot of peoples with long covid have ME and even is there is some difference we need to fight all together me/cfs is post viral too and suffer since one century. If they had doesn't ignored ME since the beginning we'll are not here today with this ridiculous lack of knowledge.... - There's a huge overlap between ME/CFS and Long COVID. If only people had cared decades ago to look into ME/CFS we might have had answers for Long COVID people. |
| 4. Current attention to Long COVID will benefit ME/CFS | - @NIHDirector You’ve neglected to include ME/CFS as a control arm in any forthcoming Long COVID research. There is no new funding for ME/CFS, which still amounts to several dollars per person per year. - As someone who’s had ME/CFS for 27yrs, riding on the coat tails of the left behind long covid sufferers is still like 1000x better than before. It’s hard for people to comprehend how ignored we’ve been by medicine for decades before COVID came along. - I have actually been an advocate for recognition of ME/CFS/PVS for some time. If you look back, lingering issues post a virus is nothing new and suffered by millions every year for a long time. Covid however is political and receiving so much more attention. Fair? - I hate to say it...but long covid is kind of a gift for us with me/cfs, because it has increased funding. - It's tragic that it has taken a pandemic to bring awareness to these disorders. Because of research into Long Covid, I'm hopeful for my son with ME/CFS. - We will learn so much about many systems and other conditions if we will do the research on SARS-CoV-2, COVID and long COVID. Ultimately could help many others, eg those with ME/CFS. |
| 5. Exercise and psychotherapy for long COVID and ME/CFS is harmful | - I dreamed I would recover from me/cfs but liaison shrinks at Kings & Oxford hijacked the illness and millions of us worldwide have been left to rot in hell or made worse by treatments based on fraudulent PACE trial. They are coming after Long Covid now. - In ME/CFS - an illness with huge presentational overlap with some long covid - 75% can’t work, 25% are house or bedbound. These statistics are probably so bad because for 40 years many National Health System doctors told people with ME to go away or fix themselves with exercise, which harmed the 250 000 affected. - Practitioners are using ME/CFS as a way of assessing post covid symptoms, but this is completely the wrong approach. Multi organ problems cannot be treated with CBT. - We observed a growing number of patients with long COVID who experienced adverse effects from exercise therapy and symptoms strikingly similar to those of myalgic encephalomyelitis (ME) - I direct messaged you about myalgic encephalomyelitis and covid19 long haulers. Don’t push yourself, it can make you worse long term. Talking, reading, any activity are all types of exertion. - New review shows that patients treated with CBT/GET are *less* likely to be able to work after this treatment than before. It also highlights that both treatments are unsafe for patients with ME/CFS or Long Covid. - The article talks about the likelihood of COVID survivors eventually being diagnosed with chronic fatigue syndrome or other chronic illness, and the fact that you can seem recovered and then exert yourself and be bedridden again. - Walking and running do not treat the underlying issue with ME/CFS or Long Covid. See NICE warning about graded exercise for LC patients and draft guidelines which say exercise should not be prescribed to ME/CFS sufferers. - graded exercise therapy is inappropriate & potentially deeply harmful for people with ME/CFS; potentially also #longcovid - advice is based on flawed research that needs retracting. - Long Covid is actually ME/cfs and there will be millions of cases. Sick for decades, gas lit by the psychs and abandoned by society. You will have to be careful rest of your life. Rest now. - GET (graded exercise therapy) has been removed from CDC & NICE treatment guidelines for #MECFS, so should not be used by #LongCovid researchers, or HCPs treating LC or ME/CFS patients. - Unless you have ME/CFS or Long Covid, in which case exercise can take you into total incapacity with severe intractable pain lasting for decades. Minimise exertion, rest, rest, rest. - Telling people with long covid to exercise is dangerous. Long covid is similar to ME/CFS. Pushing beyond limits makes sufferers worse and often bed bound. |
| 6. There is no cure for long COVID or ME/CFS | - I've been existing with ME/CFS for the past 29 years. It's a post-viral disease I got from Epstein Barr Virus (Mono). It's a living death. I've now had Long COVID since March of 2020. It's also a post-viral ME/CFS-like disease. There's no treatments nor cures. - The nagging symptoms long-haulers experience reveal a frustrating blind spot in medicine. Medicine hasn’t cracked how to deal with patients who have chronic syndromes like ME/CFS & Long Covid. - Should admit he has long covid, brain fog and will have to do what the millions of Fibromyalgia/ME/CFS do, take your meds and float out of the pain until Dr's find the answer. - Around 1 or 2% of COVID patients go on to get long COVID that does not go away. LC may be a form of ME/CFS. It is a lifetime sentence to being locked up in your own home for the rest of your life. No energy or mental focus to do anything. A fate worse than death, actually. - nothing helped me. HBOT [hyperbaric oxygen therapy] helped, but only while I was doing it. As soon as I stopped, any gains were lost. None of the supplements or medications made any impact. A lot of companies in the supplement/ well-being industry have made a lot of money from Long Covid and ME\CFS. - If what you have is Long Covid going on to ME/CFS, stay at home and rest, take care of yourself. There is no effective medical treatment, nothing that a doctor can give you that can make you better. - There's no treatment for ME/CFS after 6 plus decades ... what makes you think you'll do better with long Covid? - We've found out that long covid can cause you to develop chronic fatigue syndrome which is a disability with no cure. - There is virtually no treatment. However, recent studies show that the effects of chronic post-Covid-19 syndrome greatly resemble ME/CFS. - Long COVID is ME/CFS and there is NO CURE. - Well darling girl welcome to chronic fatigue syndrome. Most of us got it much later in life and covid was not the reason. You will need all the help you can get. This in almost all instances is permanent. |
